# To Track or Not to Track: User Reactions to Concepts in Longitudinal Health Monitoring

**DOI:** 10.2196/jmir.8.4.e29

**Published:** 2006-12-07

**Authors:** Jennifer S Beaudin, Stephen S Intille, Margaret E Morris

**Affiliations:** ^2^Digital Health GroupIntel CorporationBeavertonORUSA; ^1^House_nMassachusetts Institute of TechnologyCambridgeMAUSA

**Keywords:** User-computer interface, computers, handheld, ubiquitous computing, home monitoring, personal monitoring, personal tracking, personal health record, diaries, self-help devices, smart homes

## Abstract

**Background:**

Advances in ubiquitous computing, smart homes, and sensor technologies enable novel, longitudinal health monitoring applications in the home. Many home monitoring technologies have been proposed to detect health crises, support aging-in-place, and improve medical care. Health professionals and potential end users in the lay public, however, sometimes question whether home health monitoring is justified given the cost and potential invasion of privacy.

**Objective:**

The aim of the study was to elicit specific feedback from health professionals and laypeople about how they might use longitudinal health monitoring data for proactive health and well-being.

**Methods:**

Interviews were conducted with 8 health professionals and 26 laypeople. Participants were asked to evaluate mock data visualization displays that could be generated by novel home monitoring systems. The mock displays were used to elicit reactions to longitudinal monitoring in the home setting as well as what behaviors, events, and physiological indicators people were interested in tracking.

**Results:**

Based on the qualitative data provided by the interviews, lists of benefits of and concerns about health tracking from the perspectives of the practitioners and laypeople were compiled. Variables of particular interest to the interviewees, as well as their specific ideas for applications of collected data, were documented.

**Conclusions:**

Based upon these interviews, we recommend that ubiquitous “monitoring” systems may be more readily adopted if they are developed as tools for personalized, longitudinal self-investigation that help end users learn about the conditions and variables that impact their social, cognitive, and physical health.

## Introduction

### Background

Baby boomers, the cohort of adults born between 1946 and 1964, will contribute to a growing medical crisis in many industrialized countries. As demographics shift and lifespans increase, a larger percentage of adults will require medical care. The rising cost of medical procedures in combination with the greater numbers of people needing assistance will place an enormous strain on health care providers.

Ubiquitous computing and health technology researchers have responded to this developing medical crisis by proposing the use of home-based and wearable sensor technology to help people to assess their own health and that of their loved ones [1]. Research has already shown that health indicators typically monitored in clinical settings can be successfully deployed for longitudinal tracking in homes (eg, [2,3]). Numerous research efforts exist to develop systems that automatically detect activities of daily living (eg, [1,4-11]) and specific conditions, such as changes in gait. Sensors embedded in the home (and on mobile devices [12,13]) are proposed to collect longitudinal and contextually sensitive data that can then be processed to automatically detect important changes in behavior patterns caused by the onset of illness. Such systems usually collect data continuously or when someone is engaged in a particular activity of interest, such as playing a computer game [14]. Commercial systems use a small number of sensors per dwelling, typically motion sensors, to monitor variation from baseline movement throughout a home in the hopes of detecting serious conditions that lead to immobility (eg, QuietCare from Living Independently Group). These systems are popular for monitoring elders living alone [15]. Although in some work authors have advocated the use of these novel technologies for personal health tracking [16-18], the focus of much of this prior work on the use of ubiquitous sensing in the home is on health monitoring geared toward the health professional.

Unfortunately, both clinicians and potential end users of monitoring systems can be skeptical of the systems technologists are proposing. The technology faces significant barriers to adoption (see [19] for a discussion). Clinicians, for example, typically do not request assessment until a problem arises, and some clinicians limit predictive testing because of time, expense, and fear their patients will overreact. End users have reservations such as fear of being diagnosed with an illness with no known cure, fear of tests, fear of stigmatization, and fears about privacy violation or behavior being judged by family or clinical caregivers.

Therefore, rather than developing systems that only monitor health status and provide data to clinicians, an approach that tightly integrates traditionally separate areas of monitoring, compensation, and prevention [19] may reduce fears and increase adoption of home health technologies among end users. Monitoring systems that provide data of interest directly to laypeople may be received more positively and be more rapidly adopted outside of laboratory settings than systems that only track metrics of interest to health providers.

Monitoring technologies that are more widely available, such as manual logging tools and diaries, have been found to be helpful in improving communication with patients [20] and reinforcing lifestyle counseling [21]. However, logging can be difficult for patients to maintain over time [22], and data originally deemed useful from a practitioner's perspective may not be easy for users to apply to everyday situations [23]. Tracked data may need to have immediate applications and be particularly relevant from a user's perspective for these systems to be kept in use long term.

Home monitoring technologies face another barrier to adoption, a classic “chicken and egg” evaluation problem. To make a (statistically) convincing argument that home monitoring systems can provide useful indicators of early onset of disease will require studies where the monitoring technology is installed in many homes for long evaluation periods, most likely months or years. To justify the cost of a sufficient number of installations, however, will require evidence of the preventive health value of the monitoring systems.

Prior work has shown that end users often strongly believe they should keep their own personal health records in addition to those kept by their medical personnel [24]. It has been proposed that such records would be enriched by including data and subjective reports collected outside of the clinic [25], but to date, no research has been done to suggest what end users would choose to track and what value, outside of the clinic, tracking would provide.

In this work, we therefore focus on ways of developing longitudinal home health monitoring systems that provide high perceived value to end users. Our primary question is whether “monitoring” systems can be designed that might be adopted by end consumers for personal use—even by people who would not characterize themselves as sick or in need of monitoring by health professionals or other caregivers. The information these systems collect might be used to develop novel forms of personal health records, which have recently attracted interest [25]. We present qualitative results from interviews with 8 health professionals and 26 laypeople who were asked to respond to mock data visualizations for novel longitudinal home health and activity tracking technologies.

The data display interviews described later in this paper were motivated by a set of exploratory interviews conducted for a project to develop a novel cognitive performance tracking technology. We interviewed 11 US-based professionals in aging and cognition, including a gerontologist, a nurse specializing in geriatrics, a home nurse, a cognitive psychologist, an occupational therapist specializing in geriatrics, three neurology researchers, and three neuropsychologists. The interviews were unstructured and lasted approximately 1-2 hours in length.

During these initial interviews, we observed that experts had not yet fully considered the breadth and depth of patient states and activities that emerging technology will be able to monitor outside of the clinical setting. Therefore, the experts, particularly the clinicians, had difficulty generating specific ideas on how their practices could take advantage of nonclinical monitoring of health status indicators other than the already familiar indicators such as heart rate, weight, and blood pressure. Nevertheless, some experts did propose new health status indicators that could be tracked outside of the clinic, and these suggestions motivated us to continue this area of inquiry. They included “catastrophic reactions to mundane activities” (eg, becoming unduly anxious when calculating a bill), ability to multitask during cooking, and changes in speed of interaction with appliances.

### Objectives

Our exploratory interviews confirmed that we needed visual aids to help experts and laypeople understand how emerging technologies might be used for monitoring health-related status. Most clinicians and laypeople have had limited exposure to longitudinal tracking methods and devices. Clinicians, for example, are accustomed to evaluating patients based on periodic and limited clinic data. Laypeople may have experience using a bathroom scale, thermometer, or pedometer, but they are unfamiliar with the capabilities of emerging ubiquitous computing technologies. To address these barriers, we mocked-up data displays representing a variety of constructs for a hypothetical patient or family. These materials were used as a concrete focal point for structured interviews with medical experts and laypersons. The data displays are not necessarily proposed concepts but are instead a mechanism used to elicit feedback about longitudinal tracking ideas. They are intentionally diverse and provocative and are used as probes to elicit detailed reactions and self-reflection during interviews. Our design criteria for the displays and strategies we used to achieve these goals are listed in Textbox 1.

**Interview goals and examples of strategies for participatory design stimuli used to meet those goals**

**Table:** 

**Interview Goals**	**Examples of Strategies Used in Displays**
Invite reflection on longitudinal monitoring of particular variables and outcomes.	Many displays consisted of sequences of steps and multiple timescales.
Invite focus on the output instead of the mechanism of monitoring/tracking.	Technology depictions or descriptions were not included; some displays explicitly suggested that data are collected manually.
Encourage participants to model how they would respond if they had tracking data, including how they would interpret outcomes and what follow-up investigations they would conduct, if any.	Displays represented accumulation of data as though the tracking tools had been in use for some time; displays put focus on action of reviewing data, instead of collecting data.
Encourage participants to think and talk about themselves and their personal concerns, values, and preferences.	Axes on graphs were often not labeled to avoid fixation on data values. Participants were encouraged to talk about what the display would look like for them.
Encourage discussion about underlying issues related to tracking, rather than restricting feedback to evaluation of a particular idea.	Multiple examples on one display could be quickly turned on and off; we deliberately restricted the set of metrics for each example to encourage brainstorming about additional metrics.

The displays were designed to encourage participants to role-play scenarios. Participants were instructed to envision themselves or their patients self-monitoring and analyzing the personalized health data. The displays were intended to illustrate capabilities of longitudinal monitoring (Textbox 2) to which participants often alluded but had difficulty discussing in more detail without stimuli.

**Home health tracking concepts that people may have difficulty discussing and relating to their personal situations and concerns**

1. Data collected over time can reveal patterns of change.
2. Context can be used to interpret reasons for change.
3. Comparisons can be made with population norms, personal goals/estimates, and peers' values.
4. Quantitative data can be used in combination with qualitative data (eg, journal entries).
5. Multiple metrics can be applied to assess health and behavior change.
6. Data can be used to motivate by highlighting the extent of a problem or documenting progress.
7. Data can be used to problem solve and evaluate interventions.
8. Data can be subjectively reported or objectively observed.
9. Data can be reviewed at specific times and locations.
10. Data can be organized in ways other than by time.
11. Data tracking may not be constant, instead triggered by directed investigations.
12. Data can be reviewed in isolation or in relationship to other variables.

## Methods

We developed 17 mock data display examples, many of which represented multiple tracking concepts. Initially, the displays were developed on paper, but to facilitate long-distance interviewing, they were converted to interactive Web pages using Flash (Multimedia Appendix 1). Most of the examples could be developed using emerging ubiquitous computing and/or wearable technology. A few (eg, an example that assumes the computer can detect linguistic “hedges”) would not be possible to implement at this time. Four of the displays are described in more detail below. We also developed sorting exercises to provide participants with a way to express tracking priorities, complementing the more open-ended feedback from the displays; they are described in the final Methods subsection, “Sorting Exercises with Laypeople.”

### Weight Displays Example

The weight display series was used to address possible monitoring strategies to facilitate a weight loss goal. The complexity increases sequentially. In the first display, one data point is available: the patient's weight at the clinic. This value is compared with norms for gender and height. The second display (Figure 1a) adds the patient's weight from a clinical visit one year ago, indicating that the currently overweight patient has actually succeeded in significant weight loss. The third display (Figure 1b) suggests that the patient has been recording his or her weight each month throughout the year; the patient's weight was steady, started to steeply decline, and finally reached a plateau. The seasonal context indicates that decline began in January. The next in the series of displays (Figure 1c) switches metrics to show pedometer readings plotted against exercise goals for each week. The final display (Figure 1d) depicts the addition of qualitative description: photos taken on “good days,” when the user's steps greatly exceeded the goal for the given week, and “bad days,” when the user's steps significantly failed to meet the goal for the given week. These “photos” are left blank, but the participants are told that they could capture anything experienced on those days: people encountered, food eaten, places visited.

**Figure 1a:**
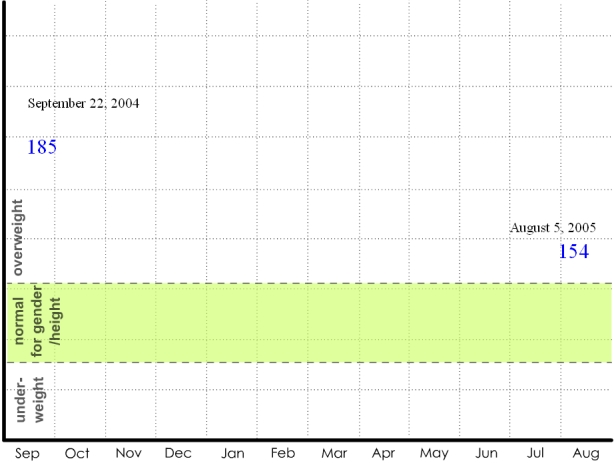
Weight displays: (a) traditional clinical weight data

**Figure 1b:**
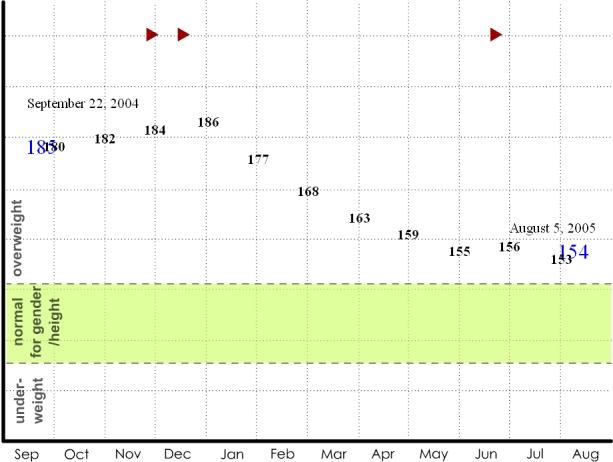
(b) home monitoring weight data

**Figure 1c:**
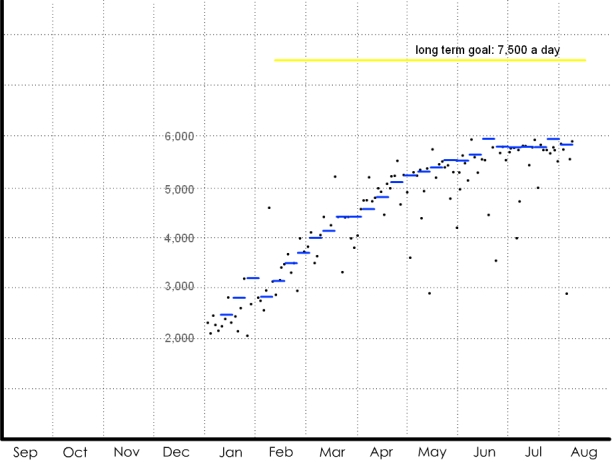
(c) pedometer readings

**Figure 1d:**
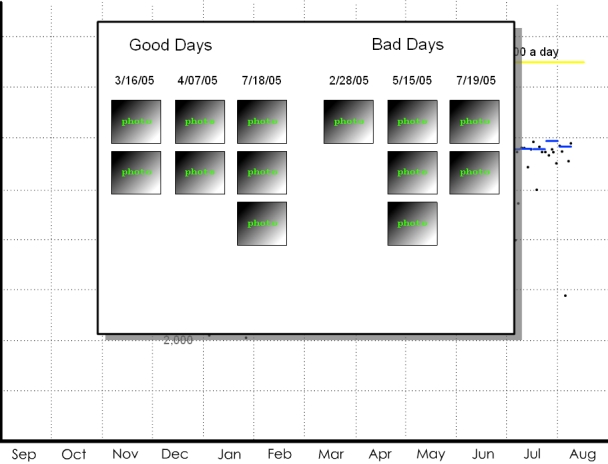
(d) qualitative data—photographs

The displays tell a story that begins with the more familiar and accepted clinical metrics and gradually transitions to richer and more diverse sources of data, concluding with a qualitative display that could provide information about a variety of life factors. Displays such as these were used to help participants understand the potential of home health tracking technologies in a step-by-step fashion. In this example, the interviewer depicted a typical patient struggling with weight who committed to change (New Year's resolution), used a pedometer to record and motivate progress, and finally reached a plateau—a point which drives many back to poor habits because their efforts are not reinforced. The interviewer described how extra data, such as the analysis of contextual factors and qualitative image data, could help the patient-physician pair work through this apparent impasse. The tools for this weight example are all commonly available: a bathroom scale, a pedometer, and a digital camera. This display series illustrates concepts 1-7 in Textbox 2.

### Scenario Displays Example

The weight displays example was developed specifically to help health professionals transition from familiar patient charts to more novel tracking concepts. To achieve a similar goal with laypeople, scenario displays (Figure 2) were developed showing extensions of familiar media associated with recording and reflecting on personal data. With the journal display (Figure 2a), participants were asked to imagine that they had the time and discipline to keep a richly detailed journal, which might include information as diverse as memorable moments and snacking events. With the health snoop display (Figure 2b), participants were asked to imagine that they could hire someone to follow them around for a week, nonintrusively making observations about such things as work habits and missed opportunities for exercise. These examples explicitly sidestep the data collection issues in favor of focusing on the basic ideas of subjective and objective recording and analysis of behavior (Textbox 2, #8). Other displays included data overlaid on a grocery receipt (Figure 2c), overlaid on a medicine cabinet mirror, and communicated by email or voicemail. These examples focused the participant on the action of reviewing and applying data (Textbox 2, #9), rather than on the collection of data.

**Figure 2a:**
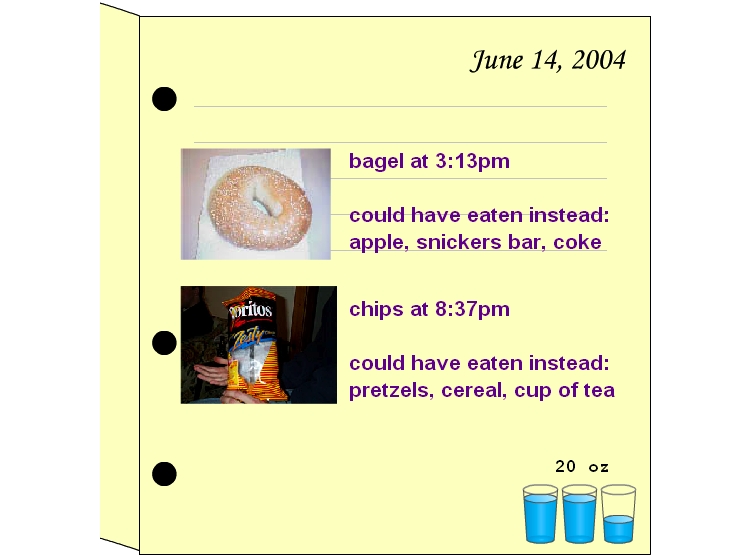
Scenario displays: (a) journal display

**Figure 2b:**
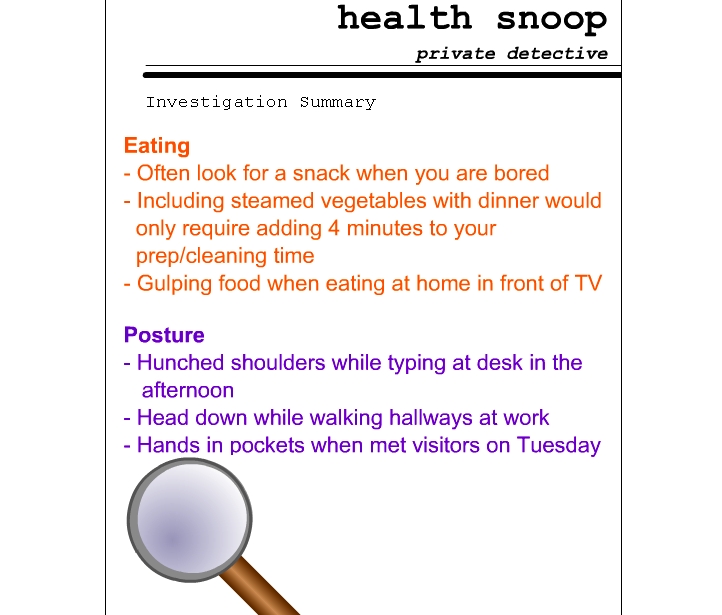
(b) health snoop display

**Figure 2c:**
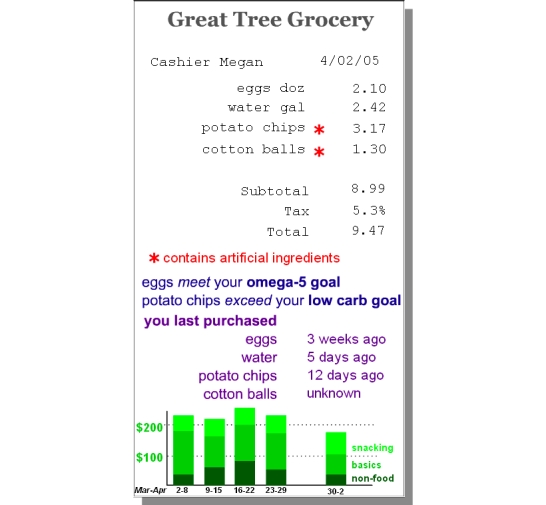
(c) grocery receipt display

### Body-Based Displays Example

The Web-based displays permitted customized interviewing, where a basic display template could be overlaid with multiple data examples by checking options on the screen. The body example (Figure 3) presented tracking data organized according to associated body regions (Textbox 2, #10). By checking the options in the bottom right corner and clicking on parts of the body, the participant could review ideas for tracking skin changes, history of exercise, activity levels, and aches and pains. Some displays showed information that would be tracked by self-report (eg, headaches). The goal with multiple examples for each display template was to encourage brainstorming, whereby the participant could feel comfortable giving gut reactions to specific ideas, while getting acquainted with deeper tracking concepts (eg, Textbox 2, #11) that they could then discuss.

**Figure 3a:**
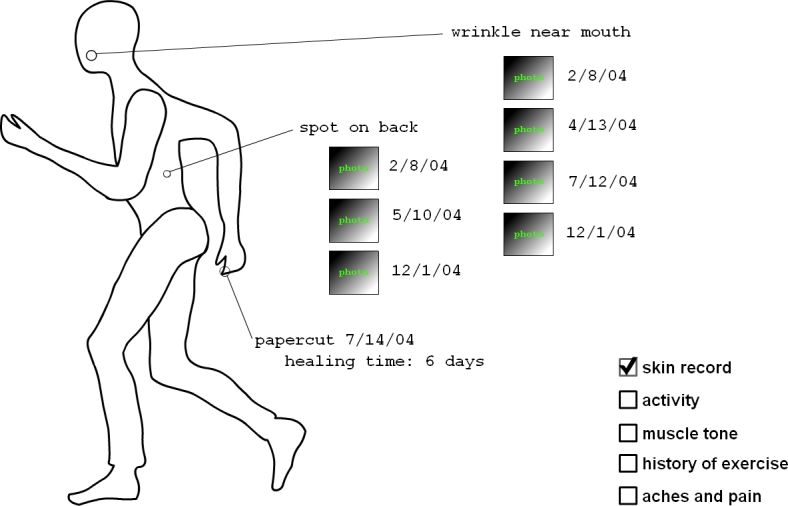
Body-based displays on (a) skin changes

**Figure 3b:**
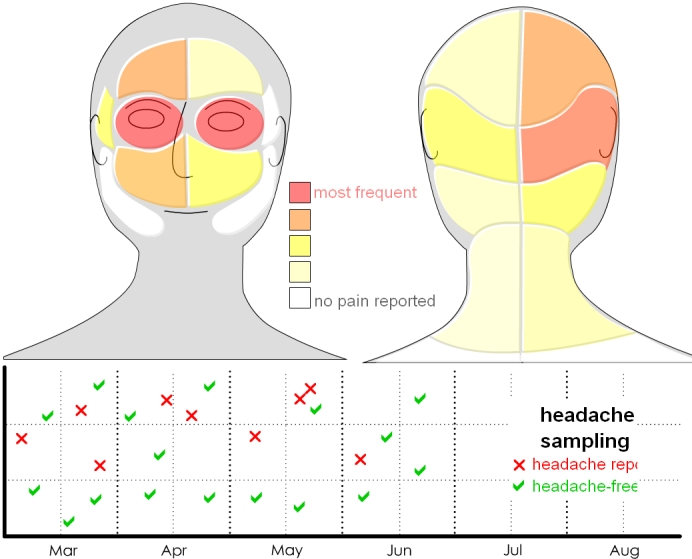
and (b) headaches

### Time Scale Displays Examples

More traditional graph-style displays were used to depict monitoring across multiple time scales (days, weeks, months, or years) (Figure 4). Y-axis units were not labeled to limit fixation on absolute data values that may not be salient for a particular participant. For example, time spent watching TV (Figure 4a) was represented with bars of varying heights, but there was no label of exact time. Variables of interest could be explored in isolation (eg, mood over time) or in relationship to other variables (eg, mood with respect to TV watching) (Textbox 2, #12)—the complexity of analysis depended on how many variables a participant checked in the screen display. On some displays, data could be compared with self-estimates (eg, your estimated TV watching) or group statistics (eg, average TV watching for your age group) (Textbox 2, #3). The years example (Figure 4b) was used to ask participants about variables that may be sensitive to major health changes.

**Figure 4a:**
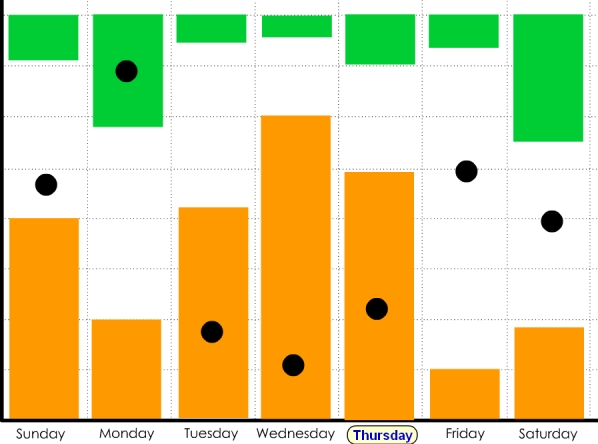
Time scale displays on (a) time spent watching TV (orange: TV watching; green: time spent outside; black dots: mood rating)

**Figure 4b:**
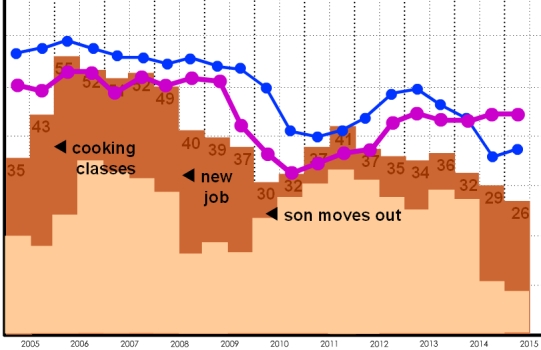
and (b) variables that may be sensitive to major health changes

### Interview Procedure with Health Professionals

We interviewed eight health professionals whom we identified through a working group on patient-centered health care at the Massachusetts General Hospital and through word-of-mouth. In contrast to the first set of experts from the preliminary interviews, many of these professionals treated healthy individuals and expressed a desire to shift to a coaching style doctor-patient relationship. They included a personal trainer, two social workers, three general practitioners, and two general practitioners who were cardiac specialists.

The interviews were 1-2 hours in duration. For all but one interview, the paper-based materials were employed; one participant responded to the electronic-based materials. The health professionals were shown 5-10 displays, starting with the weight displays, chosen because they depict a kind of patient-directed tracking with which health professionals may already have experience (ie, weight and pedometer steps).

Participants were instructed to imagine that the displayed data were collected in the patient's home and returned to the patient for personal reflection and conversations with a doctor or family member. They were reminded that the data in the displays do not necessarily reflect the capabilities of current technologies. For each example, they were asked to comment on how the information might be interpreted and applied by a patient, what it would be like to talk with a patient about the information, and what additional information would be beneficial to provide. They were encouraged to voice any concerns they might have about the collection and application of the data from their perspective as a health care provider.

### Interview Procedure with Laypeople

We recruited 21 participants via postering, mailing lists, and word-of-mouth. All were from the United States. Participants volunteered their time without compensation for a protocol approved by our institutional human subjects review board. Included were 15 women and 6 men, ages 40 to 66 years (mean = 51; SD = 9). Although our target demographic was people in middle life, we additionally interviewed five individuals over age 70 for comparison. Three participants were close affiliates of one of the authors. Seven participants were from the local area of Cambridge/Boston, MA. The remaining participants were interviewed over the phone and represented 10 states.

The interviews were 1 hour in duration. In all interviews, the Web-based data displays were employed. Participants were shown 4-11 displays, typically starting with the journal example, chosen because it depicts a kind of tracking with which laypeople may already have experience (ie, diary or journal entries). They were instructed to imagine that the displayed data were collected in their home and returned to them for personal reflection. They were reminded that the data in the displays do not necessarily reflect the capabilities of current technologies. For each example, they were asked to identify content or qualities of interest or concern, how they might personally apply or use the information, and what additional information would be beneficial to have. The interviews were transcribed (masked transcripts with research notes are available in Multimedia Appendix 2).

### Sorting Exercises with Laypeople

The displays were used to provoke discussion about health concerns that people wanted to investigate and the ways they could imagine conducting such investigations. We also wanted to quickly elicit specific ideas and rankings for concepts to track. We developed a list of 60 sample constructs that could be tracked over time using current or proposed tracking devices. These constructs represented multiple levels of inference or granularity and were selected to cover a diverse set of domains, including social interaction, cognition, physical activity, and physiology. Examples include “mood self-rating,” “blood pressure,” “tossing and turning,” and “time spent in the car” (see Multimedia Appendix 3 for the complete list).

After discussing the displays, the layperson participants in middle life (n = 21) spent 15 minutes doing the sorting exercises. The health professionals and five older adults also did a sorting exercise, but in a more open-ended manner, so for clarity, their results are not included here.

For interviews conducted face-to-face, participants were given a set of hand-written index cards to sort. For phone interviews, participants were directed to a Web page with a list of the constructs. Participants were asked to talk aloud while they sorted to indicate their choice (yes, no, or maybe) and comment about particular reasons for their decisions. Participants were asked to quickly sort the constructs according to whether they would personally want to track each over time. A second sorting exercise, where participants sorted with a particular investigative goal in mind, gave participants the opportunity to reconsider their initial gut reactions and express more focused applications for tracking. Participants were asked to select from a list of personal health areas that they might want to better understand and positively affect using tracking. The list included eating choices, family relationships, mental sharpness, mood, physical activity, and stress. Participants (n = 20) then sorted the set of constructs again, according to whether they would personally want to track each to understand their selected goal. One participant (under age 70) did not do the investigation sort due to time restrictions on the interview.

## Results

We now review a few specific reactions from participants to the data displays and summarize the perceived benefits and limitations of health tracking from the perspective of the practitioners and laypeople. We also present variables of particular interest to the interviewees, as well as their specific ideas for applications of collected data.

### Interviews with Health Professionals

In examining the body display (Figure 3), an interviewee who is a general practitioner first identified a benefit to the patient in having this type of data; he suggested, “Perhaps it could arm the patient so they can go in and more effectively communicate their worry [to a practitioner].”

**Longitudinal tracking benefits and concerns perceived by health professionals, summarized from the 8 preliminary interviews and the 8 materials-based interviews**

**Table:** 

**Professional Perceived Benefits**
Longitudinal data could be used to motivate and reward progress toward long-term goals (eg, exercise, nutrition); shift to life-goal assessments/recommendations, shift to “contracts” for change. (n = 6)
Longitudinal data can help doctors broach sensitive topics (eg, health of social relationships). (n = 4)
Longitudinal data can help doctors ask interesting questions and initiate a dialogue with the patient (n = 3); data can help patients communicate concerns. (n = 1)
Data collected outside the clinic is likely to be more representative of the patient's actual health. (n = 2)
Longitudinal data could be used to evaluate the success of interventions. (n = 2)
Longitudinal data may reveal that some constructs are more cyclic and context-based (e.g. mood corresponding to hormone cycles). (n = 1)
Longitudinal data could be used to detect the precursors of problem behaviors, such as eating disorders. (n = 1)
Longitudinal data can help the doctor make the most of a limited clinical visit. (n = 1)
**Professional Perceived Limitations**
Family physicians will not have the time or willingness to evaluate the data. (n = 4)
Most of what could be monitored either is not useful or is known already by the doctor, home nurse, caregiver, or patient. (n = 3)
Patients lack the ability to be self-aware about cognitive changes. Denial is too strong to overcome and, when serious, the cognitive impairments themselves make awareness difficult. (n = 3)
Patients may feel like their privacy or self-image is threatened. (n = 3)
Data will not represent more meaningful life pursuits (eg, becoming more self-aware, artistic advancement, enriched mental life). (n = 2)
Current generation of older adults is not interested in taking more responsibility for health. (n = 2)
Patients may become obsessed with data or may take a too narrow view, rather than approaching health holistically. (n = 2)
Data demonstrating decline will be depressing or disempowering. (n = 2)
If viewed over too long a time period, data will encourage a nonadaptive self-image. Instead of recognizing that one's current capacities are suited for the current context and meet basic life goals, one may focus too much on how one is not the person one used to be. (n = 1)
Patients may make snap judgments about contextual factors associated with health changes (correlation is not causation). (n = 1)

But he then began to imagine a counterproductive result, role playing the following interaction:

The patient who would bring this in, I would not look forward to seeing as a doctor…. [This kind of data] almost lets the patient live and dwell in something that you would rather have them just say—“I have these reoccurring headaches”—great, thank you, now how's the rest of your life going? It obscures rather than clarifies, in the conversation, they become too focused on the minute.

Although this health professional strongly supported giving patients more control over their health through information, he worried about patients becoming obsessed with data and ignoring the larger issue of holistic wellness—a trend he has seen with medical professionals who over-rely on technology-generated data.

One of the interviewed social workers consults with her patients to identify resources and opportunities to establish healthy patterns. Reacting to a display graph depicting “kitchen cabinet access events” over time, she first noted that it would be useful in her work with patients with eating disorders. These patients often display food-search behavior as a precursor to destructive eating episodes. She envisioned using the data to draw patients into discussions about this behavior pattern and strategies for behavioral change. She then noted a second application: identifying “tea and toast ladies”—older adults who have over-simplified their diet following cognitive, physical, or emotional impairment.

The displays shifted interview foci from possible monitoring applications to personal scenarios, such as “What would it be like to have a patient bring me this data?” Although many of the presented ideas represented a departure from clinical metrics, they evoked a generally positive response and elicited more detailed feedback on using longitudinal data to develop individualized treatment plans and support patient-doctor communication. With the exception of one social worker who specializes in geriatrics and who was pessimistic that additional information or greater awareness would be relevant for late-stage health problems, the interviewed professionals thought that longitudinal tracking tools targeted to patients would be beneficial for the type of health practice they would like to have. However, several noted that the typical medical clinician would not be receptive. One participant responded, “A presently trained and configured family…doctor would think it was junk. They would go, 'Well, that's interesting, I got 9 more minutes.'”

An additional concern that appeared in multiple interviews was that the data should be presented in ways that do not force evaluation by comparison with norms, baselines, or externally determined goals or by presentation in a “report card” format. That said, some professionals indicated a desire to use data to motivate patients, one even arguing that the “report card” format was desirable.

We have summarized some of the health professional's key perceived benefits and limitations about longitudinal tracking in Textbox 3.

### Interviews with Laypeople

At the beginning of the interview, participants were asked what they currently monitor or track. A man, age 40, who was working in a technology field, was a serious health tracker, manually recording multiple variables related to exercise, diet, and mood, including satisfaction ratings for leisure activities. Another man, age 40, working in a technology field, was a committed blogger, recording his intellectual process for work and maintaining a personal “kids” blog for his family. These participants were unusual in their existing dedication to tracking. However, all participants engaged in some type of monitoring (such as for weight, blood pressure, water drinking). Some kept journals (n = 9), tracked expenses (n = 2), kept annotated calendars (n = 7), and compiled lists of movies/books they watched/read/recommended as a backup for memory (n = 5). Several owned pedometers (n = 7), but no one was using them to track and review activity over time, citing reasons of forgetting and reluctance to exert the effort to record values.

We have summarized and categorized some of the laypeople's key perceived benefits and concerns about longitudinal tracking in Textbox 4. Here we describe a few specific reactions from participants to the data displays. For example, a woman, aged 66, who was working in an administrative position, initially responded to longitudinal tracking with disdain, asserting that she “already knew” the information it could provide and that anything she did not know would be better addressed by working with a professional. “It is interesting to me that you and the folks you are working with had the idea that people would like to have this information.” After being introduced to the years example, as she expressed her reactions out loud, she gradually shifted perspective to consider the value of passive monitoring to establish a baseline and identify change. She did not want tracking as a tool for reflection and behavior change, but decided that background monitoring would be beneficial as she gets older. She did not want to use “it as a tool for understanding [her] life right now, but [to] monitor and anticipate change as [she is] aging, as an early warning to go to a doctor .... You've flossed every day for the last 12 years and in the last month, you've skipped 5 days, so what's going on?”

A 45-year-old man, who was a working professional, had re-purposed technologies to do some of his own tracking investigations. Prompted by a personal disagreement, he had recently reviewed years-old emails to gain perspective on the evolution of a relationship. He kept a personal calendar on his cell phone and noted, in reaction to one of the displays, that he is more conscious of commitments that are hard for him to keep (addressing finances) because he has a record of postponed appointments. He was strongly positive about an example that provided information about daily rhythms, believing that even without knowing what to look for, he would still find interesting patterns. “I think I might discover something…maybe correlate this with the journal to see how sleep patterns affect your overall mood or…amount of time you spend with other people affects your outlook.” He worried though that having to describe his current mood states might amplify negative feelings (eg, saying you are sad makes you sadder). He was interested in how tracking could help him find more personal time. As a father, he could imagine using the group data to ground family discussions about issues such as bed time and together time, but was concerned that, in the end, the data might work to confirm and entrench opposing opinions. “So everyone will find the facts to support their interpretation of what's happening and the more facts you gather, the more it tends to just cement the positions of the people. That's been my experience.”

A woman, aged 58, who was recently retired, wondered whether she could use longitudinal tracking to determine the amount of structure in her day that would give her the most satisfaction. She was interested in carrying out a variety of investigations, wanting to understand why she made qualitative judgments about a movie or a meal and what factors contributed to her mood. “It's like oh, today was really good—well, why was it good? Was it because of some really trivial compliment that you were given early in the morning, or is it because you accomplished something…. Was it just your blood sugar in the morning?” She identified eating habits as a behavior she would like to affect and expressed a desire for tools that would let her evaluate her personal degree of success with recommended interventions, such as drinking water to curb hunger. Additionally, she wanted data about her menopausal symptoms (eg, mood, memory problems) to persuade her reluctant physician to prescribe hormone therapy.

Overall, seven participants were reluctant to track, often stating that they “already know” sufficient information about their health and activities and preferred solutions (such as reminders) and expert-mediated care. Nevertheless, they each indicated that some tracking would be useful, either to establish a baseline and detect troubling changes or to address very directed investigations related to exercise or diet. Thirteen participants responded positively to multiple tracking ideas, particularly those related to behavior change and time management, but were somewhat conservative in proposing novel tracking investigations. Six participants were strongly positive, expressing comfort with exploring patterns and relationships in data, examining data on multiple time scales, and initiating involved or novel tracking investigations. Participants often qualified positive reactions by saying that the tracking idea would be just “fun” or interesting, but not necessarily valuable. They often initially focused on isolated and simple investigations (eg, frequency of headache pain), but with prompting, generated ideas about possible contributing contextual factors (eg, amount of sleep, shoes you are wearing) that when tracked could give them opportunities to problem solve. When participants strongly connected with a tracking idea, they often cited significant life events (eg, the 58-year-old's recent retirement and experience with cancer) as giving them a deeper appreciation for the contextual factors related to general wellness. As the interview progressed, participants frequently identified behaviors or variables that were difficult to self-monitor (habits, behaviors done in combination with others, accumulation of subtle changes, personality variables, too familiar routines or factors) and were therefore ideal candidates for technology-assisted tracking.

**Longitudinal tracking benefits and concerns perceived by laypeople, summarized from interview transcripts**

**Table:** 

**Layperson Perceived Benefits** **Tracking may be useful for…**
**Supporting behavior change:** Motivating/enabling change (n = 7); understanding cause and effect (n = 5); reinforcement, forcing to face issue (n = 5); evaluating success of intervention (n = 4); time management (n = 4); inspiring problem solving (n = 3); achieving “optimal state” or “peak condition” (n = 3); permitting trade-offs (since I've been good all week, it's okay to cheat) (n = 2); giving a sense of accomplishment (n = 2); identification of need for replacement activity (I was more active when I had kids, I had a more regular routine when I lived by the beach) (n = 1); measuring progress (n = 1)
**Making patterns more evident:** Understanding habits, traits, behaviors (n = 6); identifying factors that affect you positively or negatively (n = 5); identifying sources of distraction (n = 3); rethinking major life patterns (n = 4); identifying valuable improvisations (n = 1); understanding consequences of life-work balance (n = 2)
**Monitoring health:** Providing material for conversation with doctors or family (n = 11); determining seriousness of problem (n = 4); identifying troubling changes (n = 4); in place or available for later problems (n = 3); establishing a baseline for later comparison (n = 2); understanding nature of change (gradual versus sudden, onset) (n = 2); early detection, chance to cure, make plans for compensation (n = 1)
**Challenging or validating beliefs:** Getting perspective (n = 6); giving a sense of control (n = 5); promoting a positive self-image (n = 5); validation of subjective feelings (n = 1); peace of mind (monitoring parents) (n = 1)
**Providing a record of events:** Jogging/strengthening memory (n = 6); backup for memory (n = 6)
**Providing entertainment/Supporting social interaction:** Feeding curiosity, fun, interesting (n = 8); serving other or dual goals (reminders, communication) (n = 4); addressing physical appearance (n = 1)
**Layperson Perceived Limitations** **I wouldn't want to track (a variable or in general) because tracking would…**
**Not apply to me:** (eg, smoking, alcohol drinking, pets) (n = 26)
**Not provide new information:** (ie, “I already know this”) (n = 22)
**Not provide valuable information:** Something/someone already takes care of that (n = 6); unimportant or irrelevant (n = 5); just want a solution, not more information (n = 3); no change needed—functioning okay even though impaired (n = 2)
**Provide too much information (information overload):** Too focused on minute details, missing the big picture (n = 8)
**Threaten self-image:** Lead to denial/feeling threatened (n = 9); would feel criticized (n = 5); don't want to think about (eg, hate exercising, don't want to spend time thinking about exercising) (n = 2); lead to uncomfortable sense of competition (n = 1)
**Not provide actionable information:** Concerned about becoming discouraged or depressed (n = 7); frustration if don't know what to do or can't do anything (n = 7)
**Lead to social conflict:** Forcing involvement between family members (n = 5); fueling conflict and entrenched opinions (n = 3); other household members would not want to participate (n = 3)
**Promote obsessive or unhealthy reactions:** Living in the past (n = 3); will make too self-focused (n = 3); becoming obsessed (n = 3); becoming dependant and not thinking for self (n = 1); may use as an excuse not to change behavior (n = 1); will ignore after a while (n = 1); will amplify negative feelings (n = 1)
**Force too much structure:** Approaching life too analytically (n = 6); negative feelings about technology aspect (n = 3); reducing things of personal importance to a number (n = 1)
**Not be suitable for particular activity or behavior:** Very little or no change so tracking not needed (n = 2); behavior too erratic to be recognizable pattern (n = 2); activity is so routine, no need to track (n = 1); aspect of health is already bad (eg, night vision) (n = 1)
**Be too complicated, error-prone, or disruptive:** Need expert to interpret data (n = 8); data won't be accurate (because, eg, being observed, combined activity of family, algorithm may be biased) (n = 5); too much effort or time required (n = 6); privacy concerns (n = 2); data collection will be disruptive (n= 1)

### Sorting Exercises with Laypeople

Participants frequently expressed surprise at how many constructs they selected to track. On average, participants indicated that they wanted to track more than a third of the constructs, answering “Yes, I want to track” for an average of 28.6 constructs out of 60 during the general sort.

The constructs selected most frequently for both exercises are listed in Table 1 (see Multimedia Appendix 4 for the complete table). While more traditional clinical metrics (blood pressure, heart rate, blood sugar, hormone levels) are represented, there are also many constructs related to the quality of activity (multitasking, variation from routine), mental states (short-term memory, ability to concentrate, laughing), and behaviors (snacking, idle time, bed time).

**Table 1:** Constructs selected by at least 60% of the layperson participants in the general and investigation sorting exercises

**Most Frequently Selected Constructs**			
**General Sort (n = 21)**		**Investigation Sort (n = 20)**	
**Construct**	**Percentage**	**Construct**	**Percentage**
Correspondence with friends and family	81	Time at which you go to sleep	80
Heart rate	76	Ability to concentrate	75
Muscle tone	76	Idle time	75
Short term memory	71	Hormone levels/cycles	70
Pitch perception (hearing)	71	Heart rate	65
Time at which you go to sleep	71	Commitments	60
Use of space	71	Variation from routine	60
Ability to concentrate	67		
Hormone levels/cycles	67		
Laughing	67		
Multitasking	67		
Snacking	67		
Blood pressure	62		
Blood sugar (glucose)	62		
Commitments	62		
Posture	62		
Variation from routine	62		

While sorting, participants sometimes expressed personal reasons for selecting a construct. For example, one participant selected *alcohol drinking* not out of concern for excess, but because he was interested in keeping a record of his wine preferences. Another participant, in selecting *alcohol drinking* mentioned the recent purported health benefits of a glass of red wine per day, perhaps thinking of tracking as a method of recasting a pleasurable activity (drinking wine) as a health-directed activity. A third participant chose *alcohol drinking* expressing concern that her husband was drinking too much, perhaps looking to make him more aware of his own patterns if they tracked consumption as a couple.

The second sorting exercise, the “investigation sort,” offered participants an opportunity to confirm or modify their tracking selections according to a more concrete tracking investigation. Physical activity and stress were the most frequently selected investigations. On average, participants answered “Yes, I want to track” on 20.8 constructs out of 60. Although participants chose fewer constructs on average for an investigation, 18 out of 20 participants added constructs that they had previously categorized as “No, I don't want to track” on the general sorting exercise. On average, participants reassigned 5 constructs from “No” to “Yes” when asked to think about investigating a particular area of their lives. Examples of these constructs include idle time, awareness of time, ability to concentrate, and impulsiveness.

We now describe an example of how re-sorting using the investigation sort exercise inspired a participant to express a desire to track a variable that she would ordinarily avoid.

A semi-retired woman, aged 59, who was working as a part-time chef, first sorted the constructs according to whether she would like to track them over time. She put *idle time* in the “no” category, explaining that she has a tendency to think that she has to “stay busy” and would feel uncomfortable examining times when she is not. After completing the general sort, she was asked to select an aspect of her life that she wanted to learn more about or change. She chose *stress* and sorted again. There were a few constructs that she selected in the general sort, such as *use of space*, that didn't seem to apply for this focused investigation, so she put them in the “no” category. However, there were several that she had previously rejected, including *clothing choices*, *night vision*, *trips to the grocery*, and *idle time*, that she chose for this re-sort. She was surprised that the more focused inquiry altered her choices. She explained why she changed her mind about tracking *idle time*: “[When] I'm in a down mood, idle time really is disturbing to me.” She went on to explain that her reaction to idle time was therefore a useful indicator of degree of stress.

## Discussion

Here we summarize some of our impressions, which we believe would be helpful for designers of health tracking systems. We qualify this discussion with the caveat that cultural differences and differences in medical care between countries could lead to different results in non-US populations using the same data displays.

Our participants envisioned conducting customized, short-term health investigations. People in middle life are often trying to address complex personal health issues, such as regular fitness routines and complex diets; sometimes these goals seem to dominate their lives. They are often dealing with radical changes to their lifestyle, such as divorce and retirement. Most participants wanted to be able to conduct short investigations, either to address a desired behavior change or to identify factors influencing their health and behavior. Typically these investigations would not start from scratch. The person has a theory about a factor that may be influencing how she or he feels and would appreciate tools that help isolate the other factors that influence the condition. Daily rhythms (including variation from routine, sleep/wake cycles, and time spent inside/outside), habitual behaviors (eating, posture), and physical health and activity were of consistent interest. Many people were also interested in mood and social interaction.

Participants frequently expressed an interest in monitoring patterns that would probably not be considered “health”-related by medical professionals. For example, some expressed a desire to be able to examine personal living patterns and variables such as time management, life-work balance, and social relationships, all variables that change as a result of life stage and other factors. These may be related to health indirectly, but they were typically not mentioned by the first set of health professionals as factors that should be tracked.

In direct contrast with concerns expressed by professionals, most laypeople wanted data about their health and behavior even if it invited negative self-evaluation (eg, declines in short-term memory, excessive time spent watching TV). They felt this information would motivate them to change a problematic behavior or help them determine how to get preventive care and compensate for health and performance changes. Another concern expressed by health professionals was evaluation against norms. Most laypeople interviewed were prepared to evaluate data according to their own expectations and goals and without a need for norms or expert evaluation (with some exceptions, such as skin changes).

Concerns about intrusiveness primarily occurred when examining the communication of data between family members. Participants were reluctant to “force involvement” by sharing data about their own activities and health with family members. Two parents with young children and adolescents suggested that there were some data they did not need themselves but that their children would benefit from (eg, time spent on morning routines). Participants suggested that family members may not be willing to share in the tracking investigations or might use data to fuel conflict. It was also evident, however, that participants were interested in leveraging social relationships to address health issues. Privacy issues also emerged when participants considered monitoring their behavior at work.

Constructs that had a duration component (eg, time spent cooking) were considered stressful or overly structured by many people. Data on short time scales were often seen as redundant to memory or provoked feelings of self-consciousness. Some participants found the concept of tracking over years difficult to grasp and struggled to identify variables they would want to track; others expressed that the long time scales might help them gain perspective on lifestyle and re-assess life goals.

Some participants felt that data collection or intervention tools that explicitly interrupt the user may be perceived as reinforcing negative states (eg, having to say you are sad may make you sadder). An exception to this was a tool that might help the user identify and get perspective on rising anger.

Finally, there was a tendency to focus on reprimand—data that tells them what they are doing wrong (eg, “how much time I'm wasting,” “how I'm not calling my family enough”) rather than on how data can help them solve a problem or reinforce positive self-evaluation (eg, “I *am* spending enough time calling my family”). This may reflect a bias ingrained by our medical system. Tests or professional medical advice are most often either ignorable if the news is positive (eg, no cancer detected) or they indicate bad news about a condition or a behavior. Put simply, no news is generally good news. A ubiquitous computing system that may collect and provide data to proactively help improve physical, social, and mental well-being is a concept that most people have not yet considered.

### Effectiveness of Methodology

We developed the mock data display interview material as a response to the challenges of eliciting rich and personal responses to concepts for health monitoring technologies using traditional talk interview techniques. They were developed as a way to help participants respond as though they had experienced the technologies without being focused on the rough implementation details of currently available research prototypes. Because participants were offered concepts to react to and expand upon, rather than being asked to respond from a “blank slate,” assumptions held by the researchers about the range and affordances of health monitoring data were undoubtedly conveyed and therefore impacted participant response. Overall, however, we found the use of the data displays to be helpful for eliciting a variety of health tracking concerns as well as ideas for potential applications of longitudinal health data from both practitioners and laypeople. We did encounter a few challenges. First, drawing out personal responses sometimes took some effort. Many participants initially stated reactions in terms of how it could be useful to people in general rather than to them personally and required prompting to focus on their own needs. Second, sometimes participants stated that a factor applied to them but subsequently expressed indifference about tracking and reflecting upon it. It was easy for participants to respond “Oh yes, I email” or “I don't watch much TV” rather than indicating whether they wanted to track the variable.

Participants frequently said that they were well aware of their behavioral, emotional, and performance patterns. We suspect that they may be overestimating their self-awareness given that retrospective recall tasks for common events is typically very poor [26]. A useful line of research would be to determine how accurately people can retrospectively recall and evaluate some of the factors they believe they are familiar with. For those where perception and reality are different, an interesting question is whether people should be made aware of the discrepancy.

As a group, our participants generated an impressive list of ideas for directed investigations (eg, “What is the level of tension in the house?”, “How close am I to needing to consider back surgery?”). It was therefore notable when participants struggled with a display, sometimes focusing on isolated variables, rather than variable relationships, or overlooking examples of antecedent conditions or contextual factors associated with behaviors (eg, actions and events leading up to skipping exercise). With prompting, they often saw the value in widening a health or behavior-change investigation to consider many more variables in addition to the obvious ones. Their difficulty generalizing from the displays to their own situations may be attributable to limitations in the mock data displays; however, further research is warranted to discover ways to best uncover people's mental models and curiosities about their own behavior that could motivate the design of novel ubiquitous health care applications.

### Recommendations

Ubiquitous computing technology may offer impressive new capabilities for home monitoring to support traditional clinical diagnosis and health assessment. However, due to concerns about cost, privacy invasion, and how end users would react to such monitoring, our interviews suggest that a fruitful direction for researchers interested in home health monitoring to pursue may be to develop technologies that allow for personalized home tracking investigations. Our interviews clearly show that while there is great variability in what factors about their life people would want to track, most people in our interviews did have concepts they would longitudinally track and self-monitor given appropriate technology. Our interviews also suggest that what people wish to track will change over time, based upon their age, life circumstances, interactions with friends and family, health status, and general curiosity. Based upon these interviews, we recommend that ubiquitous “monitoring” systems may be more readily adopted by end users if they are developed as tools for personalized, longitudinal self-investigation that primarily help end users, instead of or in addition to medical professionals, learn about the conditions and variables that impact their social, cognitive, and physical health.

We advocate research on ubiquitous computing longitudinal health monitoring systems that do the following: (1) collect information with as little interaction required from the end user as possible; (2) find appropriate times and places for users to reflect, review, and initiate investigations; (3) help people conduct short-term investigations about issues they are curious about, sometimes “channeling” fun or interesting tracking into deeper, long-term health investigation; (4) build upon the health investigations that people are already engaged in, supporting people as they try diets, train for events, or participate in exercise programs; (5) support nonhealth areas of interest, such as tracking spending, time management, home design, communication with friends and family, and memory support, in order to facilitate collection of health-related data; (6) help people explore relationships between variables that at first they might not consider (eg, mood and TV watching) and appreciate unexpected variability; and (7) help people focus on accomplishments and positive behaviors.

## Appendix

All Multimedia Appendices are also available at: http://architecture.mit.edu/house_n/data/tracking/

### Multimedia Appendix 1

Mock data displays

### Multimedia Appendix 2

Masked interview transcripts

### Multimedia Appendix 3

Complete list of sorting constructs

### Multimedia Appendix 4

Complete table of selected constructs
