# Supplementary material for: To Track or Not to Track: User Reactions to Concepts in Longitudinal Health Monitoring
Source: J Med Internet Res. 2006 Dec 7;8(4):e29. doi: 10.2196/jmir.8.4.e29 (PMC1794006; doi:10.2196/jmir.8.4.e29)
Supplement: Supplementary file 2 [file jmir_v8i4e29_app2.pdf]

Annotation Categories

BENEFIT – BENEFIT OF TRACKING
CONCERN – CONCERN OR "CON" OF TRACKING

POSITIVE REACTION – INTERESTED/WANTS TO DO ONE OF THE PRESENTED EXAMPLES
NEUTRAL REACTION – DOESN'T CARE OR DOESN'T NEED TO DO ONE OF THE PRESENTED EXAMPLES
NEGATIVE REACTION – WOULDN'T WANT TO DO ONE OF THE PRESENTED EXAMPLES

IDEA VARIABLE – IDEA FOR SOMETHING TO TRACK
IDEA RELATIONSHIP – IDEA FOR HOW VARIABLES MIGHT BE RELATED
IDEA USE – IDEA FOR HOW TRACKED INFORMATION COULD BE USED OR IDEA FOR HOW TRACKING SHOULD BE DONE

PROCEDURE – SOMETHING RELATED TO HOW THE INTERVIEW WAS DONE
CURRENTLY TRACKING – PARTICIPANT IS ALREADY TRACKING AND RECORDING
CURRENTLY MONITORING – PARTICIPANT IS ALREADY MONITORING VALUES (BUT NOT RECORDING)

Interview Notes and Transcription

ID: [L1]

Date: 6/18/04, 1:11-2:15PM (64 minutes)
Participant type: Participant, through poster
Demographics: Man, 40s
Location: House_n
Audio file: DM100023

- Shown 5 Displays: Body, Activity Diary, Snoop, Daily Rhythm, Family

GENERAL REACTION: STRONGLY POSITIVE, ALREADY A TRACKER, WANTS TO TRACK MORE(+) :[L1]

- Already manually tracking a number of health and activity variables (already a serious tracker)
- Uses tracking to motivate behavior change
- Trains for triathlon
- Would like to use tracking data with family

[What I monitor] (0:00:00)

The participant completes detailed records of aspects of his life and was intrigued by the premise of the project.

BENEFIT: MAKING A BEHAVIOR A HABIT BY RECORDING IT :[L1]
BENEFIT: ASSOCIATING A BEHAVIOR WITH HOW IT MAKES YOU FEEL AFTERWARD (E.G., GUILTY) :[L1]
CURRENTLY TRACKING: NUMBER OF ALCOHOL DRINKS :[L1]
CURRENTLY TRACKING: EATING FAT (DONUTS) :[L1]

"If I just try to make these things a habit, I'll be healthier, generally, and the only way I can do that is if I have to write something down and track it. If I go back and say, what did I do last month, and I say well, there was this one day I went out to a bar and had 6 drinks, I feel guilty, and I say, if I track it and I have to write down 6 drinks, then I'm not going to do that any more. Or if I sleep late, or if I, I always feel bad after, so if I have to keep a journal of that, then I'll be less apt to corner myself and do something on a whim. Same thing with eating donuts or eating fat. I track how much fat I eat and how much exercise, I want to exercise at least an hour a day." :[L1]

The participant shows me his tracking records, in a daytimer book.

CURRENTLY TRACKING: TIME WAKING UP AND GOING TO SLEEP :[L1]
CURRENTLY TRACKING: TOTAL HOURS OF SLEEP :[L1]
CURRENTLY TRACKING: HOURS OF WORK :[L1]
CURRENTLY TRACKING: SPORTS AND TRAINING :[L1]
CURRENTLY TRACKING: MEDITATION :[L1]
CURRENTLY TRACKING: BREAKS FROM TRAINING :[L1]
CURRENTLY TRACKING: HOW YOU FEEL ABOUT YOURSELF :[L1]
CURRENTLY TRACKING: SEX, FREQUENCY AND SATISIFACTION (REVEALED LATER) :[L1]

"I've been through several different ways to do this. This is just monthly data tracking I've done over the years. It says what time I went to bed and what time I get up, total hours of sleep, total hours of work, caffeine, alcohol, swimming, biking, running, lifting, calisthenics, mediation, yoga, and some other, I don't know, other personal things I have in here that kind of affect my life that I want to keep track of. I keep it all so it's very visible so I can track it and see exactly what I've done, what races I've done, when I've taken a vacation, and how good I feel about myself. So if I do that, it helps me move forward and not do the things I don't want to do." :[L1]

CURRENTLY TRACKING: WATER DRINKING :[L1]

CURRENTLY TRACKING: FOODS, VEGETABLES :[L1]
CURRENTLY TRACKING: VITAMINS :[L1]

"How much water I drink, foods, vegetables, supplements, actual meals that I have had, I keep track of everything I've had, then exercise on the other side." :[L1]

IDEA RELATIONSHIP: DISTANCE, PACE, HEARTRATE :[L1]
IDEA RELATIONSHIP: DRINKING AND HOLIDAYS/SOCIAL OCCASIONS :[L1]

He reviews his records weekly and monthly. He uploads the data into a spreadsheet, but it is difficult to organize and graph. He looks at mileage that he's run throughout a month, pace, and heartrate. He also tracks how many alcohol drinks he has for the month and notes that around Christmas time, when going to a lot of parties, this value can really add up. "I'll say okay, I've had too many drinks for this month."

IDEA RELATIONSHIP: WEIGHT AND LIFTING :[L1]
IDEA RELATIONSHIP: ALCOHOL CONSUMPTION AND PACE :[L1]
IDEA USE: TRIGGERING A TEMPORARY ADJUSTMENT TO COMPENSATE FOR UNHEALTHY BEHAVIOR :[L1]

He looks at how his weight is affected by how much lifting he does and how that, along with aspects such as alcohol consumption, affects his pace. He does not record his conclusions, but he may make behavioral changes, such as getting up earlier, running more, give up coffee, drink less when he and his co-workers go to a bar after work, or "not eat donuts when there are free donuts day at the office." He makes an adjustment for a period of time, then goes back to the habit until an event triggers a change again.

"I like all those things, I like donuts, these are types of things that I enjoy and if I stay off of them for that long, I get burned out." :[L1]

"I only lift for four months out of the year, because getting up at 5am to lift is hard. So I say, okay, I need a break, so I'll give myself a little mini-vacation. It would be nice to make it a habit, keep off all this stuff and exercise 2 hours a day, I would love that, but there's just too many things going in my life." :[L1]

"These are the things that are most important to me, exercise and health, and being happy. If I don't make that a number one priority, it's very easy not to exercise. It's very easy to stop at McDonald's and have a hamburger. All these things in life makes it so easy to do the bad things, that you really have to force yourself to avoid them. The only way I can avoid them is to record, okay you were bad, or you were good." :[L1]

"He's been tracking for 10 years. He tries to keep up a four-week cycle for running, biking, and swimming to train for Iron Man races. He notes that being at the wrong part of the cycle at the race can be detrimental, so he carefully structures his training according to a schedule and does more tracking with a spreadsheet during that period (miles per week, pace, type of run). He notes though that there is so much data from the other areas of his life, he does not include that in his charting analysis. The times when tracking matters the most, for his training, he has the least time to track. He would like to see, for example, whether meditation and stretching, affects his race and affects how he feels, but hasn't had time to do that analysis." :[L1]

BENEFIT: FINDING AN OPTIMAL STATE :[L1]

His goal is to "be optimal," and to feel good, regardless of performance.

IDEA USE: COMBINED MUSIC, HEART-RATE, GPS FOR ATHLETES :[L1]

He has a heart rate monitor and would like a pace tracker, but has difficulty carrying all his devices. He hopes that there will be a combined MP3, heart rate, GPS device available in the future.

He has a PDA and has tried doing the tracking on it, but has not found software that works well for both recording and graphing.

[Body] (0:11:15)

POSITIVE REACTION: SKIN RECORD :[L1]
POSITIVE REACTION: HEALING TIME :[L1]
IDEA VARIABLE: HEALING FROM SUNBURN :[L1]
IDEA VARIABLE: NUMBER OF COLDS, ALLERGIC REACTIONS :[L1]
IDEA VARIABLE: PSA :[L1]

"I do this [keep a skin record], actually, I had spots on my back from a bad sunburn. Every time I'd go to a doctor, they would say, oh they're just spots. I wanted to track them over time to see if they are changing. I keep track of whenever I'm sick or have a cold. When I start feeling bad and how long I'm feeling bad. Not things like papercut, although that would be – if you keep track of the little things, and something takes a long time to heal, something's wrong. Some of the yearly things I keep track of are cholesterol, PSA, different types of blood levels. So when I go to my annual physical, I have a medical folder, and they ask me how I've been, I can say I have had two colds, allergies, I had this. My PSA last year was this, and my cholesterol was this, my blood pressure was this, so when they say it, I can know what it is in relation to what I've been year after year." :[L1]

BENEFIT: EVIDENCE FOR CONVERSATION WITH DOCTOR :[L1]

He does not share his records directly, but he says he would share photographs of skin change to have "proof" so the doctor would take him seriously.

He was surprised to see these examples and said he thought he was alone in tracking.

NEUTRAL REACTION: MUSCLE TONE – MEASURED IN ANOTHER FORM (EXERCISE COMPLETED) :[L1]
POSITIVE REACTION: EXERCISE RECORD :[L1]
POSITIVE REACTION: ACTIVITY RECORD :[L1]

He was curious about how muscle tone tracking would be done, but stated that he would not be interested in tracking it, as it follows from the exercise he does. He already records exercise record and activity record.

POSITIVE REACTION: ACHES AND PAINS :[L1]
IDEA RELATIONSHIP: FREQUENCY, HEADACHE, DURATION :[L1]
IDEA USE: REMINDING YOU TO CHECK TO SEE HOW YOUR BODY IS AND WHETHER CONDITION HAS STOPPED :[L1]

"Aches and pains would help. You say, oh I get a lot of headaches, but you really don't track that. I typically don't have a lot of headaches and I try to keep off medication, but if I was to take aspirin or something, I'd want to track that. How long I had, I occasionally have headaches that last two days, and I don't really know when they end, because I don't notice when they end, but if I tracked it – if it was a quick menu, then it'd be like, oh I don't have the headache. It's like a tickler to remind you to check to see how your body is." :[L1]

[Daily Rhythm] (0:17:30)

IDEA VARIABLE: TIME SPENT WITH FAMILY :[L1]
BENEFIT: DISCOVERY THAT COMMITING SUFFICIENT TIME ON SOMETHING (E.G., SPENDING TIME WITH FAMILY) :[L1]
BENEFIT: GIVING LEAVE TO GO OUT OF BALANCE, IF HAVE MADE UP FOR IN THE PAST (I.E. HAVE SPENT TIME WITH FAMILY EARLIER IN THE WEEK, CAN NOW FOCUS ON WORK) :[L1]

He's already tracking the time he is waking up and going to sleep. He noted the importance of seeing how much time you spend with your family.

"Okay, if I'm on a big project and I don't spend a lot of time with my family, should I really feel guilty, oh no, I spent 8 hours with them on Friday. That's good." :[L1]

POSITIVE REACTION: FIRST CONVERSATION OF THE DAY :[L1]

He thought first conversation of the day was interesting and joked that you could be alone in the office all day [as I was at the time of the interview] and feel depressed, and then realize it was because you hadn't spoken with anyone.

CONCERN: DEPRESSING TO SEE MAGNITUDE OF BEHAVIOR (POSTPONED COMMITMENTS) :[L1]

"That would be depressing, how many postpones I have. I do track things like that with a Franklin planner. You have the things where you extend it out. I am a list person. I have 25 critical things I have to do before my mother comes, before next week's camping trip - I just cross them off and keep adding to them." :[L1]

IDEA USE: VISUAL TO-DO LIST :[L1]

CONCERN: ONLY SO MUCH CONTROL OVER LIFE, FAMILY MEMBERS HELP DETERMINE HOW THINGS ARE DONE :[L1]

He shows me one of his to-do lists that he prints off every day with top priorities, "very soon," "whenever." In free moments, he looks at his list and plans what he needs to do next, such as stopping off at the store on the way home. He feels like it could be better organized if made visual (as in the example). He puts the list together each evening and finds that he can alleviate anxiety by going through the list and deciding what pieces of it he can do the next page. He would like to have a master list from which he could drag items to the week and then look for chunks of time when other items can be introduced. He's not sure about the place-based aspect of the commitments and would have to see it in action, as applied to his life.

He notes that being in a relationship introduces new elements that affect his ability to follow through with a list.

[Activity Diary] (0:25:00)

POSITIVE REACTION: TV :[L1]

"TV is good. I get addicted to TV and feel guilty. I guess this whole thing about me is feeling like I've wasted time. Time is too important to waste on TV, but guess what, the playoffs are on. I don't schedule it, and then I feel guilty if I watch it. I feel like I'm sneaking." :[L1]

IDEA VARIABLE: WHAT ACTIVITIES MAKE YOU HAPPY :[L1]

IDEA VARIABLE: WHAT ACTIVITIES MAKE YOU DISTRESSED :[L1]

He's read the book Flow and likes the idea of understanding fluctuations in mood. He would like to know in the past, what made him happy. He gave examples of participating in a playgroup with kids which may have brought happiness in the past but is no longer part of one's routine and meeting with the boss, which may cause distress and indicate that a new job should be sought.

INVESTIGATION: DOWN DAYS :[L1]

"Some days I'm just very depressed and don't know why." :[L1]

NEUTRAL REACTION: REFRIGERATOR OPENINGS – NOT A DIETER :[L1]

He wasn't interested in refrigerator openings. He likes to track what he eats, but is not a dieter.

IDEA VARIABLE: DIFFICULTLY WAKING UP AND STARTING DAY :[L1]

He can imagine going back to look at particular days.

"My big thing is sleep cycles, where I can get very little sleep and feel like I get a million things done and other times when I can't wake-up until the last minute before I have to leave. If I can look over time and say, okay, here it comes, it's September and October and I'm feeling like I can't get out of bed for some reason, either I'll start

accepting that or planning a 7am swim and commit with a friend to do that, so I don't have to feel bad. If I get the mood rating very low because I'm not accomplishing what I want to accomplish because I'm sleeping too late, then I'll swim with a friend. I'm guaranteed to wake-up whenever I plan something with someone first thing in the morning. So this would definitely be helpful and I try to do that, but it's hard to track that." :[L1]

[Snoop] (0:28:15)

POSITIVE REACTION: EATING BEHAVIORS :[L1]
POSITIVE REACTION: POSTURE :[L1]
POSITIVE REACTION: OPPORTUNITIES FOR EXERCISE :[L1]
POSITIVE REACTION: WORK HABITS :[L1]
NEGATIVE REACTION: SOCIAL AWARENESS :[L1]
IDEA RELATIONSHIP: POSTURE AND BLOOD-FLOW AND TIREDNESS :[L1]

He immediately liked the idea of having information about eating behavior, posture, opportunities for exercise, and work habits. He was less comfortable with the social awareness. He believes something like posture can affect blood-flow and tiredness. He noted that an outside viewpoint would be helpful because it is easy to get into habits you wish to avoid.

IDEA VARIABLE: ANGER, OUTBURSTS/YELLING :[L1]
IDEA RELATIONSHIP: DRIVING AND TEMPER OUTBURSTS :[L1]
IDEA RELATIONSHIP: (NOT) LOSING TEMPER AND MEDITATION/PRAYER :[L1]

"I'm happy with who I am in that aspect. I don't know. I just don't think I could change that at this point in my life. <pauses> I guess I could. I guess sometimes I get mad at someone or something, like driving, you get mad at someone and scream or something and wish that you wouldn't get upset because drivers are always going to be like that. So later on, if I'm at church on Sunday and I'm like how come I felt bad those five times or how come I yelled at that person, because they didn't do a good job, I feel guilty afterwards or feel like I could have done better." :[L1]

"I can control myself if I tell myself, okay, don't do this. If I had to write it down, that I yelled at four drivers or was upset because someone missed a deadline that I was depending on and I got so mad at that person I just felt bad. If I had to track that, maybe I would put myself in their shoes before – why am I so uptight? Maybe I should just relax a little bit. I know when I meditate or pray a lot, I don't do it as much, I'm much more casual and much more relaxed, during any circumstance. I can reframe it." :[L1]

The participant therefore concluded that he would then like to track that aspect of social awareness (feelings of anger or losing temper).

[Family] (0:32:55)

The participant noted that the whole morning schedule and at bedtime should be "red" (running later than expected).

POSITIVE REACTION: MORNING SCHEDULE :[L1]
IDEA RELATIONSHIP: SCHEDULE AND TASKS AND RESPONSIBILITIES BY FAMILY MEMBER :[L1]
BENEFIT: GETTING CONTROL OVER SCHEDULE :[L1]

"If you could set tasks and responsibilities and combine those two things with this [the morning schedule]. Like if mom was in charge of making lunches for the two kids and dad was in charge of getting them dressed, and it was a problem, okay, make lunches the night before, could alleviate the problem. Mornings are never smooth, but maybe that's because they are not controlled as they should be. That would be helpful." :[L1]

POSITIVE REACTION: PERCEPTION OF HOW LONG SOMETHING TAKES AND HOW LONG ACTUALLY TAKES :[L1]

BENEFIT: COMMON MATERIAL FOR FAMILY DISCUSSION :[L1]

The participant agrees it would be helpful to distinguish between the perception of how long things take and how long they actually take and notes that it would help when discussing things with another family member.

NEUTRAL REACTION: WATER USAGE :[L1]

The participant had no interest in the water saving example, but liked the eating together example.

POSITIVE REACTION: EATING TOGETHER :[L1]

"Very good. I like that. I know that the more time you spend eating together at the table, studies have shown you are a better adjusted family." :[L1]

The participant thinks that his family would "definitely" be comfortable talking about these issues as a family and family members would not get defensive.

POSITIVE REACTION: READING, NEW WORDS :[L1]

"I like the part of tracking reading. I'm not sure about the shared themes. New words would be a good topic of conversation, if everyone tracked new words and related it to everyone else at the end of the day, oh these are the words we learned. But we all read at different times, so I don't know when the new words thing would come up – maybe it's by the week and not a daily thing." :[L1]

POSITIVE REACTION: CORRESPODENCE :[L1]

"I need this <tracking extended correspondence> for potential, in the future correspondence with family, I need to call them at least once a week and on their birthday, so I need some kind of tickler." :[L1]

POSITIVE REACTION: GROUP ACTIVITY :[L1]

CONCERN: MAY BE DIFFICULT TO TRACK DIVERSE ACTIVITIES :[L1]

"We basically try to do, once a week, we come home from church, and everyone goes out running together. Then it's split up by each kid going and playing softball or swimming or something. Very tough to track. I have four kids so it's very difficult. It's hard, but it could be done." :[L1]

IDEA USE: SETTING GOALS TO DO THINGS TOGETHER :[L1]

The participant feels that it is important to try to do activities together.

"If you don't have a goal to do something, to be together, then the whole weekend can go by and you can miss everything." :[L1]

[Sorting] (0:41:00)

Resorts by physical activity, mood, family relationships

ID: [L2]

Date: 8/30/04, 4:30 – 5:30 (55 minutes)
Participant type: Mailing list
Demographics: Man, age 40
Location: Phone (California)
Audio file: DM1000

- Shown 5 Displays: Snoop, Diary, Performance, Social, Daily Rhythm
- Is professionally and personally interested in archiving
- Blogs
- Would like to have a better sense of what he's read/watched/heard and where – a google engine for the media he experiences
- Would like to see projected consequences for actions (if you lower the thermostat and keep it low… if you eat that snack now…)
- Would like context and interpretation, a system that would help tease out destructive routines
- There are many things he doesn't feel a need to track now, but he would like to take 6 weeks and get a baseline for a variety of health and activity variables so they can be compared against when he gets older

Work: Futurist, archivist, blogger; married with two young children
Technologies: cell phone, digital camera, PDA, laptop
Monitoring devices: scale, no pedometer, used to have a heart rate monitor

[What I monitor] (0:05:29)

CURRENTLY TRACKS: WORK PROGRESS (BLOG) :[L2]

He has had work-related journals since college and thinks of them as "extensions of my memory." The blog serves somewhat the same purpose. He keeps emails as an archive.

CURRENTLY TRACKS: WEIGHT :[L2]

He monitors his weight.

He keeps track of when his car needs oil-changes.

CURRENTLY TRACKS: CHILDREN'S MEDICAL VISITS :[L2]

He monitors the health of his children, keeping track of their medical visits. He is not monitoring his parents' medical visits yet, but expects to do so in the future. He informally monitors his parents by communicating with them through email. One of his parents travels quite a bit, so email is more convenient.

He has a blog about his kids that his family reads on a regular basis.

BENEFIT: MAKE PUBLIC PROCESS AS WELL AS RESULT (BLOGS) :[L2]

He treats his web blog like a research notebook. He previously worked on electronic archiving projects. He likes the idea that blogs "make public materials that would normally stay in the background in the research process."

CURRENTLY TRACKS: CHILDREN'S MILESTONES AND DAILY ACTIVITIES :[L2]
BENEFIT: SHARING CHILDREN'S GROWING UP WITH FAMILY :[L2]

Benefit: intellectually stimulating when like ethnographic fieldwork (kid blog) :[L2]
He thinks of the blog he keeps for his children as a variation of the baby book. He notes that keeping a baby book is almost culturally mandated for parents. He thinks of the traditional baby book as a place where one records milestones and a way of remembering how one's children were even when they have changed significantly (e.g., pierced teenagers who don't talk to you). He found he never wrote in it himself. Once he had the public medium of a blog, he was motivated to begin writing about his children. If he doesn't update it weekly, he may receive a message from his mom or relatives asking for updates. He notes that the traditional baby blog is about "first things," whereas he uses his kids' blog to write more about the daily things that they do. He thinks of it as "ethnographic fieldwork" and finds it more intellectually stimulating.

[Snoop](0:15:35)
Positive reaction: snoop :[L2]
Looking at eating,

```
 I suppose there would be some utility to having external analysis of these sorts of
  patterns. :[L2]
```

Positive reaction: posture :[L2]
Idea use: long term consequences of particular postures :[L2]
Idea investigation: posture in different contexts, as mapped to social cues :[L2]
Concern: whether technology can give sufficient information about social implications of behavior :[L2]
He never worries about posture, but would think it would be helpful to have information about whether there might be long-term consequences to sitting one way or another. He would also like information about how posture he exhibits in different contexts serves as a social cue and whether that is to his benefit. He is skeptical that a machine would be able to do that.

He notes that the exercise examples are more prescriptive than the posture and eating.

Positive reaction: social awareness :[L2]
He thinks social awareness would be relevant for many people. He was interested in what the system would need to know to accomplish some of the examples.

Positive reaction: eating behavior :[L2]
Positive reaction: exercise :[L2]
He thought that the eating and exercise were the most useful, but none of them were "stupid" either.

Positive reaction: work habits :[L2]
Concern: work habits are too idiosyncratic for system to provide useful feedback :[L2]
He thought about the work habits examples in terms of "coaching." He noted that work habits can be so idiosyncratic, particularly with regard to thresholds for distraction, etc., that developing a system smart enough to give this type of advice would be challenging, but it would be beneficial.

Benefit: reinforcing existing monitoring, which we currently don't do a good job at (eating behavior) :[L2]
I asked him if he could choose one that he would personally want to focus on over the next few weeks and he chose "eating behaviors" because it is the most familiar kind of monitoring and we don't necessarily do a good job at it. He believes that he basically knows what to do (eat less, make better choices), but reinforcement is always a good thing. Work habits would be the second choice for him.

IDEA USE: FEEDBACK ABOUT BEHAVIORS AT TIME WHEN CAN CORRECT :[L2]
He believes that if the information were delivered fast enough so that he could correct his behavior, these examples of feedback would not make him feel negatively self-conscious.

He noted though that even having comparisons with other people on things like posture would not be "particularly devastating or anything"

[Diary](0:22:54)
IDEA VARIABLE: REFRIGERATOR OPENINGS FOR SELF VERSUS FAMILY :[L2]
He wondered whether the refrigerator open data was just him or was for the household. He imagined that you might want both views.

NEUTRAL REACTION: TV WATCHING, NOT NEEDED UNTIL RETIRED :[L2]
IDEA USE: WHEN SEDENTARY BEHAVIOR DETECTED, REMINDER ABOUT BEING ACTIVE :[L2]
He thought he would be more concerned about TV watching if he were retired and watched more TV. He knows that sedentary behaviors are important to monitor and thought it would be helpful to be have a reminder during those time that you could be doing something more active instead.

IDEA VARIABLE: WHERE ONE DOES WORK USING GPS :[L2]
He's never experimented with mood ratings before. He wanted to track "where he works" using something like GPS on a cell phone. He favored this approach over self-report.

IDEA USE: TRACING REFERENCES FROM MATERIAL READ :[L2]
He reads a lot of material, including scanning of papers and web sites, and would like to have tools that would help him relocate references when they later become more relevant. He would like a system that would help him retrace his steps in terms of what he was reading and where he saw information.

BENEFIT: ANTI-LIABILITY DOCUMENTATION :[L2]
He thought there might be anti-liability information that would be useful related to keeping an activity diary.

BENEFIT: IDENTIFYING SUCCESSFUL PATTERNS (BILL-PAYING UNDER BUDGET) :[L2]
IDEA RELATIONSHIP: BILLS PAID, WHEN PAID, ABILITY TO STAY WITHIN BUDGET FOR MONTH :[L2]
IDEA USE: BILL-TRACKING SYSTEM WITH REMINDERS :[L2]
He liked a system that would track that you've paid the bills – a system that provides reminders, particularly for bills and financial information (school payments, mortgage), along with reassurance that expected transactions have been completed. He noted that the pieces of information are easy to keep, but hard to integrate, and hard to associate with reminders. He mentioned that he would like to think about how to stay within a budget (perhaps recording when payments are made and how during months when successful at staying within budget).

BENEFIT: UNDERSTANDING CONSEQUENCES OF ACTIONS :[L2]
IDEA USE: CONSEQUENCES OF ACTIONS (E.G., DIET, THERMOSTAT) :[L2]
He talked then about how he wanted a system that would help him understand the consequences of actions. He notes that on a diet, one problem he has had, is that he will lose some weight, reach a goal, and then start to slough off a bit and gain it back.

It would be useful for me, if something could tell me, okay, you've lost two pounds, but if you do this course of behavior, you'll continue to lose, if you don't, you're just going to gain it back. It would be

useful to have that when I'm ordering in a recipe. If you get the broiled chicken, if you eat this way, your diet…

[0:32:00 phone system interruption until 0:35:22)

IDEA VARIABLE: PATTERN OF CALLS TO PARENTS :[L2]
BENEFIT: IDENTIFYING DISRUPTIONS TO PATTERNS :[L2]
IDEA USE: IDENTIFYING PATTERNS THAT CAN BE DISRUPTED (E.G, BREAKING PATTERN TO WEEKLY CALLS WITH PARENTS, RESULT IN LONG LAPSE BETWEEN CALLS) :[L2]
Something that would be able to identify particular patterns that I engage in – eating and exercise our good examples. I suppose it might also be doing things like, not calling your parents. If you don't do that – if you usually call them every weekend, but if you miss one, it might be 6 months before you do it again. .. being more cognizant of those patterns would be a very useful thing.

IDEA USE: SYSTEM THAT PUTS DATA IN CONTEXT, PARTICULARLY FOR CONSEQUENCES OF ACTIONS AND ALTERNATIVES :[L2]
For some things, he thinks he could identify the patterns himself. His preference would be for systems that can also give some context, a system that would be provide some interpretation would be useful in most categories.

> Let's say you are thinking of going on a trip… if you do this, it's going to cost this much money, and it's going to have this impact on your household finances. So maybe going on a hike would be a better thing, and it would be better for your health. :[L2]

CONCERN: INTERPRETING MEDICAL VALUES, SUCH AS LIPIDS :[L2]
He noted that interpretation is particularly important for health data, because he often can't remember is – high lipids good or bad?

> Once you get past stuff like heart rate, blood pressure, and weight, I get into a category where I want something that can help me understand what this stuff means. :[L2]

[Performance](0:38:45) – participant choice
He commented that memory would be a "downward slope" for him.

NEUTRAL REACTION: GRIP STRENGTH, STRENGTH TRAINING, MEMORY, AND ATTENTION, MORE IMPORTANT IN 20 YEARS :[L2]
He thought grip strength, strength training, memory, and attention would be more important to him in 20 years.

IDEA USE: HELPING TO ACHIEVE PEAK CONDITION :[L2]
> At my current age, I am getting concerned about getting closer to peak condition, wherever that might be. As you get older, I think you get more concerned with, where is the peak and is the peak eroding. :[L2]

He noted that you can take for granted constructs like memory and attention.

IDEA USE: IMPROVEMENT IN GAME PERFORMANCE AS INDICATOR THAT PLAYING TOO MUCH :[L2]
He thought tetris performance might be a reminder not to play so much of it. He notes that games on cell phones get a lot of play time during waiting times.

[Social](0:41:35) – participant choice

POSITIVE REACTION: GOOD TIMES AND BAD TIMES TO CALL :[L2]
CONCERN: HISTORICAL DATA CAN BECOME ERRONEOUS WHEN MAJOR LIFE CHANGE OCCURS (MOVING, HAVING BABY) :[L2]
He liked the good time to call and bad time to call example, particularly if the system could coordinate information from the recipient of the call. He noted though that significant changes, like someone having a baby or moving to a different time zone, could make historical information incorrect.

[Daily Rhythm](0:42:45)
BENEFIT: DAILY RHYTHM FOR IDENTIFICATION OF SIGNIFICANT CHANGES :[L2]
POSITIVE REACTION: DAILY RHYTHM FOR PARENTS, SELF WHEN OLDER :[L2]
CONCERN: TOO MUCH DETAIL FOR RIGHT NOW (DAILY RHYTHM) :[L2]

This is definitely the kind of stuff that I would want to be able to – it's basic enough – I don't know if I want this level of information, but certainly as I get older and my parents get older, something that would alert me to significant changes would be a useful thing. :[L2]

POSITIVE REACTION: DAILY RHYTHMS FOR CHILDREN :[L2]
He could imagine wanting to look at the routines for children.

[Family](0:43:45)
POSITIVE REACTION: WATER USAGE, CONSEQUENCES OF ACTIONS :[L2]
He liked the water usage example because it's consistent with his "consequences of your actions" idea.

IDEA RELATIONSHIP: ADJUSTMENTS TO THERMOSTAT AND SAVINGS :[L2]
For me, being able to see if I turn down my thermostat to 60 and I leave it there *all winter*, this is how much money we'll save by the spring. I would find that kind of thing quite useful.

POSITIVE REACTION: EATING AT TABLE :[L2]
IDEA USE: DATA IN CONTEXT OF STUDIES (EATING AT THE TABLE) :[L2]
IDEA USE: DATA IN CONTEXT OF HIGHER LEVEL GOALS (HEALTH OF FAMILY -> EATING AT THE TABLE) :[L2]
Because his children are young (2 and 5), there is little variation in their dining routines, but he could imagine that when they are older, eating at the table might be relevant. He wanted, however, there to be context with the data. For example, do studies suggest that eating together affects the health of the family relationships – how does the amount of time spent doing that stack up against values in those studies. He also imagined that if you had set as a goal or concern the health of the family relationships that you would then want to look at this data.

POSITIVE REACTION: CORRESPONDENCE WITH FAMILY :[L2]
He thought the correspondence with family would be useful.

[Sorting] (0:48:40)
Ability to concentrate was relevant because his work involves the ability to recall information.

CONCERN: IF ALREADY HAVE TOOL TO ACT AS ABILITY, NO NEED TO TEST ABILITY (AWARENESS OF TIME) :[L2]
Because he always has his watch, awareness of time doesn't seem relevant

BENEFIT: HEALTH METRICS WHEN MONITORING CHRONIC DISEASE :[L2]
BENEFIT: HAVING BASELINE DATA :[L2]

If he had reason to worry, he would want information about blood pressure, blood glucose, etc., but not otherwise. When I asked him about having comparison data, he agreed that having baseline data would be helpful

He was interested in tracking headaches because he felt headache patterns may be indicators of stroke; as someone who is involved in a cognitively challenging job, anything related to the brain is of concern for him.

CONCERN: SKEPTICISM ABOUT WHETHER CONSTRUCT CAN BE QUANTIFIED (KNOWLEDGE ABOUT FAMILY) :[L2]
He questioned whether knowledge about friends and family could be quantitatively evaluated, given that there much of it is tacit; when framed as knowledge of birthdates, schedules, and other types of facts, he liked the idea of tracking it.

CONCERN: IF DON'T NEED ABILITY, DON'T NEED TO TRACK (NIGHT VISION) :[L2]
He didn't feel a need to track night vision because he doesn't do much at night without light.

He felt that time at which you go to sleep would be "definitely useful as historical data"

IDEA VARIABLE: BACKGROUND VERSUS ACTIVE TV WATCHING :[L2]
He noted that you could measure background TV watching versus active and would want that breakdown.

He thought there would be many he would say yes to in 20 years (such as hearing).

IDEA USE: 6 WEEK BASELINE INVESTIGATION :[L2]

```
There would be an argument for doing all of this stuff for, 6 weeks, let's say, and then
  turning off basically all the things I say no to, but having all that data for 10 years
  from now (for comparison). :[L2]
```

Resorts by physical activity.

IDEA RELATIONSHIP: COMMITMENTS AND PHYSICAL EXERCISE (MORE TAKE ON, LESS TIME TO EXERCISE) :[L2]
He noted that commitments has an orthogonal relationship to physical exercise (the more take on, less time to exercise)

The main exercise he does is bicycling, so community traffic congestion is relevant.

IDEA USE: GOAL DIRECTED INVESTIGATIONS + BACKGROUND :[L2]
He could imagine being more interested in particular goals – he wouldn't want to turn off the sensors for other things though.

ID: [L3]

Date: 8/25/04, 4:00 – 6:00 (65 minutes)
Participant type: Word of mouth
Demographics: Man, age 40
Location: Phone (California)
Audio file: DM100023_1

- Shown 4 Displays: Journal, Snoop, Place, Social
- Has elaborate eating requirements that require advance planning
- Interested in using tracking to help him focus on his own goals (tends to put others first)
- Concerns about disruptive data collection

Work: High school teacher (history), writer
Technologies: Cell phone, digital camera, PDA (doesn't work right now)
Devices: been thinking about getting scale/pedometer

[What I monitor] (0:02:50)

CURRENTLY TRACKING: MATERIAL FOR CREATIVE WRITING (JOURNAL) :[L3]
He keeps a journal with reflections on his day and material for his writing. He's been keeping it for 20 years.

CURRENTLY MONITORING: CALORIES, CARBOHYDRATES, PROTEIN :[L3]
He's been trying to balance caloric, carbohydrate, and protein intake. He has a chart that he uses, but doesn't record anything.

He notes that his teaching schedule inhibits detailed tracking.

IDEA USE: BECOMING SELF-AWARE AND OBSERVANT (MEDITATION) :[L3]
He's done meditation and believes that this makes him more observant of himself and interested in the best use of time.

[Journal](0:05:40)

POSITIVE REACTION: PEOPLE ENCOUNTERED, MATERIAL FOR WRITING :[L3]
POSITIVE REACTION: PLACES VISITED, MATERIAL FOR WRITING :[L3]
Because he's a writer, he finds much of this material as relevant for inspiring his writing (e.g., people he encounters may become characters). He also looks for material that is relevant for his teaching.

POSITIVE REACTION: SNACKING, FREQUENCY NUTRITION :[L3]
He's trying to work on snacking. He has a high metabolism and snacks a lot; he knows this is difficult to mentally keep track of, so he would like a record of it. He would like to work on making his snacks more nutritious.

NEUTRAL REACTION: WATER DRINKING, ALREADY MASTERED :[L3]
He already drinks plenty of water a day (at least 8 glasses), so doesn't feel he needs to monitor that.

POSITIVE REACTION: MEMORABLE MOMENTS :[L3]
He would definitely record memorable moments.

POSITIVE REACTION: MEDIA STORIES :[L3]

He notes that he already keeps a separate informational notebook that contains references to media that he identifies as relevant for his own interests and teaching. He does not date it and does not relate it to his entries in his personal reflections journal.

IDEA USE: PERSONAL REFLECTIONS AND MEDIA REFERENCES MAY CROSS IF SOMETHING HAS AN EMOTIONAL IMPACT :[L3]

> "Occasionally I'll write about a film that has a real impact on me, but usually those references to media are kept separately. Unless I use them and they become part of a story." :[L3]

POSITIVE REACTION: TRACKING INTERACTION WITH FRIENDS OVER TIME :[L3]

I asked him about looking for trends in this kind of information related to personal development. He agreed that looking at information about interactions with friends over time would be something he would review (and currently may pay attention to with his own journal).

[Snoop](0:10:30)

POSITIVE REACTION: EATING BEHAVIOR, HIGH METABOLISM, HEALTH-CONSCIOUS, STRICT DIET :[L3]

The eating behaviors category was of interest to him. He doesn't feel he is overweight, but he ends up eating a lot because he has a high metabolism. He is health-conscious and does not eat many processed or refined foods – no sugar, caffeine, and very little alcohol. He prepares his own meals and snacks.

POSITIVE REACTION: POSTURE, DUE TO OLD INJURIES :[L3]

He believes he could use some help with posture, due to an old injury in his back, but believes he is in pretty good health.

NEUTRAL REACTION: OPPORTUNITIES FOR EXERCISE, ALREADY COMPENSATES FOR SEDENTARY BEHAVIOR BY SETTING ASIDE TIME EACH WEEK FOR VIGOROUS EXERCISE :[L3]

He spends a lot of time sedentary when he is writing, so he makes an effort to really get out and go hiking.

IDEA VARIABLE: WAITING TOO LONG TO EAT :[L3]
IDEA VARIABLE: REGULARITY OF VIGOROUS EXERCISE :[L3]

I asked him to imagine what they might discover about him of which he may not have a good awareness. He thought they may discover that he sometimes waits too long to eat. He noted that it may be tough to recognize because he appears that he has a lot of energy and endurance even when he is needing to eat. They may notice that he slouches too much. They may discover that he doesn't get enough exercise, as sometimes he goes two weeks before getting vigorous exercise.

NEUTRAL REACTION: SOCIAL AWARENESS, ALREADY HAS GOOD RAPPORT :[L3]
IDEA VARIABLE: GETTING ENOUGH SOCIAL INTERACTION :[L3]
IDEA RELATIONSHIP: TIME SPENT ON SOLITARY WORK VERSUS SOCIAL INTERACTION :[L3]

He thinks of himself as a sensitive person who enjoys good rapport with people and thinks his friends and wife would agree. He thrives being around people

> "Even though I have a job and interests that are solitary a lot – if I get too much of that it's not a good thing – I need to balance, that's important for me. A detective might notice that I'm not getting the social that I need. I'm a new teacher, even though I'm 40, so there's a fair amount of extra work I need to do, and I'm writing a book, so there's a fair amount of work I need to do. So I need to balance that." :[L3]

He was a Franciscan monk for years and believes that experience helped him develop listening and counseling skills. He also believes meditation helps him be aware of his surroundings and people.

POSITIVE REACTION: WORK HABITS :[L3]
IDEA VARIABLE: SOCIAL VARIABILITY, TIME WITH DIFFERENT SETS OF PEOPLE (KIDS VERSUS ADULTS) :[L3]
IDEA VARIABLE: TIME WITHOUT DISTRACTION :[L3]
He noted that in his work, balancing time with kids versus adults is important, as is finding settings where he can work without distraction.

I asked him if he would find a report like this valuable and what in particular he would want the report to focus on.

BENEFIT: HELP MODERATE SELF-CRITICAL FEELINGS :[L3]
He would like the report to include eating behaviors, work habits, and social interaction – the latter two particularly because he is a new teacher and feels he is overly self-critical right now.

CONCERN: IF INTENTIONS OR COMMITMENT ARE QUESTIONED (SOCIAL, WORK) :[L3]
I asked him if there were anything he wouldn't want to hear or learn, anything that would make him feel uncomfortable. He indicated that if the person questioned his intentions toward and commitment to helping people and making their lives better would bother him.

[Place](0:23:00)
POSITIVE REACTION: ACTIVITY PATTERNS IN PLACE – MORE IMPORTANT IF LARGER HOUSE :[L3]
He found the activity patterns interesting and would like to see them over time. He believes he would be more interested if he was living in a house, as he and his wife are currently living in the smaller space of an apartment. He would like to see it over time.
IDEA INVESTIGATION: HOW MUCH TIME DO I SPEND ON THE COUCH WHEN I THIN K I'M REALLY WORKING :[L3]
> "How much time do I spend on the couch when I think I'm really working, that would be useful." :[L3]

IDEA USE: EXAMINING ACTIVITY PATTERNS ON MONTHLY BASIS :[L3]
IDEA USE: SPECIFIC AND AVERAGED ACTIVITY PATTERNS :[L3]
He thinks that he would look at it on a monthly basis, which he thinks would be long enough to make it into a consistent pattern. He would like to see both specific and average.

I asked him what we would see if we collected this data for the next month.
> "I think after hours, after 4 o'clock, you'd see me in front of the computer, you'd see me up late. My wife would probably be spending more time reading on the couch and working at the desk, but not with the computer on." :[L3]

IDEA INVESTIGATION: MAINTAINING AN EATING SCHEDULE, WHAT WAS HE DOING WHEN HE DELAYED EATING OR SKIPPED EATING, WHEN CAN HE DO PREPARATION TO HAVE SNACKS AVAILABLE :[L3]
He doesn't take medication, but he is trying to eat on a more regular schedule. He thought that it would show him working on correcting papers when he should be getting something to eat. He noted that part of the problem was that he prepares his snacks, so the time required to do that is a deterrent for getting a snack when he needs one. He notes that he could eat something like cottage cheese, but organizing grocery trips and preparation of snacks is still an issue for his busy life. He's gone to the trouble of getting small containers for snacks.
> "It could be useful – maybe more so in a larger home – but it's mostly about… I have a very work intensive eating habit. If I did TV-dinners, it would be pop-in microwave and turn-in microwave." :[L3]

BENEFIT: FOCUSING ON TAKING CARE OF YOURSELF (FOR PEOPLE WHO TRADITIONALLY ACT AS CAREGIVERS) :[L3]
> "It's about taking care of myself when I'm used to taking care of other people."

"It's about falling into old habits. I don't know if proximity would help that. If you were looking at larger patterns outside the home, that would be useful… it would be useful to see why didn't I eat at this day or that day, oh it's because I had a meeting at 4 o'clock. There are a lot of demands on my schedule… and I need to make sure I have food. Anything that would help with scheduling." :[L3]

He is planning working with some friends who are software engineers to make a tool that could provide meal suggestions and grocery lists to meet particular nutrition goals, tailored to one's BMI. He likes the idea of having biometric information related to your performance on the diet fed back into the tool. Even though he doesn't like needles, he would be willing to sample his blood for this purpose.

IDEA RELATIONSHIP: DIET AND HEART-RATE, BLOOD PRESSURE :[L3]

"At the end of the week, if I could measure heart rate, blood pressure, and… feed that back in..." :[L3]

IDEA USE: COLLECTED DATA COULD ALSO BE USED FOR NATIONAL STUDIES :[L3]

He goes on to imagine that the data collected could be supplied to a national database for researchers.

NEGATIVE REACTION: MOOD SELF-REPORT, ALREADY KNOW, DIFFICULT TO RECORD :[L3]

CONCERN: TOO MUCH LIKE START TREK :[L3]

"I don't know if I want to be going through the day… I'm picturing myself a mobile unit like my cell-phone, 'low point', I'm like registering it <laughs>, it's like I'm a member of Star Trek or something and I'm reporting back to myself. I'm not sure I want to do that to the level that is required to get useful information, because I can already tell you what those [situations that are low points/high points] are, off the top of my head, without monitoring. If someone wanted to get beyond that level of analysis about what people won't disclose or monitor about themselves, like an anthropologist would do without the intrusion, you would need something that could [passively] capture that…" :[L3]

CONCERN: DON'T WANT TO BE CONSCIOUS ABOUT ANALYZING SELF THROUGHOUT THE DAY :[L3]

"I would want to do that with minimal encroachment on my consciousness. I don't want to be conscious about analyzing myself all day long in quite that way." :[L3]

CONCERN: DISRUPTIVE :[L3]

IDEA USE: PROMPTS TO ENCOURAGE WHEN TO RECORD OR REFLECT, BUT MINIMAL SUGGESTION :[L3]

He wants tools that are self-initiated or have minimal intrusiveness.

"I use the prompts on my email to write, and I could use that moment to do this debriefing kind of thing. I need encouragements to do that, but those more readily come from in, and if I can't do that, I need minimal level of suggestion." :[L3]

[Social](0:40:35)

POSITIVE REACTION: SOCIAL NETWORK, FREQUENCY, TIME, QUALITY :[L3]

IDEA VARIABLE: MEANS OF MAINTAINING CONTACT, PHONE/EMAIL/WRITING :[L3]

"These would be quite useful. I've tried to do that myself. I've tried to create a spreadsheet, it sounds silly, it's so easy to get so busy, it would be useful to have something that would show me a pattern of connection. Frequency, time, and quality of that, all those factors. Means of maintaining as well, is it better for phone, email, writing." :[L3]

I asked him what he would discover about his time spent on social connections.

CONCERN: WOULD DISCOVER NOT SPENDING ENOUGH TIME (ON FAMILY CONNECTIONS) :[L3]

"There's not enough <laughs>. That I spend a lot of time with work and not enough time with family, with extended family, my mom or my siblings. None of them live here. It's not like we never talk, but I'm not in touch with my siblings nearly enough." :[L3]

IDEA USE: REWARDS OR PUNISHMENTS FOR NOT MEETING SOCIAL INTERACTION GOALS :[L3]

"You know what would be best? If I couldn't use my computer, unless I made a phone call or if my stove wouldn't work unless I made a phone call or my fridge wouldn't open unless I didn't make a phone call…. It sounds weird <laugh>, but it's like that sound your car alarm makes, when you open the fridge, it doesn't stop until you make that phone call. You may think that's crazy… but we need those kind of things. It could be humorous or use positive reinforcement – every time you call that person, you get a free chocolate bar <laughs>" :[L3]

[Sorting] (0:45:30)

doesn't drink alcohol very much
doesn't drink coffee/tea
doesn't feel old enough for grip strength
would like night vision compared to other people
doesn't have a pet, but would
has trouble sleeping
thinks he knows when he uses microwave, but may be wrong

He wouldn't want him something following him around for the stories/jokes, telling him whether he is funny.

IDEA VARIABLE: PERSONALITY/LEARNING STYLE (E.G., MYERS BRIGGS) :[L3]

He would be interested in patterns such as those revealed on the Myers Briggs test. He things he would see a change in the way you answer those questions. He's taken the test before with respect to learning style and found it useful.

CONCERN: FOCUSING ON INTROSPECTIVE QUALITIES MAY MAKE THEM WORSE :[L3]

"The irony of some of those things that are introspective is that you get in your own way by having the device around, it almost makes it worse." :[L3]

With respect to conversation turn-taking, he wouldn't want to live in a reality-TV type situation, but might find it interesting to be in a research study scenario.

CONCERN: BEING OBSERVED WILL AFFECT BEHAVIOR :[L3]

Based on his education in anthropology, he knows that being observed affects one's behavior.

CONCERN: EVEN VIDEO WON'T TELL YOU THE WHOLE STORY :[L3]

He's used videotaping as a tool in teaching and acknowledges its value and its limitations.

"The camera in some ways doesn't lie, but anything can only take as big a picture as the frame allows." :[L3]

CONCERN: OTHER FAMILY MEMBERS MAY NOT BE WILLING TO BE TRACKED :[L3]

He's not sure how his wife would respond to being tracked.

IDEA USE: NON-VIDEO TRACKING, PARTICULARLY TO PUT OTHER FAMILY MEMBERS AT EASE :[L3]

IDEA VARIABLE: QUALITY OF TIME, AMOUNT OF DOWN-TIME, VEGGING :[L3]

He would feel more comfortable with non-video tracking, particularly with respect to other family members.

"I would want to track diet and exercise pretty regularly and also the quality of my time, like how often am I sitting on the couch just vegging, how often am I wasting time that I could do other things. It's hard because I have an intensive job with a long commute, and I need down-time, but it would be interesting to look at how much of it I have." :[L3]

Resorts according to stress.

IDEA INVESTIGATION: UNDERSTANDING WHY WEEK WAS A STRESSFUL ONE :[L3]
IDEA RELATIONSHIP: STRESS AND SNACKING, RELATING TO PEOPLE :[L3]

"If at the end of the week, and it was really stressful, I could look back and some of it would be obvious, like I spent 17 hours in traffic, but other things like how much I was snacking, if I was eating properly, how I was relating to people, that would be useful." :[L3]

BENEFIT: UNDERSTANDING THE DEGREE TO WHICH KNOWN FACTORS AFFECT YOU (STRESS) :[L3]
BENEFIT: DISCOVERING FACTORS THAT AFFECT YOU (STRESS) :[L3]

"I think it would be both the degree of those things and things I'm not aware of." :[L3]

ID: [L4]

Date: 8/22/04, 2:00 – 3:15 (75 minutes)
Participant type: Friend to researcher
Demographics: Woman, 41 years
Location: Phone (New Jersey)
Audio file: DM100014

- Shown 6 Displays: Snoop, Daily Rhythm, Activity, Years,
- Strong concerns about tracking

Work: Housewife (with child under age 8)
Technologies Cell phone, digital camera, no pda
Tracking devices: Scale, pedometer

[What I monitor] (0:03:05)

CURRENTLY MONITORING: STEPS :[L4]

IDEA USE: TRACKING TO REACH A GOAL EACH DAY (PEDOMETER) :[L4]

She uses a pedometer to record the distance that she walks and runs. She does not currently record the values, instead trying to reach a particular goal each day. She is planning to begin running in races at some point in the future and believes that she would record the values in preparation.

She mentally monitors her family's needs in terms of groceries.

IDEA VARIABLE: HEARTRATE :[L4]

She would like to measure her heart rate.

IDEA VARIABLE: CALORIES :[L4]

She has kept hand-written food journals in the past, including caloric information. She found it difficult to record and would like a handheld device that would provide automatic calculations. She commented on how difficult it was to determine her caloric intake based on how foods are cooked and served.

> "What is a tablespoon of oil? If you sauté mushrooms, are you really factoring in the oil that you sautéed them in?" :[L4]

[Diary] (0:14:40)

PROCEDURE: MISINTERPRETATION OF DISPLAY AS SCHEDULE RATHER THAN RECORD :[L4]

She initially misinterpreted this example as providing a schedule for an upcoming day and reacted negatively. When I put the example in the context of providing a record of the day before, she reconsidered.

NEUTRAL REACTION: TIMES WHEN ENGAGING IN PERSONAL CARE, DON'T NEED :[L4]

She generally thought it might be useful, but felt the hygiene activity information was unnecessary.

> "Brushing my teeth for 2 minutes, I just don't see the necessity of that." :[L4]

POSITIVE REACTION: TIMES WHEN WALKING :[L4]
POSITIVE REACTION: FRIDGE OPENINGS :[L4]
POSITIVE REACTION: TV REMOTE :[L4]
IDEA USE: TO DETERMINE HOW MUCH TIME WASTED (USE OF TV REMOTE) :[L4]

“The walking, maybe monitoring, I might find the refrigerator one. I guess mine all would do with food and health. And the other I found useful was the TV remote one. [I would use it to] track the time I was wasting watching TV.” :[L4]

NEGATIVE REACTION: TIME SPENT ON DAILY ACTIVITIES :[L4]
CONCERN: HIGHLIGHTING WASTED TIME :[L4]

I asked her to imagine what it would be like to have several days of this information for the next week.

“Probably a lot of the time I waste. <laughs> That’s probably why I don’t want to see this.” :[L4]

CONCERN: FEELING A NEED TO COMPETE AND SET UNREALISTIC GOALS :[L4]

“What would make me feel better if it was directing me to do things like exercise. I guess it would have a lot to do with your self-esteem. Sure if you put in a day of getting things done, you are going to feel really great about yourself. I could also see this thing, if it was a part of my life, could be very depressing. You could start trying to compete against this thing and feel like you’re some kind of super-human.” :[L4]

[TV](0:18:00)

BENEFIT: HELP YOU MONITOR BEHAVIORS YOU WANT TO LIMIT (TV WATCHING) :[L4]
NEGATIVE REACTION: MOOD RATING, CONCERNS ABOUT PRIVACY :[L4]
CONCERN: PRIVACY :[L4]
CONCERN: FEEL LIKE A SCIENCE PROJECT :[L4]

“I have both positive and negative thoughts about it. I think in some way it is helpful in that it makes you look at how much time you are spending watching TV and how much time you’re spending outside. It could help monitoring your TV watching. I’m a little, I guess with all this… [the mood rating] that is too New World Orderish for me; I don’t want that information out there. I would be concerned how this is using against me. I’d feel like I was being a science project.” :[L4]

I asked her if she was just writing this information in your journal if she would feel the same way. She asked whether the information would be in her TV and I responded that at a basic level, it could just be her recording it, or the time could be recorded in other ways that are not connected with her TV.

CONCERN: OVER-ANALYZING :[L4]
NEGATIVE REACTION: TIME SPENT IN CONVERSATION WITH SPOUSE :[L4]
POSITIVE REACTION: MOOD RATING :[L4]

“I guess I would think this over-analyzing. Take out the conspiracy theories, and we’re just looking at it as my diary or my journal, I don’t think that I’m going to write about how much time I spent in conversation with my spouse. My mood rating, mmm… that’s probably something I would write in my diary.” :[L4]

IDEA RELATIONSHIP: MOOD AND TIME SPENT EXERCISING, EATING (REFRIGERATOR OPENINGS) :[L4]
POSITIVE REACTION: TIME SPENT WATCHING TV VERSUS TIME SPENT OUTSIDE :[L4]

I mentioned that this chart highlights how you can see relationships between variables, such as mood-rating. I asked her what she would like to see in relationship to her mood-rating, if she were to record it, and she said time spent exercising or eating – how many times her refrigerator opened.

“I would like to see how much time I spent watching TV versus how much time I spent outside.” :[L4]

POSITIVE REACTION: TIME SPENT WATCHING TV AND ESTIMATE FOR TIME WATCHED [L4]

She also thought it would be helpful to compare the time spent watching TV to her estimate.

POSITIVE REACTION: TIME SPENT WATCHING TV AND MOOD :[L4]
IDEA RELATIONSHIP: WEATHER AND MOOD, TIME SPENT OUTSIDE :[L4]
"I would also want to know what the weather was like outside. Either one [weather or TV] would affect my mood and my time spent outside. If it was rainy or snowing." :[L4]

[Daily Rhythm] (0:23:30) (participant chosen)
IDEA VARIABLE: TIMES ON PHONE, WORK VERSUS PERSONAL PHONE CALLS :[L4]
BENEFIT: LEAD TO A CHANGE IN BEHAVIOR :[L4]
IDEA VARIABLE: TIME SPENT ON COMPUTER :[L4]
CONCERN: LEAD TO DEPRESSION (TIME SPENT INSIDE) :[L4]
"I think in one way this looks at myself and how I spend my time. My relationships – how many times I'm on the phone. To me, you have to further divide what kind of phone calls these are – work or personal. This would really prompt me to look at how I was spending my time over the week. It's two sides of the coin, either it's going to spark in me a change or lead me into depression. If you saw that you spent all your time inside, not doing anything, spending all your time on the Internet. I think that would definitely make you feel more depressed." :[L4]

IDEA RELATIONSHIP: SPOUSE TRAVEL SCHEDULE AND TIME SPENT ON COMPUTER :[L4]
I asked her what we would see of her pattern.
"When [my husband] is out of town, you'd see more time on the Internet. Even though I might be spending some time doing research on my own, there's still that nagging feeling that you should be outside doing something more constructive." :[L4]

IDEA USE: INCLUDE DETAILS ABOUT WHAT YOU WERE DOING INSIDE, ON COMPUTER :[L4]
I asked her if there was additional information about what she was doing while inside that would make her less concerned.
"If you could enter what you were doing, sure. If you just saw a fixed time for how long you were inside all day, then I think it would have the opposite effect. I think I would need to find out what I was doing." :[L4]

CONCERN: OBSESSED OVER TIME LOST RATHER THAN HELPING INITIATING A CHANGE (TIME SPENT INSIDE) :[L4]
I asked her if she saw several days of just spending time inside whether that would encourage her to make a change for the next day to balance things out. She indicated that she would just stay focused on the days spent inside.
"It would make me agitated." :[L4]

NEUTRAL REACTION: WAKING AND SLEEP TIMES, STAY FAIRLY CONSTANT :[L4]
She wakes up at the same time each day and the time, which she goes to sleep only varies within a 2-hour block. Her husband is the same way. She does not therefore feel waking up/sleep time would be informative.

NEGATIVE REACTION: MEAL TIMES, TOO STRUCTURED :[L4]
POSITIVE REACTION (MILD): MEAL TIMES, CURIOUS :[L4]
CONCERN: FORCING STRUCTURE ON LIFE :[L4]
She was somewhat concerned that seeing the meal times would give her a sense that her life had to be too structured. She did think, however, that it might be interesting, but not particularly valuable.
"Depending on how it was presented, it might be interesting to see oh, here I am eating dinner at 6pm, on time. I would just be curious." :[L4]

[Commitments] (0:29:45)
IDEA USE: LARGER TIME SCALE MAKES LESS TENSE (COMMITMENTS)
POSITIVE REACTION: COMMITMENTS ON YEAR TIME SCALE
She liked the commitments example, particularly the yearly chart.

"I think yearly versus daily makes me less tense."

"I could see it as a positive way to make me start doing [the things I postpone]." :[L4]

She initially thought the place-based commitment was strange, but with some thought, changed her mind.
"It allows me to keep it in the forefront of my mind." :[L4]

CONCERN: ALREADY IGNORING EXISTING REMINDER SYSTEMS :[L4]
She noted that she gets AOL reminder alerts but always ignores them.

[Performance] (0:33:30)
POSITIVE REACTION: MEMORY :[L4]
BENEFIT: CONFIRMING POSITIVE SELF IMAGE (MEMORY) :[L4]
POSITIVE REACTION: STRENGTH TRAINING :[L4]
The first thing she noted as being of interest was the memory test. I asked her what she might see if she was seeing the information for the next year.
"[It would stay] consistent. I would think I have a sharp memory and [it would make me feel good]. So would strength training." :[L4]

IDEA USE: TRIGGER CONVERSATION WITH DOCTOR (MEMORY)
IDEA USE: MONITOR WHETHER KEEPING MIND ACTIVE IMPROVES MEMORY
She would alert her doctor if she saw that her memory was getting worse. If he or she told her it was a normal part of aging, she would keep on monitoring it.
"As I get older, I'm aware that your memory gets worse unless you keep your mind active." :[L4]

She would want the information even if it were disconcerting.

NEUTRAL REACTION: PROJECT SNAPSHOTS, DOESN'T NEED SENSE OF PROGRESS, DOESN'T THINK OF AS GETTING BETTER AT :[L4]
IDEA VARIABLE: PROJECT PRODUCTIVITY (WRITING PAGES) :[L4]
Her personal projects concern doing research on the Internet. She does not feel she needs a sense of progress, unless she was working on a book. In that case, she would like to see how much she had written. In general, she would want to track productivity against a goal. She did not think of her project in terms of "getting better at doing it." She feels she knows her subject better but does not need a representation of it.

IDEA RELATIONSHIP: PILATES WORK WITH WAISTLINE :[L4]
POSITIVE REACTION (MILD): TRAINER'S COMMENTS :[L4]
She would like to see how she is improving at her Pilates, defined in terms of inches lost. She works with a trainer and could imagine that she might keep comments that her trainer makes at sessions. She does keep a chart for strength training. It hasn't just steadily improved; she has seen dips in her performance.
"Usually there would be a dip, then there would be progress."

[Family] (0:41:15)
POSITIVE REACTION: MORNING SCHEDULE FOR KIDS :[L4]
BENEFIT: EVIDENCE FOR FAMILY DISCUSSIONS (CHORES NOT GETTING DONE) :[L4]
"It would be great to show, to use as an example if I didn't think certain things were getting done. Show my kids that they could use their time more wisely." :[L4]

CONCERN: KIDS WOULDN'T WANT INFORMATION – TEENANGERS MIGHT BECOME DEFIANT, ONLY OF INTEREST TO CHILD IF CONNECTED WITH REWARD :[L4]

She admitted that they her kids wouldn't like that information, however. She thought that it would make her teenager more defiant, but thought it could be helpful for her 6-year old, if rewards were integrated. She thought he would find it rewarding to see that he is meeting a goal, but could better understand how it impacted his allowance.

IDEA VARIABLE: TIME GOING TO SLEEP FOR KIDS :[L4]

IDEA USE: TRACKING TIED INTO ALLOWANCE :[L4]

> "He is horrible about getting to bed on time. With him, I think it would have to be in some form of reward or punishment [instead of in relationship to how tired he felt]" :[L4]

CONCERN: TOO MUCH INFORMATION :[L4]

CONCERN: FEELING WATCHED :[L4]

IDEA USE: IT'S OKAY FOR MY KIDS, NOT OKAY FOR ME OR MY SPOUSE :[L4]

> "I can see pros and cons to it. In some ways I think it too much information. Do I really need to know that [my husband] is in the shower at 7 and I'm feeding birds? I guess I would need this if I wanted to make the most out of every minute of my day. As far as children, I could see it as a good way of showing them how to make good use of their time. Maybe I'm being hypocritical… when I turn the tables on myself, I begin to feel watched." :[L4]

POSITIVE REACTION: WATER USAGE :[L4]

IDEA VARIABLE: ELECTRICITY :[L4]

IDEA RELATIONSHIP: WATER USAGE/ELECTRICITY AND MONEY SAVINGS :[L4]

She liked the water usage example and would also like to see electricity.

> "I guess what I would like to know is… I would think for someone like us, how that translates to savings in terms of money." :[L4]

POSITIVE REACTION: READING TIME :[L4]

IDEA RELATIONSHIP: TIME SPENT ON GAME BOY VERSUS READING (FOR CHILD) :[L4]

IDEA USE: NO-ELECTRONICS DAY :[L4]

She liked the reading time example.

> "We've talked about having a no-electronics day. I'd like how much time spent on game boy versus reading [for my son]." :[L4]

She liked the information about when she read and when the reading was with a parent. She thought it would be important for reading with her children, but not for her husband and herself.

CONCERN: TAKING OVER FOR PEOPLE THINKING (IDENTIFY SHARED THEMES AND NEW WORDS) [L4]

As far as the shared themes and new words, she thought it was too extreme.

> "That's just too nutty for me, I'm sorry. It's just too much, taking too much away from people thinking. No, I don't like that." :[L4]

NEUTRAL REACTION: EATING TOGETHER, ALWAYS EAT TOGETHER :[L4]

They always eat together so monitoring that is not an issue.

CONCERN: SPENDING TOO MUCH TIME REFLECTING/ANALYZING AND NOT LIVING :[L4]

> "I don't see that as necessary either. I'd feel like I was spending too much time with my diary and not being part of life." :[L4]

POSITIVE REACTION: GROUP ACTIVITY LEVELS, ONLY IF CRITICAL GOAL IDENTIFIED :[L4]

IDEA USE: ONLY TRACK WHEN DECIDE THERE IS A CRITICAL GOAL THAT NEEDS TO BE ACHIEVED (FAMILY ACITIVITY) :[L4]

She thought that her family could use the group activity levels. She would like to monitor her kids' activity levels, but would be less interested in her husband's.

"Group activity levels – I could see this stuff happening if okay, everyone in the family needs to lose some weight or get outside more, so for the next few weeks we're going to track our activity, but I don't know if I want this to become part of my everyday life." :[L4]

CONCERN: TOO MUCH DETAIL :[L4]
BENEFIT: IF FAMILY IS AT A BREAKING POINT AND SOMETHNG MUST BE DONE :[L4]

"I think it's a bit too much detail. I think this would be great if something was sorely lacking and we really needed to get on track if we were going to make it." :[L4]

[Mirror] (0:53:20)

NEUTRAL REACTION: TOOTHCARE, ALREADY MASTERED :[L4]
IDEA USE: TOOTHCARE FOR KIDS :[L4]

She thought that the brushing time and flossing would be helpful for her kids, but felt she had mastered this as part of her normal routine.

IDEA VARIABLE: DOING ORGANIZATIONAL TASKS :[L4]

I asked her if there was something like flossing for her, that she's trying to get better at doing.

"Getting things organized. Going through a box or something. I would love when I planned to do it, to do it that day… I would love a program that help me not procrastinate" :[L4]

IDEA RELATIONSHIP: ABILITY TO DO TASK (ORGANIZING) AND MOOD, ENERGY LEVEL, EATING, EXERCISING :[L4]

"For me, that would be related to mood… probably energy level, which might be directly related to what I eat, how I exercised." :[L4]

I asked her if she was planning on doing an organizing project this Thursday and was able to look back on aspects of days when she had been successful in the past, would she be able to see things that would help her prepare for Thursday in a way that would better guarantee that she complete her task.

BENEFIT: IDENTIFYING CONDITIONS THAT MAKE TASK COMPLETION MORE LIKELY :[L4]
IDEA RELATIONSHIP: ABILITY TO DO TASK (ORGANIZING) AND TIME GET UP IN THE MORNING, TIME FIRST EXERCISE IN MORNING, TURNING ON COMPUTER, GETTING SHOWER OUT OF THE WAY, COMPLETING MORNING CHORES, :[L4]

"The time I get up, that I first exercise in the morning, that I don't turn on the computer, that I immediately get my shower and get my morning chores out of the way, and that I don't have anything else going on." :[L4]

She agreed that there might be other things that affect her success that she might not be aware of and that doing tracking for that reason would be helpful.

[Sorting] (0:58:45)

Awareness of time bothers her.

She liked anything health related, such as bathroom trips and blood glucose.

IDEA RELATIONSHIP: BLOOD SUGAR AND CAFFEINE IN RELATIONSHIP WITH SLEEP QUALITY :[L4]

She would like to see blood sugar and caffeine in relationship with sleeping.

IDEA VARIABLE: VOLUNTEER COMMITMENTS :[L4]

She would like to determine how much time she puts in volunteer commitments.

"At this point, I'm very into anything health and fitness." :[L4]

She can be gullible, but is not sure how gullibility would be tracked.

She is anticipating menopause and feels that hormone levels/cycles tracking would be important.

IDEA RELATIONSHIP: TIME SPENT WATCHING TV AND TIME SPENT USING COMPUTER VERSUS TIME SPENT WITH CHILDREN AND TIME SPENT DOING THINGS FOR SELF (READING) :[L4]
She would like to understand how much time she is spending watching TV and using the computer, because she feels it affects how much time she spend with her children and doing things for herself, like reading.

IDEA RELATIONSHIP: IMPULSIVENESS AND SPENDING :[L4]
"Yeah [to impulsiveness], I'd like to track how many times I go outside and buy something." :[L4]

"As they get older, I'd like to know more about what's going on with them [her pets]"

She is interested in her hearing perception as she gets older.

IDEA RELATIONSHIP: PILATES AND POSTURE :[L4]
She's working on her posture with pilates.

IDEA RELATIONSHIP: REFRIGERATOR OPENING/CLOSING AND WEIGHT AND ELECTRICITY USE :[L4]
She saw that refrigerator opening/closing could give her information about watching weight and watching electricity.

She feels that she came from a generation that consistently burned themselves, so she is interested in skin changes for tracking possible skin cancer.

"I would love to monitor snacking." :[L4]

CONCERN: MONITORING MOOD IS TOO SUBJECTIVE :[L4]
CONCERN: MONITORING VARIABLES RELATED TO FAMILY MEMBERS IS INVASIVE :[L4]
"Spouse's mood I think is just too subjective. It strikes me just as too invasive."

IDEA RELATIONSHIP: SPORTS PERFORMANCE AND SLEEP :[L4]
She thought that if she got more serious about racing, she would want to track time at which she went to sleep.

IDEA RELATIONSHIP: TIME SPENT COOKING AND FREQUENCY OF EATING OUT :[L4]
She would like to see time spent cooking in relation to eating out.

"Personal relationships and stuff, I don't think it's necessary to keep track of that." :[L4]

IDEA RELATIONSHIP: TRIPS TO GROCERY AND SPENDING AND TIME USE :[L4]
She relates trips to grocery to money spending and time use.

"I think in most instances we know if we are spending too much time, but I would like to know." :[L4]

IDEA USE: IDENTIFYING PRODUCTIVE VARIATIONS FROM ROUTINE :[L4]

“Variation from routine. Yeah I would like to know that… if I went away from my routine and was more productive or less productive. If I was more productive, then there might be something in my variation that I need to apply.” :[L4]

She thinks of use of space as organization.

Resorting by family relationships. **(1:12:00)**

PROCEDURE: RESORT ON FAMILY RELATIONSHIPS, ADDS CARDS OTHER FAMILY MEMBERS SHOULD TRACK :[L4]
She was thinking about all members of the family, and that's why she added awareness of time. Chores and getting things done

IDEA RELATIONSHIP: TIME SPENT WITH FRIENDS VERSUS TIME SPENT WITH FAMILY (TEEN DAUGHTER) :[L4]
With her daughter, she thought it would be helpful to contrast time spent with friends versus time spent with family.

Final thoughts (1:17:30)
CONCERN: ANALYTICAL ASPECT OF TRACKING DOES NOT REFLECT PERSONAL IDENTITY :[L4]
“It's weird. I'm not the kind of person – I have a day organizer and sometimes I use it, but sometimes I don't. I rely a lot on my memory.” :[L4]

“I would rather use my memory than have something to do it for me.” :[L4]

CONCERN: THE REAL PROBLEM IS DENIAL AND NOT MISSING INFORMATION :[L4]
“I think everyone has in the back of their mind, a bell that goes off, they know when they're spending too much time doing something. You've been watching too much TV, slacking for the last few days because I'm in a mood, because something is bothering me. I think they already know that.” :[L4]

CONCERN: MAY BECOME OVER DEPENDENT ON TRACKING AND RISK AFFECTING MENTAL ABILITIES :[L4]
She is concerned that if we do not use our memory and rely too much on technology, we are more at risk of losing mental ability.
“…it takes away from our memory.” :[L4]

IDEA USE: TOOLS THAT TRIGGER MENTAL ANALYSIS (PEDOMETER) :[L4]
She uses the pedometer value to trigger her to re-analyze her running.
“I'm using it more as a gauge, not as a science.” :[L4]

CONCERN: REINFORCING IDEA THAT NOT LIVING UP TO WHAT YOU'RE SUPPOSED TO BE :[L4]
“The two points I would point out is that it causes undue stress, we already live in a stressful society and seeing how our performance is, it's almost like we're not living up to what we're supposed to be, so it put stress to perform a certain way; and it's putting more reliance on technology and less on ourselves. So those are my two negative comments.” :[L4]

She thinks the benefits of tracking depend on personality type and imagines that there are some people who would want to do it.
“Maybe I'm just not a Type A personality.” :[L4]

CONCERN: TRYING TO DO IT YOURSELF WHEN YOU SHOULD BE SEEKING OUTSIDE HELP FROM A TRAINED PROFESSIONAL (THERAPIST) – VARIABLES RELATED TO FAMILY RELATIONSHIPS :[L4]
“I could see the benefit to family relationships. If you are going through a really hard family time and you need to look at some of these things – but maybe you should be going to a therapist instead of handling it yourself.” :[L4]

CONCERN: DOESN'T MATCH MY "TRADITIONALIST" PERSONALITY :[L4]

"I just know that there are always going to be people who want gadgets and there are people who are traditionalists, and I'm a traditionalist." :[L4]

BENEFIT: AMUSEMENT :[L4]

CONCERN: TRIGGERED CHANGES TO BEHAVIOR WOULD NOT LAST :[L4]

"For ha-has, I might want to track some of this stuff. Would I implement them? Would they make me change how I do things? Maybe for a time, I don't know if it would be a long-term change." :[L4]

ID: [L5]

Date: 7/1/04, 12:04-1:08PM (64 minutes)
Participant type: Participant, through email announcement
Demographics: Woman, 41 years
Location: By phone (Oregon)
Audio file: DM1000

- Shown 4 Displays: Journal, Snoop, Daily Rhythm, Mirror

GENERAL REACTION: POSITIVE RESPONSE TO TRACKING, WITH SOME CONCERNS (+/-) :[L18]

- Keeps a journal with both subjective and objective information
- Sees tracking as a way of recognizing accumulation of incremental changes and shifts in pattern – particularly with regard to how approach problems or issues
- Reacted with concern about the Mirror example, noting that it could make people self-critical or obsessed with the past

Work: TV producer and preschool teacher
Technologies: Uses a cell phone, but does not own a digital camera or PDA
Tracking devices: Does not use a scale, pedometer, glucose monitor, or heart rate monitor.

[What I monitor] (0:05:50)

CURRENTLY MONITORING: WAY CLOTHES FIT :[L5]

CONCERN: A PARTICULAR METRIC (WEIGHT) CAN DISTRACT FROM BIG PICTURE :[L5]

"I keep track of my health, just not in terms of the number on the scale. The way my clothes fit tells me more about whether I'm in shape or not than a scale. I found that the scale is very distracting." :[L5]

CURRENTLY MONITORING: PET ACTIVITY :[L5]

IDEA USE: USING DOG'S HEALTH TO MOTIVATE PERSONAL HEALTH (WALKING) :[L5]

"I try to keep track of how many times I exercise or walk. I tend to keep track of it in terms of *my dog's health*. You know what I mean? My dog needs to be walked twice a day. Then I'm walking twice a day. Then it doesn't become oh god, I have to go work out, instead it's I have to go take Jackson for a walk. If I don't walk her, then I don't walk, and we both know we're getting out of shape." :[L5]

CONCERN: ACTIVITY IS SO ROUTINE, NO NEED TO TRACK (YOGA, SPORTS) :[L5]

"In terms of yoga or swimming or things that my dog doesn't participate, I have an awareness of it, but I'm not writing it down – I've done it long enough." :[L5]

BENEFIT: MOTIVATION TO REACH GOALS :[L5]

"I don't need that motivation right now, but there have been times in my life when I've written it on the calendar so I can look back on it and go, oh come on, if I do it today, I'll get a perfect 5 this week." :[L5]

CURRENTLY MONITOR: CHRONOLOGICAL RECORD OF EVENTS AND EMOTIONAL REACTION :[L5]

She keeps a journal, which includes both a chronological record of events and her emotional reactions to those events.

"It's a mixture of personal experiences and a record keeping of what has happened. We went on vacation and kept a journal where we were emotionally narrating, but also chronologically narrating what we did on the trip. So we would be able to remember it, but also what it felt like to be walking on the lava of the big island of Hawaii…" :[L5]

She uses her non-digital camera a lot and her boyfriend has a digital camera.

"Photographs are probably the most consistent way I document what's going on in my life, and making lists, that's more sort of preparation and reminding sort of stuff, might be work related, might be personal, might be exercise." :[L5]

CURRENTLY TRACKING: JOB SEARCH EFFORTS AND OUTCOMES :[L5]

She doesn't look back on her list unless she's trying to motivate herself, in which case she keeps them in her journal. When she's looking for a job, for example, she notes who she contacted and the people to whom they refer her.

"When I'm trying to be methodical about something, I use a list as a way to track and document my method." :[L5]

[Journal] (0:13:35)

BENEFIT: PROVIDING A SENSE OF IMMORTALITY :[L5]

"Part of it comes down to a sense of your own immortality. How important is it for the world, or even me, to remember my life minute by minute. There have been times in my life where, oh my god, I have to remember everything. In which case, at that time in my life, oh my god yes, every person I've met, every place I've visited. It's all so important I just have to remember it all." :[L5]

CONCERN: WHAT DO YOU NEED TO HOLD ONTO ABOUT YOUR LIFE EXPERIENCE :[L5]

POSITIVE REACTION: MEMORABLE MOMENTS :[L5]

"Right now in my life – I've moved a lot in the last two years and my first thought is I'd have to carry this big book with me <laughs>. What I think is important to hold on to, or to remember, to carry with me at this point in my life, has really, really winnowed down to where I was like 5 years ago. In that context, the memorable moments would probably be the thing that would stick out the most." :[L5]

POSITIVE REACTION: WATER DRINKING :[L5]

"The water drinking, comically enough, is something that I wouldn't necessarily want to remember for ever, but it is something would be useful to keep track of, because I know the amount of water I drink is very important." :[L5]

IDEA VARIABLE: INTERACTION WITH DOG :[L5]

"I'd probably add a category, because of my dog and her consistency in my life." :[L5]

NEUTRAL REACTION: SNACKING :[L5]

She would like a chronology of the day on one side and what was memorable about it on the other.

"I would add a category called daily activities, which would just be the chronological accounting of just what happened that day, as opposed to be places visited or people encountered, but it would be like, got up, medicated, worked with so and so, went to work and did x – then that in conjunction with memorable moments would be all I would want to keep track of. 'Cause I think if I had a particularly memorable meal, it would go in memorable moments, but for me snacking or food consumption wouldn't be something I would want to keep track of, unless it was a memorable meal." :[L5]

IDEA VARIABLE: MEMORABLE MEALS :[L5]

A memorable meal could be something enjoyable or horrible ("oh my god, remember that meal?").

For a while she kept a journal that adhered to this structure, with a chronological record of the day and a journal that served as an emotional companion, "I was thinking this, I was feeling this, I remember this, it triggered this." She reviewed the journal and would look for patterns.

BENEFIT: IDENTIFYING PATTERNS AND SHIFTS IN PATTERNS (APPRECIATING INCREMENTAL CHANGE) :[L5]

BENEFIT: PUTTING CURRENT CHALLENGES IN PERSPECTIVE :[L5]

"The value of keeping it for me is being able to go back over it to both identify patterns and to be able to notice shift in patterns, because I think the problem with incremental change is that when you're in it, it's really hard to observe it. If you are keeping track of it, you can go, oh you know what, 6 months ago I would have done x if I was confronted with that situation, now I notice that I did y, so I am changing, slowly but surely. Without that documentation, it's really hard not to get caught up in your own experience of the moment and to think, oh my god I never change and it's horrible." :[L5]

[Snoop] (0:19:35)
"I think it would all be really useful. Sure that would be awesome."

CONCERN: DISCOMFORT FROM HAVING ILLUSIONS ABOUT YOURSELF QUESTIONED (COULD ALSO BE A BENEFIT) :[L5]
IDEA USE: ONLY GET SNOOP-STYLE INFORMATION OCCASIONALLY :[L5]
"It would probably make me all feel uncomfortable <laughs> There's nothing like someone giving you unbiased information about yourself to make you feel uncomfortable. That's the essence of our own illusions… but I think it would be really useful. I wouldn't want it to go on all the time, every now and then a little update would be useful. Sure it would be fascinating." :[L5]

CONCERN: TRUST SOURCE OF OBSERVATIONS, KNOW BIASES AND PERSPECTIVES (SNOOP) :[L5]
"I would have to know and trust the source though, because so much of what is being observed is subjective rather than objective. I would want to know the biases and the objectives of the person who was observing so I could have a framework for their comments. Some LA workout group is looking at my life, they are going to look at it differently than some Buddhist yoga instructor, who is going to look at differently from some overweight couch potato. How they are going to see my opportunities for exercise, my work habits, or my social awareness, they are going to be framed by their standards. I'd like to know who it was observing and what they were bringing to it." :[L5]

"Good eye contact. I've done a lot of work with Native American kids, as a preschool teacher, and in their culture, you don't make eye contact with elders. A lot of the kids spend their time with their eyes down. Stuff like that – what does good eye contact mean." :[L5]

IDEA USE: PROVIDING OBJECTIVES TO OBSERVER :[L5]
POSITIVE REACTION: SOCIAL AWARENESS :[L5]
"If I was to say to them, hey I'm hiring you, you know whoever, I'm hiring you, [L18], and here are my objectives for the week, and if you could tell me the ways in which I am achieving them and ways in which I am not, that would be very useful. If they were my ally and they were trying to keep me to my standards that would be great. I could say you know, I really want to work on my eye contact… or like the *you didn't pick up Julie's nervousness…* I could say, sometimes I'm worried that I am oblivious to my co-workers, so if you notice I want to know about that. I could say, oh I know Julie and she always wigs out and it's best to ignore her, otherwise we'll spend an hour talking about her nervousness when we should really be getting going on it. But it could be that oh, I never noticed that, I noticed that she's being quiet, but I didn't think to ask how are you doing, Julie." :[L5]

For some week in the near future, a sample objective would be. **(0:23:50)**
IDEA VARIABLE: FINANCES :[L5]
"Well like if I want to be more disciplined about keeping track of my finances – I notice the whole finances piece is kind of missing from what you're, well we haven't talked about it – that's a big issue as far as discipline goes. If I want to keep track of my finances, that would be useful, it would fun to have someone say, hey [L5], you've

missed these opportunities to be more disciplined, either to keep track of or to report back on, or that kind of thing. Finances would be one piece." :[L5]

POSITIVE REACTION: OPPORTUNITIES FOR EXERCISE :[L5]

"I also think the opportunities for exercise is always a good one, because it's fun to go, oh yeah I guess I could have walked, I didn't *have* to drive today." :[L5]

POSITIVE REACTION: POSTURE :[L5]

"I think that the posture <laughs>, anybody tall, and maybe short people too, but I'm tall, so it's okay, I'll check the posture one too." :[L5]

IDEA VARIABLE: WAYS TO INTEGRATE WORK INTERESTS (I.E. WORKING WITH KIDS AND TV PRODUCTION) :[L5]

"That's when it gets really personal. For me it would be related to some of the goals I'm setting for myself and how am I achieving, so for me right now it would tend to be work related and personal exploration related, because that's what I'm working on, how do I integrate these two aspects of my work into some *work.* How do you integrate working with kids with TV production, without making you know, Teletubbies." :[L5]

IDEA VARIABLE: WAYS YOU PROCRASTINATE, MISSED OPPORTUNITIES TO DO WHAT'S IMPORTANT TO YOU :[L5]

"I'm really curious about the ways I avoid things I don't want to do. I'm curious about the ways I procrastinate. I'm obviously really good at it <laughs>, because I can fool myself there's nothing else I to do but procrastinate, when it's been three days since I've done something I didn't want to do. I suspect someone else looking at me, and my boyfriend sometimes does, could say you know, [L5], there were these 10 points when you could have done that thing you say you are dying to do but just can't find time to do. That would be useful." :[L5]

[Daily Rhythm] (0:27:30) – chosen by participant

The participant brought up the movie "Kitchen Talk" about Scandinavian researchers who observe bachelors to better design kitchens.

POSITIVE REACTION: DAILY RHYTHM :[L5]
IDEA VARIABLE: HOW EFFICIENTLY USING TIME :[L5]

"This would be cool to have. I like the chart part of it. I like charts. It's graphically very interesting to look at my chunks of time. *That* would be something I would like to know better, how efficiently I am using my time, and this chart looks like it would be a really useful way to get that." :[L5]

She related efficiency of time to procrastination.

IDEA RELATIONSHIP: DINNER AT HOME VERSUS DINNER AT A RESTAURANT VERSUS ORDERING TAKE-OUT :[L5]
IDEA RELATIONSHIP: TIME GETTING HOME AND MEAL PREPARATION :[L5]
IDEA RELATIONSHIP: EXPECTATIONS FOR MEAL PREPARATION, SPOUSE PATTERNS, MOOD :[L5]

"For example, you don't have it on here specifically, if you were charting dinner, dinner at home versus dinner at restaurant – you could look at this and go Friday was dinner out because dinner is the middle of them both being out. Same with Saturday. It would be interesting to chart that in terms of, so when my spouse, in terms of this picture, is out until 10 minutes before dinner, that's when we eat take out food, but when we're both home an hour beforehand, that's when we make dinner… and like on Tuesday, when I rush in, but my spouse has been home, then that's when he takes the time to make a really good dinner. It's sort of like, in terms of efficiency, how much are we ordering pizza, how much are we ordering take-out, how much when we do eat out, that's sort of stuff. It would be cool to – the pattern thing, I like being able to look at this and go, oh no wonder I'm so grumpy on Tuesday nights, first of all I don't get home until 6:30 and second of all, Jim's always home and dinner's never ready. Every Tuesday this happens and I know he's been home all day because he only has one class until two. Being able to see patterns is kind of cool. " :[L5]

(0:32:20)

NEGATIVE REACTION: POSTPONED COMMITMENTS :[L5]

CONCERN: GETTING DISCOURAGED (PROCRASTINATION) :[L5]

IDEA VARIABLE: ACCOMPLISHED TASKS :[L5]

"This is the something that I would say I want to track, but I suspect I really wouldn't. This is where the fine line between what I know I'm supposed to say and what I know is true about me would kick in. I *know* I should want to track how much I procrastinate, because that would help end the problem, wouldn't it. If I had to stare at this and say, oh my god [L5], you have procrastinated to do your finances 17 times in the last 10 days. But the truth is – would I really do it, if it was mine and at my house, probably for the first month I would, and then I would get really discouraged. Having to face my own postponements would get discouraging. I'd rather get a list of what I've accomplished <laughs> and kind of go, woo-hoo, I did 10 things on my list, now I need to get the motivation to do the other 4. For me, looking at what I have done helps me to find the motivation to tackle something hard." :[L5]

IDEA USE: SUPPORT GROUP FOR COMMITMENTS :[L5]

She used to be part of a friendship support group that helped her follow through with personal commitments. The commitments could concern work projects, financial goals, or family relationships.

"One of the useful things I have done recently is to create a friendship group that is related to making commitments to each other, and I don't have it now, this is a couple of years back, where we would get together every two weeks and literally make a commitment to the group about one thing we would accomplish in the next two weeks. And that was really useful." :[L5]

[Mirror] (0:38:00) – participant chosen

She thought that the timing values related to bathroom activities were amusing and very MIT. She agreed that the time to get information about your recent flossing activity was at the time when you were next brushing your teeth and after correcting the brushing teeth time goal (it should be closer to 2 minutes), recognized it as an incremental goal.

POSITIVE REACTION: FLOSSING RECORD :[L5]

IDEA USE: QUANTITATIVE NOT QUALITATIVE → UP TO PERSON TO DECIDE HOW TO USE :[L5]

"This is very cool. I love the flossing record thing, that would be handy, because I would floss more if I got a little nudge. Maybe you could have your floss so it would track every time you took the floss out of the container. This is interesting because this is objective. This is interesting because it's just useful information for me to have. Quantitative not qualitative, this is just reporting back to me. It's saying okay [L5], it's been two days since you last flossed, what's up, and then it's up to me. It's not saying, you've done a great job flossing or a bad job flossing. It's different from that thing that is following me around. This is more just data that I can do with what I want to." :[L5]

NEGATIVE REACTION: REFLECTION :[L5]

CONCERN: PRESSURE TO STAY YOUNG (REFLECTION) :[L5]

She had concerns about the mental health implications of seeing a reflection of yourself in the past.

"For me, this would be a little alarming <laughs>. I'm not madly in love with the whole reflection, I don't spend a lot of time looking in the mirror or thinking about what I should wear. I could see how it would be a source for a lot of negative messages for how we look. That's not necessarily a reason not to do it, but I think we, particularly as women get a lot of pressure to look younger, even as biologically the exact opposite is happening, and to be reminded in the morning what I looked like a year ago – you know what, I looked younger. I think that we as a culture have a complicated enough relationship with our mirrors, that this one raises some flags for me." :[L5]

CONCERN: MAY MISINTERPRET DATA (CHANGE IN APPEARANCE FROM SLEEP, STRESS, EATING, EMOTIONAL EVENTS, NOT JUST WHAT YOU WERE WEARING) :[L5]

"How you look has a lot to do with how much sleep you got, how much stress you're under, how much you ate. It's different from a record of what you wore – see a picture of your face, you could have been pregnant, your dog could have just died…" :[L5]

CONCERN: LIVING IN PAST (REFLECTION) :[L5]

"It's like a photograph, there are a lot of people who spend a lot of time either hating what they look like and reminiscing about what they need to do to get back to that day. I've got a bit of bias toward Buddhism and being in the moment, and going you know what, that's just where you were then, this is where I am." :[L5]

CONCERN: STRIVING TOO HARD FOR PERFECTION (REFLECTION) :[L5]

"So let's say you had the perfect good hair day, and that's what you have a picture for, how much time do you want to spending trying to get your hair to look perfect again? That was a great day, and wahoo you should enjoy it, but now that you have a zit because you are PMSing – you wake up in the morning and you see the perfect hair day and then you see the zit for this morning and suddenly what happens to your confidence for that meeting. I don't see a lot of value in that piece of looking back." :[L5]

POSITIVE REACTION: REVIEWING EVENTS AND PEOPLE ENCOUNTERED :[L5]

CONCERN: METHOD OF DELIVERY FOR DATA REVIEW, FINDING RIGHT TIME AND PLACE AND NOT GETTING IN THE WAY :[L5]

IDEA USE: REVIEWING INFORMATION WHILE EATING BREAKFAST :[L5]

She commented on some of the awkward aspects of displaying information, such as the activities from the previous day or the people encountered/will encounter, on a bathroom mirror.

"I know for people who wear contact lens, they'll spend a lot of time squinting at the mirror, because they don't have their contact lens in. It also tends to get foggy… In terms of a place to disperse information – you might as well put a ticker thing, you know a stock ticker thing, like a little billboard in the shower. <laughs> I'm joking sort of, but when you think of places where you're standing with not much else to think about, for me the shower ranks a little higher than – usually when I'm in front of the mirror, I'm going through the routine, brush my teeth, check my hair, blah, blah, blah, I'm not really needing to stop and read anything right then. The flossing thing was nice because it was a quick graphic thing – *alright, fine, I'll floss*. In the shower or on the toilet, there are more opportunities for me looking for reading material, than when I'm front of the mirror. I'd rather have this in my kitchen table, this would be great to read when I'm eating breakfast. On a table or on a tablet that I carry with me, so I could glance at this and kind of make my plans for the day." :[L5]

She agreed that mornings are the time she would want to review this information.

[Sorting] (0:51:00)

Resorted by physical activity

ID: [L6]

Date: 7/7/04, 7:36-8:27AM (51 minutes)
Participant type: Participant, through email announcement
Demographics: Woman, 41 years
Location: Participant workplace
Audio file: DM100034

- Shown 6 Displays: Journal, Snoop, Grocery, Daily Rhythm, Body, Voicemail

GENERAL REACTION: POSITIVE REACTION, SPECIFIC INVESTIGATIONS (+/-) :[L6]

- Is a runner and is interested in activity-related metrics
- Interest in tracking impulsive spending
- Likes the idea of sharing data between aging parents and children

Work: Financial analyst, manages money, picks stocks
Technologies: Regularly uses a cell phone, digital camera, and PDA
Tracking devices: Uses a scale and a heart rate monitor

[What I monitor]

CURRENTLY MONITORING: CALORIES :[L6]
She counts calories, but doesn't keep a record.

She does not keep a journal or diary, but does have photographs. She keeps a schedule to keep appointments, and occasionally looks back on, but not routinely. She stays focused on what is ahead.

CURRENTLY MONITORING: RUNNING INTERVALS :[L6]
She is a marathon runner and uses a digital watch to monitor running intervals.

[Journal] (0:06:37)

POSITIVE REACTION: MEMORABLE MOMENTS :[L6]
POSITIVE REACTION: SNACKING :[L6]
POSITIVE REACTION: WATER DRINKING :[L6]
IDEA USE: TRACK ONLY WHEN WANTED TO KNOW :[L6]
IDEA USE: TRACK TO UNDERSTAND RECENT CHANGE (WEIGHT) :[L6]
NEUTRAL REACTION: PEOPLE ENCOUNTERED :[L6]
NEUTRAL REACTION: PLACES VISITED :[L6]
She would like to keep a record of memorable moments, but those may not be on a daily basis. She would only want to track snacking and water drinking at times when she would want to know, such as when she is trying to understand why she is gaining weight.

"So I would say, these are the most – memorable moments and then I think there would be times when you would want to know snacking and water drinking, but I wouldn't care about these [people encountered, places visited], unless I was writing a book <smiles>." :[L6]

IDEA RELATIONSHIP: SNACKING AND WEIGHT :[L6]
Wouldn't regularly check on snacking and water drinking.
"Say you gained a few pounds and you were trying to analyze why, you might want to track how much snacking you're doing. So it would be for purposes of collecting data that you could make a decision with." :[L6]

BENEFIT: FUN TO REMINISCE :[L6]

"Memorable moments would be like, wow, that was so fun, and you want to reminisce. But that might not necessarily be everyday, just wow, that was a memorable moment. Intervals of time that go by, friends in town or just went out with a group of people for dinner and had a good time." :[L6]

[Snoop] (0:08:50)

POSITIVE REACTION: EATING BEHAVIORS :[L6]
POSITIVE REACTION: POSTURE :[L6]
POSITIVE REACTION: EXERCISE OPPORTUNITIES :[L6]
BENEFIT: HELP YOU SEE THINGS YOU DO HABITUALLY :[L6]
BENEFIT: HELP YOU THINK OUT OF THE BOX ABOUT HOW YOU COULD IMPROVE :[L6]

"I think all those [Eating, Posture, Exercise] are good. Those are things you do habitually. Posture especially, you can't see yourself." :[L6]

"These are all things you would want to improve. We could all eat better, we could all have better posture, unless we're dancers. We all tend to work in office buildings, unless you're a park ranger, hiking up and down the park all day, you're not getting enough exercise, pretty much, by definition. So you're probably not thinking outside the box about a lot of these things and it helps to have input." :[L6]

Thinks social awareness and work habits are useful, especially if you are trying to "improve your status."

POSITIVE REACTION: SOCIAL AWARENESS – FOR JOB CLIMBING :[L6]

"Socially, you may not realize, but you're going for a job interview and you don't make good eye contact and you don't have a good command of the language and you fall into patterns of language use. People who know you, love you, and they accept it. I know one person who always finishes people sentences, always, and I don't think he's aware of it. It's the kind of thing he really should correct." :[L6]

POSITIVE REACTION: WORK HABITS :[L6]

"At work, it's so easy to fall into habits that you don't even realize." :[L6]

IDEA USE: ONLY TRY TO CHANGE A FEW THINGS AT ONCE :[L6]

"I think you can only change a few things at once. You'd want to work at one thing, or maybe two things at a time, and not massively change your habits." :[L6]

IDEA USE: EVENT-TRIGGERED TRACKING, GOING ON A JOB INTERVIEW, STARTING TO DATE :[L6]

She thinks you would know what to prioritize to improve and mentions particular trigger events, such as going for a job interview or date.

[Grocery] (0:12:40)

IDEA VARIABLE: SPENDING BY CATEGORY (EATING OUT, ENTERTAINMENT, RENT, ETC.) :[L6]

She would like to track where she is spending her money into categories, such as meals out, entertainment, rent, insurance, then there is discretionary spending ("wow, I spent $100 at Trader Joe's, on what?").

"I think it's very useful to track your spending. I've tried doing that, but it was a big burden." :[L6]

BENEFIT: SEEING HOW SMALL ACTIONS ADD UP (E.G., PURCHASES) :[L6]

"I think that's another thing that's very subconscious. You go into 7-eleven and buy a coke and don't think about it, but then when you start adding it up." :[L6]

IDEA USE: SEEING HOW BEHAVIOR CHANGE COULD RESULT IN OTHER GAINS (E.G., SPENDING) :[L6]

“If you saw that changing that behavior resulted in something really positive that you wanted to get – gee, if I got one less Starbuck’s a week, I might go to more movies or go out to dinner.” :[L6]

She has used a little a notebook, but it was too hard to put in a database for sorting and analyzing.

She agreed with the grocery spending example, but wanted to go beyond groceries (e.g. shoes at Bloomingdales, magazines). She uses “Carrie Bradshaw” as an example of someone who spent a lot on shoes that could have been used for a down-payment.

IDEA USE: IDENTIFY NEW CATEGORIES, SUCH AS NEW SOURCES OF SPENDING, THAT YOU WANT TO TRACK RIGHT NOW :[L6]

She would like to be able to add new categories, and gives the example of spending money on newspapers or how much you’re spending on gummy bears if you have kids.

“Time will dictate the different things that you are curious about. [It should be] customizable.” :[L6]

NEGATIVE REACTION: NUTRITION GOALS – DIFFICULT TO APPLY TO ACTUAL MEALS THAT ARE CONSUMED DUE TO COMBINATION OF FOODS AND PORTION SIZE :[L6]

She is not interested in nutrition goals on the grocery receipt because it is difficult to apply to particular choices on day, including portion size and combination.

[Body] (0:19:15) – participant choice

She’s interested in sports training and so chose the body example.

POSITIVE REACTION: SKIN RECORD FOR TRACKING POSSIBLE CANCERS :[L6]
NEUTRAL REACTION: HEALING TIME :[L6]

Thought the skin record would be helpful if you are tracking something like melanoma, but wouldn’t care about healing time.

IDEA USE: INVESTIGATION INITIATED AFTER CLINIC VISIT (SKIN CANCER) :[L6]

“I think if there is something you are monitoring for medical purposes where you’d have to go in to a doctor, they have to try to remember what they saw the last time. If you had a way, just at home, we want you to monitor this for 6 months, if every week you stood in front of the camera, took a photo of your elbow, then you’d have a record you could show your doctor. That’s useful. It wouldn’t be on a daily basis, it would just be when something turned up.” :[L6]

IDEA USE: HAVING TOOLS AVAILABLE, EVEN IF NOT USING ALL THE TIME (SKIN RECORD) :[L6]

“If you had a system, you’d just fire it up and use it when you needed.” :[L6]

NEUTRAL REACTION: ACTIVITY :[L6]
POSITIVE REACTION: MUSCLE TONE – OUT OF CURIOSITY, ONLY EVERY 6 MONTHS :[L6]

She wasn’t interested in activity, but was curious about how muscle tone would be measured.

“I’d do it [check muscle tone] out of curiosity, and maybe check it 6 months later because I could, but I don’t think I would go out of my way to do it.” :[L6] (0:21:51)

POSITIVE REACTION: EXERCISE LOG, LAST TIME DID BUT NOT REPETITIONS :[L6]
CONCERN: TIME REQUIRED TO RECORD :[L6]

She would possibly keep a record of the exercise she did, as long as it was simple and not time-consuming. She noted that most people keep a record at a gym. She doesn’t like doing repetitions,

but she might like to know the last time she did a particular exercise and would like to track it over time, particularly if it were weight-lifting.

She thinks aches would be useful as you get older.

POSITIVE REACTION: ACHES AND PAINS :[L6]

IDEA RELATIONSHIP: PAIN AND EXERCISE, STRETCHING, MENSTRUAL CYCLES, SLEEP, TRAVEL, SHOES :[L6]

IDEA USE: TOOLS THAT HELP YOU ANSWER A QUESTION :[L6]

BENEFIT: EASY TO OVERLOOK BASIC CONTRIBUTING FACTORS (E.G., PAIN BECAUSE OF SHOES YOU ARE WEARING) :[L6]

> "I would try to correlate it with other information I had. Say I had these backaches and they only came once in a while. I'd say, gee what else happened during that time? Did I work out hard and my muscles are tight? Did I do no yoga and just running, and that made my back sore, or did I have my period and I'm bloaty and crampy and that's the correlation. Or did I not get enough sleep and I was traveling. I think all of this stuff is useful if you have a question you need to answer and need a way to collect the data to answer your question. You want to be organized about it and you want it to be simple. You don't want to have to write a script, you want to be able to put things together easy and be able to say, gee headaches correspond with my period… as if you wouldn't know that, that's an easy example, but I wonder about those things, my back will be tight and I'll wonder, did I run particularly long or maybe I need new running shoes. You're trying to answer questions and you try to think about all the datapoints, but you don't usually have that information. You rely on gut feeling and you are probably overlooking something pretty basic." :[L6] **(0:24:00)**

When asked if she could just visualize it, but without a clear answer to what is occurring, would that still be useful, and she said yes.

IDEA USE: CLEAR ANSWER NOT NEEDED, RECOGNITION OF COMBINATION OF FACTORS OKAY :[L6]

> "I bet most things are a combination of things…. I think if things are customizable and you can track data that you want to track, for whatever questions come up in your life." :[L6]

[Daily Rhythm] (0:27:49)

NEUTRAL REACTION: DAILY RHYTHM :[L6]

She doesn't think she would use it that much, but it could be helpful at the right time.

> "Maybe there would be some things you'd always monitor, your weight or your blood pressure, or something. Then like, if you are fatigued for some reason – gee, while I am so tired – then you might decide to keep track of this for a while and see if anything shows up. You may discover something." :[L6]

IDEA USE: SOME THINGS YOU WOULD ALWAYS MONITOR, SOME THINGS MONITOR ONLY WHEN PROBLEM OCCURS OR HAVE QUESTION :[L6]

IDEA VARIABLE: WEIGHT, BLOOD PRESSURE, AND CHOLESTEROL :[L6]

She could imagine always tracking weight, blood pressure, and perhaps cholesterol if it could be easily done. She would like to track exercise, such as how much she is running.

CONCERN: TOO MUCH DETAIL :[L6]

CONCERN: NEED ABILITY TO AMEND BECAUSE AUTOMATED OBSERVATION NEVER 100% RELIABLE :[L6]

She felt that the daily events (time of meals) were too much detail and would be too time consuming to record.

> "The more automated, the better, with the ability to amend, because automated things are never 100% reliable." :[L6]

NEUTRAL REACTION: COMMITMENTS :[L6]

She wouldn't use the commitments-tracking, but likes the idea of having all of these available.

“This could be like a problem solving tool, like a little therapist that is helping you investigate something in your life.” :[L6]

CONCERN: PEOPLE WHO AREN'T READY TO CHANGE/DON'T WANT TO CHANGE WON'T BENEFIT :[L6]

“I’m a very data-oriented person, very organized, methodical person. I gravitate to this kind of thing. I think a lot of people aren’t discipline enough. They don’t really want to change. They don’t want to get better. They like sitting on the couch and drinking Coke and watching TV. But if they are really determined to change, having good information is important.” :[L6]

When asked if she has something that she resists changing, she couldn’t think of anything. She wants to get better at things she already does. Is she getting enough variety in what she eats, does she effectively replenish after a run, is she getting enough sleep – but it’s not “mission critical.”

[Voicemail] (0:36:00)

I suggested the voicemail example.

BENEFIT: PEACE OF MIND ABOUT AGING PARENTS :[L6]

POSITIVE REACTION: MONITORING CHANGES IN AGING PARENTS :[L6]

She liked the idea of monitoring changes in her aging parents’ health, particularly things they may not be aware of or tell her on their own, particularly if one of them died.

IDEA VARIABLE: PARENTS NOT GETTING OUT OF BED :[L6]

“If one of them stayed in bed for four days, without a good reason… it would be useful to have an objective system to monitor their health.” :[L6]

IDEA VARIABLE: PARENTS’ MOOD :[L6]

IDEA VARIABLE: WHETHER PARENTS HAVE TAKEN MEDICATION :[L6]

She would like to track her parents’ mood.

“A daily report would be awesome. Knowing that the medication was logged in. If I could call them, hey mom, I don’t think you took your medication?” :[L6]

BENEFIT: SUPPORTING COMMUNICATION BETWEEN FAMILY MEMBERS :[L6]

IDEA USE: IF DEPRESSED, HAVING FAMILY MEMBERS KNOW ABOUT MOOD SWINGS MIGHT HELP COMMUNICATION :[L6]

She feels she’s an open book and that there is nothing she is worried about health-wise or mood-wise, but if she was depressed, she might want family members to know when she was going through mood swings for communication.

She doesn’t think it would be awkward to have a conversation with her parents about things that are monitored.

[Sorting] (0:39:15)

Resorted by eating choices

ID: [L7]

Date: 6/18/04, 9:36-10:52AM (75 minutes)
Participant type: Participant, through poster
Demographics: Man, 45
Location: House_n
Audio file: DM100021, DM100022

- Shown 6 Displays: Journal, Snoop, Daily Rhythm, Years, Family, Place

GENERAL REACTION: POSITIVE, INTEREST IN EXPLORATORY AND APPLIED INVESTIGATIONS (+) :[L7]

- Is currently utilizing the tracking capabilities of email and a personal calendar on a cell-phone
- Interested in exploring patterns in daily living
- Nice observations about the challenges of applying tracking data to family issues
- Interested in identifying how much "me time" he has
- Concern about whether tracking mood would affect mood for the worse

Work: high-tech, programmer
Technologies: Cell-phone, digital camera, no PDA

[What I monitor] (0:01:00)

IDEA VARIABLE: PLACES VISITED :[L7]
IDEA VARIABLE: FREQUENCY EATING OUT :[L7]

"I don't explicitly track anything, but one thing that kind of interested about your project is that I'm always amazed, at least interested, when I get my phone bill or when I get my frequent flyer account statement. Oh yeah, I do go there – or my credit card bill… oh yeah, I've eaten out an awful lot these days, I should probably curtail that." :[L7] **(1 file, 0:01:00)**

NEUTRAL REACTION: UTILITIES, STAY CONSTANT :[L7]

"My utility charges are pretty constant; I'm never surprised by that." :[L7] **(0:01:51)**

Keeps a journal and also considers email a kind of journal.

IDEA USE: EMAIL AS A JOURNAL :[L7]
INVESTIGATION: CONVERSATIONS IN THE PAST :[L7]

"Recently I've had occasion to go back and read email from decades ago, and that was pretty interesting. I gained a lot of insights into who I was and how I've changed and how I got here…. I had reasons for doing it. I had to deal with someone's perceptions of me that had become my perceptions, but then there was some facts that came out of discussions with this person that just didn't jive with what I remembered. This is pretty important, I bet I told someone about this, and the only record I have is email that I sent to friends – and I looked back and said, well yeah, the way I remembered it was pretty much how it happened. I learned a lot doing that and it helped straighten my head out." "I just keep my email for work reasons and it gets mixed up in personal stuff… it wasn't something I intended to keep." :[L7] **(0:02:06) [what do you track]**

[Journal] (00:05:45)

POSITIVE REACTION: PEOPLE ENCOUNTERED :[L7]
POSITIVE REACTION: MEMORABLE MOMENTS :[L7]
POSITIVE REACTION: MEDIA, BOOKS READ :[L7]

In his journal, records people encountered and memorable moments, things he reads

"The one time I was disciplined about keeping a journal, I kept a daybook, summarizing things I read. Could be anything, I'm reading all the time." :[L7]

IDEA USE: KEEP TRACK OF WHETHER CHECKED OUT ARTICLES THAT HAVE BEEN RECOMMENDED BY FRIENDS/COLLEAGUES :[L7]

Only looks at news articles if someone points him to it – would like to remember that he checked it out.

IDEA RELATIONSHIP: EMAIL AND PHYSICAL LOCATION WHEN WRITTEN :[L7]

Would like to have some context to email (where was I when I wrote this?)

"One thing I noticed from my email and my journal is that there is not a lot of mention from where I am. I came across one email that didn't make a lot of sense to me until I realized I did it in Mexico." :[L7]

NEUTRAL REACTION: SNACKING :[L7]

NEUTRAL REACTION: WATER DRINKING :[L7]

Doesn't care about what he eats or drinks (snacking, water)

[Snoop] (0:10:25)

POSITIVE REACTION: WORK HABITS :[L7]

NEGATIVE REACTION: SOCIAL AWARENESS :[L7]

CONCERN: BECOMING TOO SELF-CONSCIOUS :[L7]

Has found it useful when boss or co-worker has taken an interest and given feedback on work habits; is sure would find social awareness interesting, but afraid it would turn him into a "nervous wreck"

"I don't think I need anymore self-consciousness. I think I have more than enough of that." :[L7]

NEUTRAL REACTION: EATING BEHAVIORS :[L7]

NEUTRAL REACTION: EXERCISE :[L7]

Not concerned about eating, posture, exercise.

[Daily Rhythm] (2 file, 0:01:05)

POSITIVE REACTION: DAILY RHYTHMS :[L7]

BENEFIT: FINDING UNEXPECTED PATTERNS :[L7]

Thinks daily rhythm would be interesting, not sure what it would tell, but believes something would come out of it. When he looked back on his email, he had the experience of finding things he didn't expect and wasn't looking for.

INVESTIGATION: EXPLORING PATTERNS WITHOUT A GOAL IN MIND :[L7]

IDEA RELATIONSHIP: MOOD AND SLEEP PATTERNS :[L7]

IDEA RELATIONSHIP: TIME SPENT WITH PEOPLE AND MOOD/OUTLOOK :[L7]

"I think I might discover something. For instance, it would be interesting to see, for example, maybe correlate this with the journal to see how sleep patterns affect your overall mood or the degree of, amount of time you spend with other people affects your outlook." :[L7]

IDEA VARIABLE: REGULARITY OF HABITS :[L7]

"Just to see how regular one's habits are or not." :[L7]

POSITIVE REACTION: DAILY RHYTHMS COMPARED WITH A FAMILY MEMBER'S :[L7]

Would definitely want to see his one pattern in relation to other people's patterns ("oh yeah, oh yeah. When you added that overlay it suddenly got a lot more interesting.")

[Commitments] (0:04:17)

POSITIVE REACTION: POSTPONEMENT OF COMMITMENTS :[L7]

"I already tend to do this. I don't have a PDA, but my phone has PDA functions and I do fill up my phone once a year and I dump calendar data to Outlook. That's always been interesting, how much time I spend on various things, how recurring items come up, and they almost have to do with paperwork, financial filings, mailing proxies, talking to my advisor – they are on my schedule and they tend to get postponed, for weeks at a time before I actually fulfill them." :[L7]

CONCERN: DOESN'T GUARANTEE CHANGE IN BEHAVIOR :[L7]

He indicates that it should change the way he approaches his financial tasks, but it doesn't. He schedules social commitments, but did think of one kind of commitment that he doesn't explicitly schedule.

IDEA VARIABLE: THINGS THAT YOU DO FOR YOURSELF :[L7]

"Things that I'm going to do for myself fall by the wayside, because I don't schedule them. That was actually my new year's resolution to schedule more things for myself, to give myself the same, or at least some weight, less than my sons or my work, but some – it's another new year's resolution I haven't followed up on." :[L7]

[Years] (0:08:44)

POSITIVE REACTION: CABINET ACCESSES FOR INVESTIGATING EFFICIENCY OF SPACE :[L7]

He has been thinking about the efficiency of kitchen – might like some time-motion in kitchen.

POSITIVE REACTION: SHORT-TERM MEMORY :[L7]

CONCERN: TIME REQUIRED TO TRACK :[L7]

He reacts to the short-term memory examples.

"I really feel like my short-term memory has fallen off a cliff in the last couple of years and that concerns me. I can't imagine actually taking the time to track it. Making the time to do it." :[L7]

CONCERN: ALREADY KNOW (TIME SPENT WITH FRIENDS) :[L7]

CONCERN: NOT VALUABLE (STOVETOP VERSUS MICROWAVE) :[L7]

Thinks he has a sense of the other things (time spent with friends) or doesn't care (stovetop versus microwave).

IDEA VARIABLE: TIME SPENT WORKING :[L7]

IDEA VARIABLE: TIME SPENT DRIVING :[L7]

"I think it would be interesting to have some record of the time I spent working, or the time I spend driving, which has always been a goal of mine to minimize." :[L7]

BENEFIT: DETERMINING WHEN A CHANGE (WEIGHT, BLOOD PRESSURE, TEETH) STARTS OCCURRING :[L7]

BENEFIT: UNDERSTAND NATURE OF CHANGE, DETERMINING GRADUAL CHANGE VERSUS SUDDEN CHANGE :[L7]

IDEA VARIABLE: TEETH :[L7]

Sudden change over years **(0:11:28)** – He mentions "health things" such as weight, blood pressure, the state of his molars – thing he doesn't pay attention to until he goes to the doctor and they say, did you notice? ("last time you weighed 30 more pounds – could be bad, could be nothing.") He believes it would be helpful to know when the change happened – whether it was gradual or just happened in the last three months. He never felt a need to have a scale.

[Family] (0:14:08)

POSITIVE REACTION: MORNING SCHEDULE :[L7]

BENEFIT: PROVIDING EVIDENCE IN FAMILY DISPUTES (MORNING SCHEDULE) :[L7]

IDEA RELATIONSHIP: TIME GOING TO SLEEP AND TIME WAKING UP – CHILDREN :[L7]

CONCERN: CAN USE DATA TO SUPPORT INTERPRETATION REGARDLESS AND BECOME ENTRENCHED :[L7]

"[the morning schedule] may help settle some family disagreements. Like what time we're really going to bed at night and what time we're waking up in the morning. It has been my assertion for a long time that my sons wake up later when they go to sleep later.

They don't believe that. They don't believe those two times have anything to do with each other. I have actually kept track for weeks at a time, but it doesn't convince them. The real problem with families trying to deal with issues of schedules and commitments to each other in the household is not really, I'm not sure it could be addressed technologically. It's not a matter of fact, it's a matter of interpretation. So everyone will find the facts to support their interpretation of what's happening and the more facts you gather, the more it tends to just cement the positions of the people. That's been my experience." :[L7]

CONCERN: FINDING TIME TO REVIEW INFORMATION AS A FAMILY :[L7]

"The other real problem is getting the time together to do this, just to talk about it, especially as the kids get older and don't want to sit a the dinner table. There's no time to set aside and say this is important." :[L7]

NEUTRAL REACTION: WATER CONSERVATION – NO PERCEIVED NEED :[L7]

IDEA VARIABLE: ATTENTION GIVE TO OTHER FAMILY MEMBERS :[L7]

The participant could imagine a discussion about water conservation in the future, but not now.

"The only really scarce resource is attention. There is a limited amount of attention that you can demand of yourself and others. That you can spend on others. I don't know how you would track or ration that." :[L7]

CONCERN: NON-ACTIONABLE, CAN'T DO ANYTHING ABOUT IT (EATING TOGETHER) :[L7]

He indicated that his family doesn't have any time to eat together, so it's not useful to track.

"It seems like there should be something you could do about it, but knowing that it's not happening doesn't help much. Or I just couldn't see it helping." :[L7]

CONCERN: BALANCE BETWEEN PLANNING AND SPONTANEITY :[L7]

I suggested that perhaps "opportunities" for sharing time together or attention

"You spend more time arguing about something than it would take to do it. Similarly, you spend a lot of time planning for things you'll never do, rather than doing things that you would do. There's this real tension, in family life as I've lived it and with my friends, as they've lived it, between planning and spontaneity. Because there are lots of things that you can only do or would only do if you plan them, but there are a lot of opportunities to do things that you miss, because you're planning or you're carrying out something that you planned to do, and you tend to pass up other opportunities. So, again I don't know, I haven't really thought about this. I don't know what you would do to get some insights into that." :[L7]

NEGATIVE REACTION: NO USE FOR INFORMATION (CORRESPONDENCE WITH FRIENDS/FAMILY) :[L7]

"I think this, to some extent is, this gets monitored in passing, or recorded in passing. I kind of know what I'm doing, and I know what the kids are doing, because they are still at that age, because they're not doing it unless you're planning it for them or with them. I don't know what I would do if I had this information." :[L7]

[Place] (0:28:18)

POSITIVE REACTION: CURIOSITY ABOUT REFRIGERATOR OPENINGS (TIME SPENT WITH REFRIGERATOR OPEN) :[L7]

"I have often wondered, how often the refrigerator gets opened without anything going in or coming out of it, but that's just <laughs>, my own curiosity. I just notice, occasionally I'll find someone standing in front of the refrigerator looking in there <laughs>." :[L7]

NEGATIVE REACTION: ACTIVITY SEQUENCES IN SPACE :[L7]

CONCERN: WOULD RATHER HAVE EXPERT EVALUATE DATA (HOME DESIGN) :[L7]

CONCERN: ALREADY KNOW (ACTIVITY PATTERNS IN SPACE) :[L7]

Although he mentioned it earlier as something of interest for home design, he would rather have an expert interpret the data. He feels like he knows enough about it.

“I have the impression that I know how the house is used and I don’t think I would learn anything from knowing in more detail about its used.” :[L7]

“You know where you are cleaning, so you know that during the week there’s a lot more activity at the front door, and on the weekend, at the back door.” :[L7]

IDEA VARIABLE: ACTIVITY SEQUENCES OUTSIDE THE HOME :[L7]

“Outside the home, maybe, but in the home, I have trouble seeing the utility.” :[L7]

[Sorting] (0:32:25)

IDEA VARIABLE: WINE PREFERENCES :[L7]

He interpreted alcohol drinking as “wine preferences”

ID: [L8]

Date: 8/27/04, 6:30 – 7:30 (55 minutes)
Participant type: Word of mouth
Demographics: Woman, 1956
Location: Phone (from Washington)
Audio file: DM100028_1

- Shown 8 Displays: Journal, Snoop, Family, Social, Weight, Body, Years, Daily Rhythm
- Interested in investigating lifestyle changes now that she is divorced and her children have moved away
- Interested in social variable

Work: Manager in shipping industry; divorced with adult children; one child moving home again
Technologies: Cell phone, digital camera
Devices: Bathroom scale

[What I monitor] (0:02:10)
She keeps a sheet of financial assets and liabilities and her checkbook.

CURRENTLY MONITORING: INTENSE TIMES IN LIFE (DIVORCE, PREGNANCY) :[L8]
Used to keep a journal off and on, but right now she isn't keeping one. She would record thoughts, actions, stuff that was going on in her life and of the people around her that affected her. She kept it during her divorce. She also kept journals during her pregnancies.

[Journal](0:04:25)
POSITIVE REACTION: PLACES VISITED, NEW PLACES (TRAVELING) :[L8]
POSITIVE REACTION: PEOPLE ENCOUNTERED :[L8]
POSITIVE REACTION: MEMORABLE MOMENTS :[L8]
She identified places visited, people encountered, and memorable moments as the types of information she would record in a journal if she were disciplined. The other stuff is not a "big deal" to her.

NEUTRAL REACTION: SNACKING (NOT OBSESSED WITH HEALTH) :[L8]
NEUTRAL REACTION: WATER DRINKING (NOT OBSESSED WITH HEALTH) :[L8]
"As I've gotten older in life, it's more the experiences that are important to me than like snacking and water drinking – I'm health aware, but I'm not obsessed. To me, I feel healthy so I don't feel a need to track them." :[L8]

She was thinking of the places visited with respect to travel locations, not mundane stops. She would list that she went to a festival or found a new trail to hike.

"If I had interaction with someone that was meaningful, if something was going on in their lives that touched me or affected me, I would probably list it." :[L8]

NEUTRAL REACTION: MEDIA (ONLY INTERESTED IN PERSONAL VARIABLES) :[L8]
Aside from big events like September 11th, media wasn't important to her. She wanted the journal to be about events in her life and in her family's life.

[Snoop](0:08:28)

“Oh I would be afraid of what they would say! <Laughs>” :[L8]

POSITIVE REACTION: EATING BEHAVIORS :[L8]
BENEFIT: SEEING HOW LIFESTYLE REQUIREMENTS AFFECT ROUTINES (EATING) :[L8]
IDEA INVESTIGATION: HOW EATING ROUTINES HAVE CHANGED WITH CHANGES TO HOUSEHOLD :[L8]

“Eating behaviors would be interesting, because now that I’m living alone, I think my eating habits have changed, and I would be curious with probably what someone else might observe about them. I’m not disciplined – when you have a family, you all sit down together and you have a prepared meal – I’ve found myself often standing at the counter eating a bowl of cereal for dinner.” :[L8]

NEUTRAL REACTION: POSTURE (FEEL HEALTHY ALREADY) :[L8]
NEUTRAL REACTION: EXERCISE (FEEL HEALTHY ALREADY) :[L8]

The health things – posture and exercise – weren’t very interesting to her because personally she feels healthy. She starts to tell me that she has an exercise regimen that she feels comfortable with, but then notes that she’ll be talking with a trainer next week, so she will be getting an outside opinion on it.

POSITIVE REACTION (STRONG): SOCIAL AWARENESS :[L8]

“I’d be really interested in social awareness, because I’ve always felt very social and I’d be curious about their observation.” :[L8]

I asked her whether she would have them look for specific things and she said she would prefer observations without her guidelines.

NEUTRAL REACTION: WORK HABITS (ALREADY HAVE STRONG WORK ETHIC) :[L8]

She feels she has a strong work ethic.

[Family](0:11:15) – participant choice

NEUTRAL REACTION: EATING TOGETHER, DETERMINED BY SCHEDULES, CAN’T CHANGE :[L8]

She chose family because her family arrangement has changed so much in the past few years. One of her daughters is moving home in the coming week. She noted that many of these examples don’t apply very well because her daughter is grown. They both will have their own schedules and won’t be eating together.

POSITIVE REACTION: CORRESPONDENCE WITH FAMILY, MEDIUM :[L8]

She regularly corresponds with family and believes that would be interesting to track. She finds herself using email a lot and can’t remember the last time she wrote a letter. It would be interesting to her to see the medium of correspondence.

[Social](0:15:05) – participant choice

She was thinking of something else when she saw the social example and said that she wasn’t sure that what was shown would be of interest to her.

NEUTRAL REACTION: TIME SPENT ON PHONE WITH FRIENDS AND FAMILY :[L8]

“Interaction with other people. I’m in a lot more social situations because I’m divorced now… that’s how you get out there. To me, social is a little different connotation, it’s being out there with people, striking up conversations in a café or standing in line. I go out dancing quite a bit.” :[L8]

IDEA VARIABLE: EFFORTS TO MEET NEW PEOPLE :[L8]

She would like to track information on her efforts to meet new people.

"For me it would be interesting in how many – I try to expose myself to new people, so like if I go into an elevator, I at least say something to the person, like great August. I like people and you never know who you are going to meet. So to me that was what I was thinking." :[L8]

[Weight](0:18:23) – participant choice

She thought that the weight example would be fun.

POSITIVE REACTION: WEIGHT :[L8]

IDEA RELATIONSHIP: PERSONAL APPEARANCE, WEIGHT, FEELING GOOD, ACTIVITY :[L8]

"That would be kind of interesting for me. Actually, my weight doesn't vary much, but I'm real active and some days I *feel* better than others. I don't know if I look better or not, but you're right some days I feel are good days and some days I feel are bad days. Sometimes it has to do with stepping on the scale and sometimes it has to do with my hair not looking the way I want it to." :[L8]

IDEA RELATIONSHIP: EXERCISE AND ENERGY LEVELS, HOW WELL CLOTHES FIT :[L8]

BENEFIT: ALTERNATIVE WAYS TO MEASURE PROGRESS WHEN ONE VARIABLE (WEIGHT) DOESN'T CHANGE :[L8]

She's going to a personal trainer so that she will guide her to where she wants to be. She will judge progress by energy level and how her clothes fit. She's noticed that when she works out a lot, she may not lose weight but her clothes fit differently.

[Body] (0:20:45)

POSITIVE REACTION: ACHES AND PAIN (CARPEL TUNNEL, SHOULDER PAIN) :[L8]

She would like to start tracking pain especially as she gets older. She doesn't get headaches, but she has had carpal tunnel syndrome in the past and pain in her shoulder. She went to a physical therapist for her shoulder.

She believes she usually she has a good sense of the cause of the pain or discomfort, such as when she experienced back ache a few weeks ago after gardening.

POSITIVE REACTION: ACTIVITY, WHEN WORKING WITH TRAINER :[L8]

She thought her trainer would keep her log of what activity she gets and she might start doing that.

NEUTRAL REACTION: WRINKLES – PLANNING TO GROW OLD GRACEFULLY :[L8]

IDEA VARIABLE: BRUISING – HOW MANY AND HEALING TIME :[L8]

She plans to grow old gracefully, so she won't worry about the wrinkles. Her mother has a history of bruising easy and not healing quickly from the bruises. She hasn't noticed that in herself yet, but would like to track it given her family history.

[Years] (0:24:25)

INVESTIGATION: FACTORS THAT CONTRIBUTE TO A DIVORCE :[L8]

IDEA RELATIONSHIP: SPOUSE TRAVEL SCHEDULE AND HEALTH OF RELATIONSHIP :[L8]

Because her life has changed so dramatically in the last 10 years, with a divorce, the kids moving out, and job changes, she thought there would have been some valuable things to track to document the changes.

"If I could go back and track how things have changed, one of them might be the relationships, the time that I spent with my now ex-husband and family, how that time changed. Lot of that problem was his travel schedule, which left me alone a lot with the kids. I think that was one of the reasons we ended up getting divorced, the time apart. If I could track his travel schedule with where things were in our relationship." :[L8]

She considered her 47th birthday as the first day of the rest of her life.

"With the changes in my life, I know there have been big shifts. I'm now looking forward, instead of back." :[L8]

IDEA VARIABLE: QUALITY OF RELATIONSHIP WITH KIDS, TIME SPENT WITH KIDS, QUALITY OF CONVERSATIONS :[L8]

She would like to track the relationship with her kids as they get older, specifically the time they spend together and "how the conversations mature and change as we mature and change."

IDEA VARIABLE: ENERGY LEVELS FOR DETECTION OF DEPRESSION :[L8]

I asked about whether there are things that precede or coincide with times in her life when she is feeling down. She thought her energy level

"I wasn't on top of my game. My body for whatever reason was putting its resources elsewhere. In those times in my life when things have been, a bad day compared to a good day, my energy goes down. It's a sign to me that I need to workout or do something where I feel like I'm accomplishing something." :[L8]

Whenever she's down, she tries to choose tasks that are doable to give her a sense of accomplishment and to get her energy levels kick started.

BENEFIT: CONFIRMATION OF WHAT YOU ARE FEELING DURING DEPRESSION/STRESSFUL TIME PERIODS :[L8]

"Especially when I was going through the divorce – obviously that's a very dramatic time in your life, but if there had been someone there to track that, and just confirm a lot of the stuff that I was feeling, that might have been really useful." :[L8]

NEUTRAL REACTION: SHORT-TERM MEMORY OVER LONG-TERM :[L8]

POSITIVE REACTION: TIME SPENT WITH FRIENDS OVER LONG-TERM :[L8]

She wasn't interested in short-term memory, but was interested in time with her friends.

"I've found myself more hooked on time with my friends." :[L8]

[Daily Rhythm] (0:31:40)

POSITIVE REACTION: DAILY RHYTHM :[L8]

IDEA INVESTIGATION: COMPATIBILITY OF FAMILY MEMBERS ON SLEEP/WAKE SCHEDULES :[L8]

She was interested in daily events and sleep schedules.

"I'm not a morning person and my daughter is definitely not a morning person. Something like that might be interesting to see how compatible we are. That one might be kind of fun." :[L8]

POSITIVE REACTION: COMMITMENTS, POSTPONEMENTS :[L8]

When we discussed the commitments example, she brought up an example of a project she's been meaning to do, but hasn't gotten done – organizing a box of photos. There are some things she gets down on a regular basis and some things she postpones, and she would be curious to see a breakdown of what those things are.

[Sorting] (0:33:50)

Ability to concentrate would not serve any purpose for her

She wanted to see how her ability to recall as she gets older – she says she is good with names, but her kids like to tease her that her short term memory is bad

IDEA RELATIONSHIP: ALCOHOL DRINKING AND CHANGES IN SOCIAL PATTERNS :[L8]

She thinks alcohol drinking would be interesting to monitor because her life has changed – if the patterns have changed

She thought that bathroom trips may be relevant in a few years

She has had no issues with diabetes, so blood glucose is not of interest

She would like to track blood pressure because she is trying to be more health aware

IDEA RELATIONSHIP: CLOTHING CHOICES AND PLACES VISITED :[L8]

She thought clothing choices would be interesting because she leads a varied existence and would be curious to see her clothing choices compared to where she was going
She doesn't drink coffee
She would like to track commitments to see if she is using her time well
She thought that conversation turn-taking would be interesting to track once her daughter moves home to see if they are communicating at equal levels
She feels she currently tracks email and correspondence
IDEA RELATIONSHIP: GRIP STRENGTH AND EARLY STAGES OF SERIOUS DISEASE (CANCER) – IMPLIED? :[L8]
She thought that grip strength would be relevant as she gets older and referenced that her parents were both cancer survivors
She says that her kids tell her that she's "easy to put one over on," so it would be interesting to track gullibility, but she thought she wouldn't like the results
She believes she's nearing menopause and is thinking about getting one of those over-the-counter tests to find out when she'll be going through it
CONCERN: DON'T WANT TO TRACK THINGS THAT ARE TOO MUNDANE, THAT HAPPEN EVERY DAY (USING COMPUTER) :[L8]
She uses the computer every day and is not interested in tracking
CONCERN: FRIENDS/FAMILY ATTRIBUTES WOULD BE INTRUSIVE :[L8]
She thought that tracking how much she knows about her friends and family would be intrusive
She feels like she is aware of moods but would like to check it
IDEA RELATIONSHIP: IMPULSIVENESS AND LIFESTYLE CHANGES :[L8]
She would like to see impulsiveness given the changes in her life
Laughing and smiling is a good indication of how she's feeling
Doesn't do knuckle cracking
IDEA RELATIONSHIP: MULTITASKING AND DIVISION OF CHORES, HEALTH OF RELATIONSHIPS :[L8]
She relates multitasking to division of chores and would like to have tracked it with respect to her spouse and her children as she feels it was one of the problems in her marriage (she was doing everything)
IDEA USE: TRACKING SOMETHING THAT IS NEW AND FUN (NEW DIGITAL CAMERA – PHOTOS TAKEN) :[L8]
She just got a new digital camera, so was interested in the number of photos taken
She thought that raised voices would have been interesting to track when her kids were younger
IDEA INVESTIGATION: GOOD DAYS AND BAD DAYS WITH SHORT-TERM MEMORY :[L8]
She would be curious about short term memory as she gets older and noted that sometimes she feels it's getting worse and sometimes she feels on top of her game
IDEA RELATIONSHIP: SKIN CHANGES AND TIME SPENT IN THE SUN :[L8]
Skin changes with respect to too much time in the sun
Doesn't smoke
Will ask her boyfriend if she snores, but doesn't think she does
IDEA RELATIONSHIP: RESPONSE TIME AND SPORTS PERFORMANCE :[L8]
Sports – skiing stamina, skiing response time
She knows she is a night person
She doesn't cook very much
Her job includes sales, so she spends a lot of time driving – was it worth the trip?
IDEA RELATIONSHIP: GROCERY TRIPS AND HOUSEHOLD CHANGES :[L8]
She doesn't go grocery shopping as much now that she's single
IDEA RELATIONSHIP: USE OF SPACE AND LIFESTYLE/HOUSEHOLD CHANGES :[L8]
She is in a large house now and finds herself confining herself to just a few rooms – so she would like to track her presence
IDEA RELATIONSHIP: VARIATION FROM ROUTINE AND LIFESTYLE CHANGES :[L8]
Variation from routine as a single life

She resorts by physical activity.

She's a lightweight in terms of alcohol
The need to set aside time for working out

IDEA INVESTIGATION: PEOPLE'S MOODS TO UNDERSTAND SOCIAL SITUATIONS (WHEN TO START CONVERSATIONS) :[L8]

She would like to get better at detecting whether the people she is working out with are open to conversation

> "When you are thinking of a particular goal, when I applied these to physical activity to me, it seemed to be easier to say no, yes, or maybe because you're applying it to something specific." :[L8]

IDEA INVESTIGATION: CHANGING ROLES/RELATIONSHIPS OF PARENTS AND CHILDREN AS THEY BECOME ADULTS :[L8]

> "There are some times when this really might be interesting to me, especially with my daughter returning to the nest. To track how much time I spend with her and how much time I spend picking up after her, so some of that stuff might be real interest to track and I might think about doing something like that. Even though I haven't tracked, compare it to how things were when she was home before she left." :[L8]

ID: [L9]

Date: 8/27/04, 10:00 – 11:00 (45 minutes)
Participant type: Word of mouth
Demographics: Man, age 48,
Location: Phone (MA)
Audio file: DM100025_1

- Shown 7 Displays: Snoop, TV, Body, Activity, Performance, Years, Family
- Feels he already knows a lot of this information for himself
- Thought tracking would add too much structure to his life, would rather only track if a problem arose

Work: Writer
Technologies: Cell phone, digital camera, PDA
Devices: odometer on bicycle

[What I monitor] (0:02:25)
CURRENTLY TRACKING: SPENDING BY CATEGORY :[L9]
He keeps a budget and keeps a record of his spending by paying for most things by check. He allocates money for categories such as mortgage, insurance, travel, eating out and clothes. If he spends too much in a category during a month, he cuts back the following month in that category others.

He was doing a weight-lifting program and was tracking that.

[Snoop](0:06:22)
He verified with me that the "detective" would forget all the information after reporting it to him.

POSITIVE REACTION: POSTURE :[L9]
POSITIVE REACTION: OPPORTUNITIES FOR EXERCISE :[L9]
He was interested in the posture and opportunities for exercise.

NEGATIVE REACTION: EATING BEHAVIOR :[L9]
CONCERN: I CAN TAKE CARE OF MYSELF, DON'T TELL ME WHAT TO DO (EATING) :[L9]
"I don't want anyone telling me how to eat. I eat pretty healthy and can basically take care of myself." :[L9]

NEGATIVE REACTION: SOCIAL AWARENESS :[L9]
CONCERN: NAGGING :[L9]
"Social awareness is kind of nagging to me. Repeated use of "you know" in conversation sounds like someone nagging to me." :[L9]

POSITIVE REACTION: WORK HABITS, EFFICIENCY :[L9]
"Work habits, yeah that would help, I'm always looking for ways to be more efficient." :[L9]

IDEA RELATIONSHIP: STRESS, PRODUCTIVITY, PROCRASTINATION :[L9]
I asked him to imagine that they observe him for the next few weeks and tell me what they might notice. He feels he is pretty organized, but notes that he does procrastinate occasionally. He thinks they might be able to recognize ways that he can "get more done with less stress."

"Anything that can help me get work done at the end of the day so I can go home and not think about it, that I would really appreciate." :[L9]

[TV](0:09:00)

NEUTRAL REACTION: TV WATCHING, FEELS HIS TV WATCHING IS REASONABLE :[L9]

He does not currently watch TV, except for one movie a week, and in the past watched very little. I asked him if there was something like watching TV, that he would like to better understand how much time he is spending doing it. He couldn't think of anything.

NEUTRAL REACTION: MOOD, ALREADY UNDERSTAND :[L9]

I pointed out the mood rating and asked him if there was anything he would like to track with respect to how it may affect his mood.

"I don't think there is anything I don't know. I feel better if I get outside. If I get cooped up inside, I get blue." :[L9]

He feels he can recognize when these situations occur.

"I don't really need to be told that." :[L9]

[Body](0:10:45)

POSITIVE REACTION: MUSCLE TONE :[L9]

POSITIVE REACTION: ACTIVITY :[L9]

IDEA USE: COMBINED WITH SUGGESTIONS FOR CORRECTING PROBLEM (BODY) :[L9]

He found all of these examples useful.

"Especially if it found problems, like with muscle tone, and it gave me suggestions for correcting it." :[L9]

POSITIVE REACTION: EXERCISE :[L9]

IDEA USE: WHETHER MEETING GOALS, *NOT* WHAT COULD HAVE BEEN DONE (EXERCISE) :[L9]

He liked the exercise example here more than the "opportunities for exercise" in the snoop example. He felt that just by telling him whether he was meeting goals that he had set, this one was more helpful and less nagging.

He could imagine setting goals and thought that he might check this kind of information before and after he worked out.

POSITIVE REACTION: SKIN CHANGES :[L9]

CONCERN: DON'T HAVE EXPERTISE TO INTERPRET (SKIN CHANGES) :[L9]

IDEA USE: JUST ALERT HIM IF NEED TO SEE A DOCTOR (SKIN) :[L9]

POSITIVE REACTION: HEALING TIME :[L9]

He doesn't feel like he has the expertise to interpret the skin record and would only want it to tell him when there was a potential problem and he needed to see a doctor. He thought healing time might be useful however.

POSITIVE REACTION: ACHES AND PAINS, FREQUENCY :[L9]

IDEA RELATIONSHIP: NECK AND KNEE PAIN AND HUMIDITY/TEMPERATURE :[L9]

CONCERN: INFORMATION MUST BE INCLUDED ABOUT HOW TO FURTHER PREVENT PROBLEM (PAIN AND WEATHER) :[L9]

He has plenty of aches and pains stemming from old injuries and overuse. It would be helpful to see frequency. He believes his neck and knee hurt more when it is damp and cold out. When I asked him if he would like to see frequency of pain in the context of weather changes, he responded "only if it gave me a way of preventing it."

[Activity](0:16:30)

POSITIVE REACTION: SITTING VERSUS MOVING AROUND :[L9]

He would like to know how much time he had spent sitting versus moving around.

CONCERN: WANT REMINDER TO DO SOMETHING MORE THAN INFORMATION ABOUT PAST PERFORMANCE :[L9]

IDEA USE: REMINDER TO STRETCH :[L9]

"What would be helpful for me, I tend to get neck and shoulder pain, I think from hunching over the computer, and if I had a reminder now and then to get up and stretch, that would be useful because I get wrapped up into work. Those kind of activity reminders would be helpful." :[L9]

I asked him about the timescale.

IDEA USE: MULTIPLE TIME SCALES; BY WEEK FOR ACTIVITY LEVELS, BY DAY FOR STRETCHING :[L9]

"I would imagine that I would want to look at them at different times. Like if I wanted an overall idea of how active I'm being, I would look at the week, if I wanted to look at and see how much time I'm sitting, whether I'm getting up and stretching, I would look at the day." :[L9]

[Performance](0:19:12)

He laughed at the idea of tracking solitaire performance.

POSITIVE REACTION: GRIP STRENGTH :[L9]

POSITIVE REACTION: STRENGTH TRAINING :[L9]

IDEA RELATIONSHIP: BALL EXERCISES AND GRIP STRENGTH :[L9]

IDEA USE: RECOVERING FROM AN INJURY :[L9]

He liked at grip strength and strength training. He's working on grip strength following a tennis injury and is supposed to be doing ball exercises, but forgets. He thinks it would be helpful to see the effect of the exercises.

"Trying to recover from an injury, that kind of information very valuable." :[L9]

IDEA USE: AWARENESS OF INJURY HISTORY WHEN PROVIDING INTERPRETATION FOR DATA :[L9]

He would like the system to customize recommendations based on his injury history.

IDEA VARIABLE: PROGRESS ON MULTIPLE WORK PROJECTS :[L9]

He couldn't think of anything that he would track in terms of projects. His work is so involving that he tries not to do any projects outside of work. He uses lists to track his progress at work, particularly for managing multiple projects. He would like to have something that would help him keep track of these projects, as he can be working on up to 10 at a time.

NEUTRAL REACTION: GAMES, DOESN'T PLAY :[L9]

He doesn't play games, so game performance wasn't of interest.

NEGATIVE REACTION: MEMORY :[L9]

CONCERN: MEMORY SCORES WOULD BE DEPRESSING :[L9]

"Memory tests, that would be kind of – well… my memory isn't what it used to be and I'm not sure I'd want to know. I would like ways to improve my memory." :[L9]

He asked what attention tests were. He believes he is aware of his attention performance, but would take suggestions on how to improve it.

IDEA USE: TRACKING TO SEE IF INTERVENTIONS ARE HAVING POSITIVE EFFECT ON MEMORY OR ATTENTION :[L9]

He would only want to monitor mental performance if he was tracking whether particular interventions were having an effect.

IDEA RELATIONSHIP: ACTIVITY AND ATTENTION SPAN :[L9]

IDEA USE: FINDING OPTIMAL AMOUNT OF ACTIVITY FOR MENTAL SHARPNESS :[L9]

He agrees that mental abilities may be correlated with activity and other aspects of routine and behavior.

“If you could correlate memory with say, the amount of activity you did in a week, to tell you that this is the optimal amount of activity if you want to have the best attention span, that’s the kind of thing I was talking about, a suggestion for improvement.” :[L9]

[Years](0:26:00)

NEUTRAL REACTION: COOKING TIME, WOULDN’T BE USEFUL :[L9]

He didn’t think cooking time would be useful and couldn’t immediately think of anything that he would want to track over a long time period.

NEUTRAL REACTION: COMPARING MEMORY TO FRIEND, NOT VALUABLE :[L9]

I pointed out the short-term memory and asked him about the idea of comparing it to a friend.

“It would be fun fodder for conversation, but no [not really valuable].” :[L9]

I ask him about using data over a long time period to evaluate the direction his life is taking and whether he is spending time on what is important to him. He can’t think of anything that he would need to track that he is not already aware of.

“I know how much time I’m spending having fun. I don’t need the minutes tracked.” :[L9]

CONCERN: A COMPUTER DOESN’T KNOW BETTER THAN ME :[L9]

NEUTRAL REACTION: TRACKING FOR SIGNALS OF MAJOR HEALTH SHIFTS, ALREADY KNOW :[L9]

I ask him if there is anything in his life that would be a good warning sign of depression or significant health change. He thought that amount of sleep would be indicative.

“I don’t need a computer to tell me [I have a sense of major shifts]” :[L9]

[Family] (0:30:00)

CONCERN: DATA AS AMMUNITION FOR CRITICISM AMONG FAMILY MEMBERS :[L9]

“Some people could use this as ammunition to criticize each other. That seems to be a potential source of conflict more than anything else.” :[L9]

NEUTRAL REACTION: SCHEDULE, ONLY TWO PEOPLE SO NO NEED :[L9]

I asked him if that applied to his family. There are just two people in his household, just him and his son, so he doesn’t believe there is a lot to track.

NEUTRAL REACTION: WATER USAGE :[L9]

CONCERN: BETTER TO HAVE AUTOMATED APPLIANCES THAN INFORMATION (WATER USAGE) :[L9]

He didn’t want information about water usage. He would rather have appliances that know when to do jobs (wash dishes, water the lawn) when water was least expensive. He believes that knowing how to not waste water is common sense.

NEUTRAL REACTION: TIME SPENT WITH FAMILY, ALREADY KNOW :[L9]

He believes he has a good sense of how much time he is spending with his family.

NEUTRAL REACTION: READING TIME :[L9]

NEUTRAL REACTION: GROUP ACTIVITY LEVELS :[L9]

He has no interest in the reading time or group activity levels.

POSITIVE REACTION: CORRESPONDENCE, REMINDER TO CONTACT :[L9]

CONCERN: NOT LETTING DRAW OWN CONCLUSIONS, JUDGMENTAL :[L9]

“Yeah that [correspondence] would be useful. Sometimes you forget to call someone. If it’s information only just I see. I don’t want someone else saying oh you didn’t call your mother.” :[L9]

CONCERN: PRIVACY (CORRESPONDENCE) :[L9]

He emphasizes that privacy is very important to him.

IDEA USE: SHARE EXERCISE DATA WITH DOCTOR :[L9]

IDEA USE: EXPLICIT DECISION ON WHAT TO SHARE WITH DOCTOR :[L9]

“The exercise stuff, yeah I would want to take that to the doctor. But I would want to share it with the doctor, my choice.” :[L9]

CONCERN: ENFORCING FEELING OF STRUCTURE ON FREE TIME :[L9]

“My day is very structured. I plan it. When I go home I don’t want to track everything. I do that at work. Maybe a little too much for my personal life.” :[L9]

[Sorting] (0:35:30)

He wants a programmable thermostat.
Doesn’t drink

CONCERN: ABILITY DOESN’T MATTER IF ALREADY HAVE SOMETHING THAT DOES IT FOR YOU (AWARENESS OF TIME/WATCH) :[L9]

He knows the time, he has a watch
Goes to the bathroom when he needs to – if he seems to be there all the time, he would know that.
Feels blood sugar and blood pressure could indicate a problem.
He wears what he wants to wear.
He knows he drinks too much coffee, but it may be useful to be reminded of that.
He’s aware of commitments.
Knows about community traffic congestion.
There’s not a lot of competition for air-time in his family (conversation turn-taking)
Feels his email program provides enough
Only tracking foot steps if he has a goal
He’s not very gullible
Just uses computer when he needs them
Wanted to know how knowledge about family would be monitored.
Doesn’t crack knuckles.
Don’t need to track laughing/mood rating
Doesn’t have a pet
Posture – hunch over computer all day
Doesn’t smoking
Doesn’t worry about snacking
Doesn’t think he snores
Doesn’t have a spouse
Understanding more about sleep patterns and how it affects mood

Resorts according to stress

IDEA USE: EXPLICIT INVESTIGATIONS INSTEAD OF BACKGROUND TRACKING :[L9]

There were items that he chose under the stress category but didn’t originally choose. I asked him about this and he said that he would want to track them for a problem area, but wouldn’t want to be constantly reminded of them. He wanted to make an explicit decision when to track those things (e.g., I want to concentrate on affecting stress now, I’ll begin tracking these variables).

ID: [L10]
Date: 8/27/04, 3:30 – 4:30 (75 minutes)
Participant type: Word of mouth
Demographics: Woman, age 52,
Location: Phone (from Florida)
Audio file: DM100027

- Shown 8 Displays: Journal, Snoop, Weight, Place, Body, TV, Daily Rhythm, Social

Work: Retired, married with adult children, recently experienced Hurricane Charley
Technologies: No cell phone, digital camera
Monitoring devices: bathroom scale

[What I monitor] (0:02:30)
CURRENTLY MONITORING: CHOLESTEROL (DOCTOR IS) :[L10]
Her doctor is monitoring her cholesterol (every four months), but she does not have the values.

She doesn't keep a journal.

CURRENTLY TRACKING: APPOINTMENTS IN CALENDARS, WHICH ARE KEPT :[L10]
She writes down appointments in a calendar and keeps the calendars.

[Journal](0:04:45)
POSITIVE REACTION: NEWS/MEDIA READ :[L10]
CURRENTLY TRACKING: MOVIES SHE WANTS TO WATCH :[L10]
Every year she writes down movie titles while watching the Oscars to remind her to rent those movies when next at the video store. She does not keep information about her reaction to the movie or when she watched it, however.

CURRENTLY TRACKING: BOOKS READ AND REACTION :[L10]
BENEFIT: REMINDING SELF ABOUT WHETHER READ SOMETHING, RESPONSE TO THAT SOMETHING (BOOKS READ) :[L10]
She reads a lot and used to keep a record of the books she read, including title, author, when she read it, and a summary. This was helpful to her because she would often forget details about the books she read, but the process was too time-consuming to maintain (she last kept a record of reading 3 years ago).

CURRENTLY TRACKING: TRAVEL LOG :[L10]
BENEFIT: PROVIDING HELPFUL INFORMATION FOR OTHER PEOPLE (PLACES VISITED) :[L10]
NEGATIVE REACTION: PLACES VISITED LOCAL – NOTHING OF INTEREST :[L10]
She has kept a journal on vacations about where she went, what she ate, where she stayed, what she liked about it. This journal has come in handy because she has been able to give information to friends who were planning trips. She can't imagine keeping a similar journal for local places she visits though ("there's nothing great about Orlando").

NEUTRAL REACTION: PEOPLE ENCOUNTERED – WOULD ONLY TRACK SERVICE PROFESSIONALS FOR REFERENCE PURPOSES :[L10]

The only reason she would keep track of people encountered would be if she wanted to keep a list of reference information for plumbers, repair people, or other professionals.

NEUTRAL REACTION: SNACKING – ALREADY KNOW :[L10]
NEUTRAL REACTION: WATER DRINKING – ALREADY KNOW :[L10]
She's on a diet and drinks water at the gym, so she is already conscious of what she is eating/drinking and doesn't see the need for a food journal.

[Snoop](0:10:20)
POSITIVE REACTION: SNOOP :[L10]
NEUTRAL REACTION: EATING BEHAVIORS – UNDER CONTROL WITH DIET :[L10]
Her initial response was that she thought it would be nice to get a report like this. As she looked at the specific examples, she noted that eating behaviors was "pretty much under control" for her now that she was on a low-carb diet.

POSITIVE REACTION: POSTURE :[L10]
She liked posture.

NEUTRAL REACTION: OPPORTUNITIES FOR EXERCISE – UNDER CONTROL WITH TRAINER AND BELONGING TO GYM :[L10]
She already has multiple opportunities for exercise; she belongs to a gym, has a trainer, and does yoga, spinning, and other classes.

NEUTRAL REACTION: SOCIAL AWARENESS – ALREADY A PEOPLE PERSON, DOESN'T NEED HELP :[L10]
She thinks of herself as a "people person" and doesn't feel she needs help with social awareness.

NEUTRAL REACTION: WORK HABITS, NOT ENOUGH STRUCTURE, COORDINATION WITH PEOPLE TO NEED :[L10]
She initially noted that the work habits doesn't apply to her because she does not work outside of the home. When asked about projects inside the home, she indicated that she follows a random schedule and does not need to coordinate with other people.

IDEA USE: MEDICATION REMINDER :[L10]
She has medications and vitamins and forgets to take them. She noted that calcium in particular is challenging because she has to take it 3 times a day. So a medicine reminder would be helpful for her.

[Place](0:15:20)
NEUTRAL REACTION: MISSED MEDICATION, BELIEF THAT REAL PROBLEM IS NOT CONSISTENT, BUT INSTEAD HAS TO DO WITH MEMORY AND TRANSIENT DISTRACTIONS :[L10]
CONCERN: INFORMATION WOULD NOT BE HELPFUL FOR SOLVING PROBLEM; PROBLEM RELATED TO TRANSIENT DISTRACTIONS AND LACK OF DISCIPLINE (MEDICATION TAKING) :[L10]
Following her mention of medication, we took a look at the medication example. She noted that she thinks the problem she has with remembering is that she will get distracted from taking her pills and then an hour later see that she still hasn't taken them yet.

She thinks she could make a better effort into being more disciplined, but describes herself as someone who isn't very structured. She notes that not taking the vitamins is not threatening, so it's easier to slack off.

POSITIVE REACTION: HIGH POINT, LOW POINT AND SITUATIONAL FACTORS (MUSIC, LIGHTING, TEMPERATURE) :[L10]

BENEFIT: IDENTIFICATION OF SITUATIONAL VARIABLES THAT AFFECT MOOD WITHOUT PERSON BEING AWARE (HIGH POINT/LOW POINT) :[L10]
We took a look at high point/low point. She could see it being helpful, because for her there are situation variables that affect her mood, such as music. She thought there may be some things that affect her mood that she is not aware of – for example, she had never considered whether lighting affects her.

IDEA RELATIONSHIP: ROOM TEMPERATURE AND MOOD :[L10]
BENEFIT: UNDERSTANDING MOOD SWINGS :[L10]
They were without power for 6 days following the hurricane and she realized how much she is affected by heat, becoming very grumpy without air conditioning. She thinks having information about what affects you could help you understand mood swings.

NEGATIVE REACTION: ACTIVITY SEQUENCES (UNSURE OF USE) :[L10]
CONCERN: UNFAMILIAR TYPE OF DATA (ACTIVITY SEQUENCES) :[L10]
CONCERN: INSUFFICIENT INTERACTION WITH OBJECTS TO LEAVE INTERESTING PATTERN (ACTIVITY SEQUENCES) :[L10]
She thought the activity sequences data was unfamiliar and had difficulty thinking of an application for that information. She didn't feel that she interacted with objects enough to leave much of an activity pattern.

[Weight](0:25:35) – participant choice
CURRENTLY MONITORING: WEIGHT, UNDER THRESHHOLD :[L10]
BENEFIT: TRIGGER BEHAVIOR CORRECTION, IF GOES ABOVE THRESHHOLD (WEIGHT), DO EXTRA ACTIVITY (EXERCISE) :[L10]
She has become very interested in weight loss, diet, and exercise, so this example is more relevant to her. She weighs herself in the morning and makes sure it is under a certain amount. If it it's not, she exercises harder and more. She'll take an extra kick-boxing course or spinning course.

NEUTRAL REACTION: PHOTOS OF GOOD DAYS, BAD DAYS (WEIGHT) – LITTLE CHANGE :[L10]
She didn't think photos of her body would change from day to day. She didn't think that photos of what she was doing would be very helpful.

NEUTRAL REACTION: WEIGHT WITH GOOD DAYS/BAD DAYS, ALREADY KNOW :[L10]
She thought she understood why there are days when she gets less activity and noted that there are some days when she deliberately gives herself a rest.

[Body](0:30:10)
POSITIVE REACTION: ACHES AND PAINS
BENEFIT: TRACKING DOWN SOURCE OF PAIN RELATED TO EXERCISE :[L10]
CONCERN: BECOMING TOO FOCUSED ON ACHES AND PAINS, BECOMING COMPLAINER :[L10]
She was attracted to the aches and pains example. She talks with her trainer about aches that she may experience to try to determine what exercise could have caused it, but she is not very helpful. She thought keeping a record over time could therefore be helpful. She was concerned however that she may become too focuses on aches and pains, like her father, and begin complaining too much (she notes though that he was more sedentary than her).

POSITIVE REACTION: HISTORY OF EXERCISE :[L10]
IDEA INVESTIGATION: EXERCISE, WHAT EXERCISE IS INTENDED TO ADDRESS, ENJOYMENT OF EXERCISE, WHAT EXCERSIZE NEW ACTIVITY IS REPLACING OVER TIME :[L10]

She thought the history of exercise would be helpful, particularly for understanding limitations. She would like to have a record of the exercises she does, when she does it, what it addresses, and how well she likes it. She recounts how she used to run in her 30s, but has shifted to fast walking at most to preserve her knees, so the limitations reference seems to indicate that she would like to make sure she is balancing her exercise regimen while gradually replacing exercises as she gets older.

CONCERN: PACING OF EXERCISE COURSE ALREADY PROVIDES INFORMATION ABOUT PROGRESS :[L10]
She feels she has an indication of how she's doing with yoga as her instructor introduces more strenuous poses.

[TV](0:36:20)
NEUTRAL REACTION: TIME SPENT OUTSIDE, ALREADY DOING ENOUGH :[L10]
She notes that her TV watching varies a lot based on what's airing, being an avid sports fan. She believes she spends sufficient amount of time outside. She notes that she watches TV in the background while reading sometimes, particularly as she waits for the news to come on.

POSITIVE REACTION: TV WATCHING :[L10]
IDEA RELATIONSHIP: TV WATCHING AND BEING INFORMED :[L10]
BENEFIT: BETTER UNDERSTANDING OF BEHAVIORS THAT HAVE BECOME HABITS (TV WATCHING) THAT ARE COMBINED WITH OTHER ACTIVITIES AND THEREFORE DIFFICULT TO PERSONALLY CALCULATE :[L10]
BENEFIT: CHANGE IN BEHAVIOR (TV WATCHING) :[L10]
She would like to know how much time she's watching TV. Having been without TV for 6 days, she's become aware of how much she depends on TV for staying informed. She also notes that TV is a habit for her, something she starts in the morning and combines with other activities. She thinks she might modify her habits if she had a better understanding of how she's watching it.

IDEA INVESTIGATION: PRODUCTIVITY AND TIME MANAGEMENT :[L10]

> It would be neat to know where you spend her time. I might waste time piddling around and then go, what did I do today? I don't' know, but another day's gone <laughs> So that would be really interesting to break it down and organize my time better. :[L10]

IDEA VARIABLE: POSTPONED ACTIVITIES :[L10]
She would like to know the activities and whether she was spending too much time in one area. She would like to track if there were things she was putting off.

[Daily Rhythm](0:43:30)
POSITIVE REACTION: DAILY RHYTHM :[L10]
BENEFIT: INFORMATION ABOUT ACTIVITY PATTERNS CAN PROVIDE COUNTERBALANCE OF STRUCTURE WHEN YOU ARE A LESS SCHEDULED TYPE OF PERSON :[L10]
She liked this example a lot. She notes that she is a spontaneous person who isn't anal or scheduled, so having this information might provide a counterbalance. She knows about herself that she doesn't like to be doing any one thing for a very long period, but instead shifts activities often and not according to a set plan.

IDEA INVESTIGATION: WAKING UP ROUTINE TO BETTER MANAGE TIME AND GET STARTED WITH DAY FASTER :[L10]
She's not a morning person, but wakes up at 6am. She has to drink coffee to wake herself up, particularly on days when she needs to go to the gym. She would like a better understanding of her waking up routines to better manage her time and get going faster.

POSITIVE REACTION: COMMITMENTS :[L10]

BENEFIT: MOTIVATION TO STAY ON TASK :[L10]
IDEA USE: SOME CHOICE COMBINED WITH COMMITMENT OR STRONG PERSUASION :[L10]
She feels she does know what she's procrastinating on, but would like something like the commitments example to provide motivation and get her on track for attending to tasks she's putting off. She would like something that would give her some flexibility ("choose one thing on the list"), but would commit her to acting ("do it now").

[Social](0:48:00)
NEUTRAL REACTION: SOCIAL, DOESN'T TALK ON PHONE OR EMAIL MUCH :[L10]
She doesn't like to talk on the phone very much and instead tries to meet with friends in person. She does talk with her dad on the phone and via email.

POSITIVE REACTION: ACCUMULATED TIME SPENT ON PHONE TO REASSURE COMMUNICATING ENOUGH WITH FAMILY :[L10]
BENEFIT: DATA OVER TIME CAN REASSURE THAT OVERALL AMOUNT OF COMMUNICATION IS SUFFICIENT, GIVEN THAT THERE ARE UPS AND DOWNS IN FREQUENCY :[L10]
She feels that having information about the accumulation of time would be helpful. She knows most of it. She thought that if there was a way to show that the total amount of time she is committing to communicating with her dad, over a period of months, was meeting some goal, that that might make her feel better, because there is a lot of variability (she's calling him a lot right now) and it might balance out the times when is in little communication with him.

[Sorting] (0:52:15)
BENEFIT: HAVING DATA TO ANSWER QUESTIONS DURING CLINICAL VISITS (HOW MUCH ALCOHOL DRINK EACH WEEK) :[L10]
She says yes to alcohol drinking because she notes that they usually ask at the doctor's office how much you drink per week.

She thought about barometric pressure when she felt her ears popped when the hurricane came through.

IDEA USE: TRACK SOME THINGS OF DEVELOP CHRONIC DISEASE WHEN OLDER (BLOOD PRESSURE, BLOOD GLUCOSE, TRIPS TO THE BATHROOM) :[L10]
She notes that some things might change as she gets older – noting her dad would be more interested in the health issues. She thought she would want to begin tracking blood pressure, blood glucose, or trips to the bathroom if she developed a chronic disease.

IDEA VARIABLE: CHOLESTEROL :[L10]
IDEA USE: KEEPING TRACK OF HEALTH VALUES (CHOLESTEROL) ON COMPUTER RATHER THAN FROM CLINICAL VISITS :[L10]
BENEFIT: NOT HAVING TO RELY ON A DOCTOR TO MONITOR HEALTH VALUES (CHOLESTEROL) :[L10]
She notes that she would like to have more information about her cholesterol as she ages, rather than relying on a doctor to track it. In general, she would prefer to track health values herself, on her computer, rather than relying on a doctor.

IDEA USE: CLOTHING CHOICES TO MAKE BETTER PURCHASING CHOICES :[L10]
She would like to track clothing choices in order to make better purchasing choices.

She thinks she is gullible and often purchases things just to avoid confrontation. She has gotten better, she thinks, but would like to monitor it.

Concern: if something is already bad, no need to track (night vision) :[L10]
She knows that she doesn't have good night vision, so doesn't think she needs to track it.

She thinks she would track pet activity if her dog were younger and more active.

She doesn't do sports.

She resorts by "stress"

Idea variable: which conversations are arguments :[L10]
She thought that if conversation turn taking had information about whether the conversation was argument, it might be helpful.

Idea relationship: loud music and stress :[L10]
She selected hearing because loud music drives her crazy

Idea relationship: variation from routine and feelings of disruption :[L10]
She noted that experiencing the hurricane showed her how much variation from routine can be disruptive.

ID: [L11]

Date: 8/23/04, 2:00 – 3:00 (55 minutes)
Participant type: Word of mouth
Demographics: Woman, 53 (1951), works in the health-technology field
Location: Phone (MA)
Audio file: DM100017

- Shown 8 Displays: Social, Performance, Email, TV, Body, Weight, Family, Years
- Particularly interested in activity-based metrics

Work: Manager of home health care program
Technologies: Cell phone, digital camera, pda
Devices: scale, pedometer (got it at Xmas, doesn't use), blood pressure device for wrist

[What I monitor] (0:02:25)
CURRENTLY MONITORING: WEIGHT :[L11]
CURRENTLY MONITORING: BLOOD PRESSURE (OCCASIONALLY) :[L11]
She tracks her weight on a daily basis and occasionally checks blood pressure.

She wrote down her weight for a couple of weeks last year, but eventually that got lost and she stopped.

She doesn't keep a journal.

[Social](0:04:55)
POSITIVE REACTION: SOCIAL :[L11]
She thought that the network was representative in that she tends to call family more than anyone else. She does more work phone calling than represented.

BENEFIT: INTERESTING, FOR CURIOSITY SAKE (SOCIAL PHONE) :[L11]
She doesn't think that this information would be helpful, but interesting.
IDEA INVESTIGATION: PHONE USE AND WORK AS JOB POSITION CHANGES :[L11]
It would be interesting to track as the years go by with work, does it increase, does it decrease, how does that correspond to the particular positions you have. :[L11]

IDEA VARIABLE: ALL FORMS OF COMMUNICATION, ELECTRONIC, BLACKBERRY, TELEPHONE, RELATED TO WORK :[L11]
Any kind of connection – electronic, blackberry, telephone – for work would be interesting to her.

She thinks that social would vary quite a bit with regard to who her friends are at the particular time and her work situation. She noted that some relationships stay constant – those who are closer to her.

NEUTRAL REACTION: SOCIAL – PHONE ON SHORT TERM, PHONE BILL ALREADY DOES THIS :[L11]
Her phone bill has the breakdown about calls and so she doesn't feel she needs additional information on a short-term basis.

IDEA USE: DATA THAT YOU OCCASIONALLY SCRUTINIZE, LIKE PHONE BILLS AND CREDIT CARD BILLS :[L11]
It's like your credit card bill. Sometimes you really analyze it, where am I spending my money, but probably not on a regular basis. :[L11]

POSITIVE REACTION: GOOD TIMES TO CALL FOR CALLS WITH FRIENDS :[L11]
NEUTRAL REACTION: GOOD TIMES TO CALL FOR CALLS WITH FAMILY, ALREADY KNOW :[L11]
She thought that the good time to call example would be good for conversations with friends, but felt that she knows her family so well, she wouldn't even look at the information ("because you know that intrinsically, when to call").

POSITIVE REACTION: USING CALL INFORMATION TO INVESTIGATE HEALTH OF A RELATIONSHIP OVER TIME – INTERESTING, BUT NOT NECESSARY :[L11]
When questioned, she initially thought she would know about the health of a relationship with a friend and that his information wouldn't be needed, but on consideration, thought it might be interesting to "look back on the cluster of calls, what that meant," but didn't think it would be necessary.

[Performance](0:10:35)
POSITIVE REACTION: PERFORMANCE, SPORTS, GRIP STRENGTH, AND STRENGTH TRAINNG :[L11]
IDEA INVESTIGATION: PERFORMANCE FOLLOWING REST PERIODS OR SLACKING FROM EXERCISE REGIMENS :[L11]
She had an immediate positive response to this example and indicated that sports, grip strength, and strength training were of particular personal interest to her. She does strength training and sports and would like to see improvement and how periods of rest affect her performance (where does she start back on the chart after a period of slacking off).

IDEA USE: DATA AUTOMATICALLY COLLECTED AND CORRELATED WITH HEALTH STATUS VARIABLES (AS OPPOSED TO TODAY'S PEDOMETERS, WHICH REQUIRE MANUAL RECORDING) :[L11]
She does aerobic activity, strength training, and walking. She has a pedometer, but she often forgets about it, it's not a natural thing to put on, and even when she has the information, she doesn't know what to do with it. She would like it integrated and correlated with status of health and well-being.

> Telephone calls are interesting to me. This [physical performance] is important. :[L11]

IDEA RELATIONSHIP: PHYSICAL PERFORMANCE AND WEIGHT :[L11]
IDEA VARIABLE: "HEALTH STATUS" SCORE REPRESENTING COMBINED METRICS :[L11]
IDEA USE: "HEALTH STATUS" ALGORITHM BASED ON STUDIES THAT HAVE BEEN DONE, RATHER THAN PERSONAL EVALUATION :[L11]
It would be helpful to know that her performance and strength were increasing, that she could go further, faster. She would like to see the information integrated with weight. She would like a combined "health status" score to look at, rather than just having it broken down on all these measures. She would like the algorithm for health status to be determined based on studies that have been done.

> I think I may know, but is that the best algorithm? Probably not. :[L11]

BENEFIT: NO CHANGE (IN PERFORMANCE) TRIGGERING ALTERATIONS TO WORKOUT OR DIET :[L11]
If she didn't see change, she would make alterations to her workout or diet.

POSITIVE REACTION: MENTAL PERFORMANCE, BUT NOT AS IMPORTANT AS PHYSICAL PERFORMANCE :[L11]
BENEFIT: MENTAL PERFORMANCE WILL LIKELY BECOME MORE IMPORTANT AS AGE :[L11]
With regard to the mental performance measures,

> I guess – important, interesting. Wouldn't be first on my list, but I guess the older I get, the more I would want to track that. :[L11]

CONCERN: IF WORKING, ALREADY CAN JUDGE WHEN AND HOW ATTENTION AND MEMORY ARE IMPAIRED :[L11]

Because she works every day, she believes she is a good judge of her attention and memory and knows when she is having an off day.

```
 I think memory and attention, I think you know that pretty well yourself. :[L11]
```

IDEA USE: DON'T COMBINE PHYSICAL PERFORMANCE AND MENTAL PERFORMANCE :[L11]
She would want to see mental performance measures separate from physical measures.

[Email](0:17:45)
POSITIVE REACTION: OUTDOOR CONDITIONS, WEATHER RELATED TO PHYSICAL EXERCISE ROUTINES :[L11]
She immediately reacted to the information related weather to physical exercise, because it is something that directly impacts her exercise regimen. She'll adjust the length of her walk based on temperature. She feels that season affects exercise quite a bit for people.

IDEA USE: INFORMATION DELIVERED (WEATHER), RATHER THAN HAVING TO BE SOUGHT :[L11]
She notes that she organizes her day around the weather, whether to go out to do an errand, what to wear, etc. and would like to have that kind of information at least twice a day. When asked whether the current information available about weather is sufficient, she said it would be nice to receive it and not have go look for it.

POSITIVE REACTION: PAIN IN CONTEXT OF RECOMMENDATIONS :[L11]
POSITIVE REACTION: NEWS RELATED TO CURRENT ACTIVITIES, TIME SPENT IN CAR :[L11]
IDEA VARIABLE: TIME SPENT SITTING :[L11]
BENEFIT: MOTIVATING ACTIVITY (TIME SPENT SITTING) :[L11]
She thought the pain and food examples were good, but particularly reacted to the time spent in car example. She noted that she would like to know how many minutes she spent sitting in the chair today. She thought that would motivate more activity.

IDEA USE: DATA WITH POTENTIAL CONSEQUENCES OR OTHER METRICS (TIME SPENT SITTING WITH RISK OF DIABETES, CALORIES BURNED) :[L11]
She imagined what it would be like to have the data (you sat for 6.5 hours today) with information about the significance of that (you're more prone to diabetes, you burned less calories) – she thought this information would be motivating.

NEGATIVE REACTION: GROUP STATS ABOUT PHYSICAL ACTIVITY OR MOOD (INTRUSIVE) :[L11]
IDEA INVESTIGATION: DEMOGRAPHIC STATS AND GENERAL ACTIVITIES (TIME SPENT WORKING) OF NEIGHBORS :[L11]
BENEFIT: SENSE OF SECURITY TO KNOW MORE ABOUT NEIGHBORS :[L11]
The group data was not very interesting to her as it was. Instead of knowing about their health, she would like to know about the demographics of her neighbors – how many students, break down in professions, how many married, average age. When I suggested knowing how many hours they spend working, she agreed that would be interesting, and commented that today we don't know our neighbors too well, so having information would give a sense of comfort. She wasn't interested in invasive information, such as average mood or physical activity.

[TV](0:25:30)
POSITIVE REACTION: TV WATCHING COMPARED WITH AVERAGE FOR DEMOGRAPHIC :[L11]
POSITIVE REACTION: TV WATCHING, WOULDN'T AFFECT BEHAVIOR, BUT FOR CURIOSITY SAKE :[L11]
She thought it was interesting to compare her TV watching with the average for her demographic. She doesn't spend many hours all at once in front of the TV, but watches it in the background while doing other tasks. She doesn't think it would affect what she would do, but she is curious.

IDEA USE: SOMETHING THAT WOULD HELP HER TAILOR TV WATCHING TO FAVORITE SHOWS ONLY :[L11]
She wishes there was something that would help her tailor her TV watching to only shows she really enjoys and wants to plan on watching.

POSITIVE REACTION: HEART RATE AND MOOD WITH TV WATCHING, CURIOSITY SAKE :[L11]
She thought it would be interesting to see her heart rate and mood while watching TV. She thinks her mood would be in between and her heart rate would be lower than normal. She thinks that there would be differences when watching her favorite shows or new shows. This would again be something she would just be curious about.

[Body](0:30:00)
NEUTRAL REACTION: BODY - SKIN CHANGES :[L11]
POSITIVE REACTION: BODY - ACTIVITY, EXERCISE, MUSCLE TONE :[L11]
POSITIVE REACTION: BODY - ACHES AND PAINS :[L11]
She thought of the body example as a "micro-analysis" and found it interesting. Skin changes was not of interest to her, but activity and muscle tone were really interesting. She thinks aches and pain would be helpful.

[Weight](0:31:00)
POSITIVE REACTION: WEIGHT WITH PEDOMETER STEPS :[L11]
She liked the idea of seeing weight with pedometer steps.

IDEA USE: NO DATA ENTRY, "MINDLESS" TRACKING TOOLS :[L11]
She emphasized that she wanted something that would automatically record and correlate the data rather than requiring data entry. She wanted tracking tools that were "mindless"

POSITIVE REACTION: DATA WITH GOALS :[L11]
POSITIVE REACTION: PHOTOS FOR GOOD DAYS/BAD DAYS :[L11]
She was strongly responsive to the idea of comparing data with goals and thought the photos would be helpful.

[Family](0:33:00)
POSITIVE REACTION: FAMILY SCHEDULE :[L11]
BENEFIT: DATA AS A TUTORIAL :[L11]
BENEFIT: DATA TO GENTLY NUDGE FOR BEHAVIOR CHANGE :[L11]
This is like a tutorial, like a nice gentle way of educating and highlighting what they are doing now, and nudging them along the proper path.

She's very regimented and could imagine that her schedule would look very much the same from day to day.

IDEA RELATIONSHIP: ACTIVITIES FROM PREVIOUS DAY, SLEEP QUALITY, AND FEELINGS OF FATIGUE/OFF-GAME :[L11]
She would like to know how the activities on the previous day and sleep affected how she felt the next day ("draggy").

> Sometimes it takes you a while to discover gee, why you feel that way, and what you can do to modify your behavior. :[L11]

[Years](0:34:30)
POSITIVE REACTION: TIME SPENT COOKING :[L11]

BENEFIT: EVEN IF HAVE A GOOD SENSE OF BEHAVIOR, SEEING DATA CAN HELP IMPROVE :[L11]

She thought the number of minutes spent cooking would be really interesting.

```
I think anything that has to do with behavior is really interesting. I think we all think
 we have a handle on our behavior, but it's helpful to see something like this so you can
 improve. :[L11]
```

POSITIVE REACTION: DATA OVER YEARS :[L11]

BENEFIT: DATA OVER YEARS HELPFUL FOR CHANGES THAT ARE DIFFICULT TO INTUITIVELY BE AWARE OF (MEMORY DECLINE, EFFICIENCY) :[L11]

She liked the idea that it was over several years and felt that it was appropriate for identifying changes that are difficult to personally be aware of.

```
I think as I've gotten older, I am working in a more efficient manner, that could be
 meals, that could be planning what we're going to eat for the week. I didn't have the
 planning down at that time (10 years ago). :[L11]
```

She thought the time spent with friends would be interesting.

POSITIVE REACTION: MEMORY IN CONTEXT OF DECLINE :[L11]

BENEFIT: PROMPT CONVERSATION WITH PHYSICIAN (MEMORY DECLINE) :[L11]

She thought the memory in the context of friends would be good. She thought that decline would prompt her to talk with her physician and noted that changes in memory (over years) are difficult to personally detect. She would want to know even if it showed decline.

IDEA VARIABLE: PHYSICAL ACTIVITY OVER YEARS :[L11]

IDEA VARIABLE: EFFICIENCY, TIME MANAGEMENT, PLANNING OVER TIME TO SEE HOW IMPROVE :[L11]

She would like to track physical activity and mental activity and would like to see how she gets better at time management and planning over time.

NEGATIVE REACTION: SHARING DATA WITH FAMILY MEMBERS (INTRUSIVE) :[L11]

She was reluctant about the idea of sharing information with family members.

```
I'm someone who doesn't want to be intrusive… the only other person would be my
 physician. :[L11]
```

POSITIVE REACTION: SHARING DATA WITH PHYSICIAN :[L11]

She felt that she could share activity or social based information with her physician. Her physician asks about social wellbeing. She couldn't think of anything that was difficult to talk about or to summarize when talking with her physician.

[Sorting] (0:41:35)

She noted that she is poor at recalling jokes and stories, but believes she has always been that way, and would like to track it.

IDEA INVESTIGATION: CLOTHING CHOICES TO IDENTIFY "GETTING IN A RUT" :[L11]

She would like to know when she is getting into a rut in wearing the same thing.

She reacted strongly (positive) to conversation turn-taking, gullibility, hormone cycles, knowledge about friends and family (she thinks she would be surprised), laughing, mood self-rating, physical exercise concepts, posture, response time, time spent with friends, use of space, variation by routine.

CONCERN: KNOWING HOW MUCH MULTI-TASK MAY LABEL PERSON AS TOO STRUCTURED OR ANAL :[L11]
She multi-tasks all the time and doesn't want to know to what extent, because it would "show people" how anal she is.

BENEFIT: TRACKING CONSTRUCTS THAT ARE DIFFICULT TO INTUITIVELY MEASURE (NIGHT VISION) :[L11]
She notes that night vision is important and that it is difficult to realize how much it is diminishing.

NEGATIVE REACTION: SNACKING, DISCOURAGING :[L11]
CONCERN: FORCES YOU TO ADDRESS BEHAVIOR THAT YOU MAY NOT BE DISCLIPLINED ABOUT (SNACKING) :[L11]
POSITIVE REACTION: SNACKING :[L11]
BENEFIT: GETTING CONTROL OF BEHAVIOR :[L11]
CURRENTLY TRACKING: (PLANNING TO) FOOD JOURNAL :[L11]
Tracking snacking would make her feel bad, but she is planning on keeping a food journal. She doesn't feel that she has a weight problem, but doesn't want to have one. She's heard that a food journal is the best way to take control of her eating, but feels that she will only be able to maintain it for a few weeks.

IDEA USE: SKIN CHANGES ONLY IF MONITORING A MOLE :[L11]
She would only want to track skin changes if monitoring a mole.

She resorts by physical activity.

She chooses refrigerator openings, idle time, snacking, and alcohol drinking, though she notes that she didn't choose them before.

IDEA USE: TRACKING WITH A GOAL IN MIND :[L11]
> Initially, I didn't think it was important, but when you think about it in a different context and start to focus and narrow down what you are trying to ascertain, it gives it new meaning. So if I'm looking at physical activity, I want to track my idle time… it helps to highlight what the issue is. :[L11]

IDEA USE: SOME METRICS ON OCCASION (BLOOD SUGAR, BLOOD PRESSURE, COFFEE/TEA DRINKING), OTHERS ALWAYS IN BACKGROUND (GRIP STRENGTH, HORMONE LEVELS, MUSCLE TONE, SPORTS PERFORMANCE) :[L11]
She would like to occasionally track her blood sugar, blood pressure, coffee/tea drinking; but things that are "more pertinent" as she perceives and would want to track all the time, would be grip strength, hormone levels, muscle tone, sports performance.

IDEA USE: DATA MONITORING STATION :[L11]
Her final recommendations would be to make tracking less cumbersome – steps in the day, caloric intake, activity in the day. She would like the data transmitted to a monitoring station and get feedback from a built-in algorithm.
> It's one thing to analyze your own data, and it's another thing to have a professional analyze your data. :[L11]

ID: [L12]

Date: 8/15/04, 9:43-10:54PM (80 minutes)
Participant type: Participant, through word of mouth (mother of alum)
Demographics: Woman, 53 years
Location: Phone
Audio file: DM100010

- Shown 5 Displays: Journal, Snoop, TV, Place, Family
- Interested in eating and exercise tracking
- Concerns about feeling criticized (work habits), but also feels could balance what she hears from other people (social awareness)
- Doubtful that family would go along with tracking

Work: Food prep, sales
Technologies: Cell phone, digital camera, no PDA
Monitoring devices: Scale, just got a pedometer, but doesn't use; no heart rate monitor

`[What I monitor] (0:07:29)`
CURRENTLY MONITORING: WEIGHT :[L12]
CURRENTLY MONITORING: FOODS EATEN :[L12]
She weighs herself and "kind of tracks" what she eats.

CURRENTLY MONITORING: BLOOD PRESSURE :[L12]
CURRENTLY MONITORING: BLOOD GLUCOSE :[L12]
She tracks blood sugar and blood pressure. She had a home blood pressure monitor, but it doesn't work, so she uses the machine at the grocery store. She has a home gluco-meter that she uses. She was recording them for a while, but lately is just making sure they are within a range. The blood sugar machine keeps a record of the data.

IDEA RELATIONSHIP: FRUIT, BLOOD SUGAR, EXERCISE, WEIGHT :[L12]
She has noticed that the less fruit she eats, the more she exercises, and the lower her weight is, the better her blood sugar.

IDEA VARIABLE: MEMORABLE MOMENTS, SELF AND FAMILY :[L12]
She writes down a couple times of year what went on in her and her husband's life and what went on in her kids' lives, but would like to do so more often. She keeps photos.

IDEA USE: LANDMARK-BASED WALKING PROGRESS :[L12]
She walks and knows that a pedometer would be helpful, but monitors her distance instead by walking to particular landmarks. When she walks on a treadmill at the gym, she makes use of the distance tracking technology.

IDEA VARIABLE: TRACKING KIDS' DEVELOPMENT :[L12]
She tracked her kids' height on the door frame as they grew up.

IDEA VARIABLE: PERISHABLE FOODS :[L12]
She writes down when she opens milk or eggs on the calendar.

IDEA VARIABLE: PILLS TAKEN :[L12]

She keeps track of which pills she has taken each day.

IDEA VARIABLE: FILTER (AC) CHANGES :[L12]
She tracks money in her checkbook. She tracks when she changes the filters in her air conditioner.

She tries to drink 8 glasses of water a day, but does not keep a record of it.

She keeps a grocery list and a to-do list.
“I guess I’m a tracker. <laughs>”:[L12]

[Journal] (0:18:29)
POSITIVE REACTION: PLACES VISITED :[L12]
POSITIVE REACTION: PEOPLE ENCOUNTERED :[L12]
IDEA USE: PLACES VISITED TO ADD UP MILEAGE FOR TAXES :[L12]
IDEA USE: SOMETHING NICE ABOUT PEOPLE ENCOUNTERED TO FEEL GOOD :[L12]
She interpreted the displays as representing “some person.” (oh, this person tracks that too).
“You could use it as a diary or a calendar, maybe for your taxes to add up mileage. If you needed to add up your food expenses or something, if you want to budget. You could budget how much you spent over time.” :[L12]

“I would write something nice about the person, a feel good type of thing. That would probably be it.” :[L12]

POSITIVE REACTION (STRONG): SNACKING :[L12]
POSITIVE REACTION (STRONG): WATER DRINKING :[L12]
POSITIVE REACTION: MEMORABLE MOMENTS :[L12]
BENEFIT: RECORD OF YOUR LIFE TO PASS ON TO OTHER PEOPLE :[L12]
She thought that the places visited and people encountered would be helpful. She particularly liked snacking and water drinking.
“The memorable moments would be good because you could save it for years and people could look at what you did.” :[L12]
CURRENTLY TRACKING: WORLD EVENTS ASSOCIATED WITH PERSONAL EVENTS (BIRTHDAYS) :[L12]
She often saves newspapers from birthdays, her own or her childrens’, so she can look back on what was going on at the time. This is how she interpreted the news/media idea.

“I don’t think I would want to track it everyday. I’ll just track birthdays or something major, like when the war started…”:[L12]

POSITIVE REACTION: RECORDING REACTION TO WORLD EVENT :[L12]
I ask her whether she would just record the date or other information, such as her reaction to the event.
“That would be a good idea, to write down your reaction to them, I never thought of that one before, but that would be good.” :[L12]

[Snoop] (0:23:15)
POSITIVE REACTION: EATING BEHAVIORS :[L12]
BENEFIT: IT’S EASIER TO RECOGNIZE MISTAKES WHEN YOU SEE THEM “IN PRINT” (EATING) :[L12]
IDEA VARIABLE: HOW MUCH YOU EAT, FREQUENCY OF EATING :[L12]
“Yeah, like when you’re taking something to eat that you shouldn’t be taking to eat, that wouldn’t be on the diet that you wanted to stay on. It’s easier when you see it in print, how much you eat, whether it’s too much or too often.” :[L12]

POSITIVE REACTION: OPPORTUNITIES FOR EXERCISE :[L12]

IDEA USE: WHETHER ACTIVITIES AND EXERCISE ARE MEETING GOALS :[L12]
BENEFIT: EVALUATION OF CURRENT PERFORMANCE TOWARD GOAL (EXERCISE, EATING) :[L12]

In addition to the eating behaviors, she liked the opportunities for exercise. She thought it would be valuable to have someone tell her whether the work she is doing is helpful to her goals, recommend opportunities for doing more, and give her information that might motivate her to continue.

> "Exercise. If someone could tell you whether what you're doing is helping you lose weight or tone up or giving you some kind of exercise… help me to stay on a better path to eating, showing me where I can fit in more exercise, or maybe being more motivated to exercise, or just showing me whether the exercise I normally do is working." :[L12]

NEUTRAL REACTION: POSTURE :[L12]
IDEA USE: POSTURE (WALKING WITH HEAD UP OR HEAD DOWN) AS INDICATOR OF CONFIDENCE :[L12]

> "I don't know so much about the posture. I guess it would give you an idea of how confident you are, if you are walking with your head up or head down, but that would be the least interesting for me." :[L12]

NEGATIVE REACTION: WORK HABITS, GETTING ENOUGH CRITIQUE FROM EMPLOYER :[L12]

She felt that her boss already tells her enough about her work habits.

> "They tell you do this or do that. Sometimes you feel like a yo-yo." :[L12]

POSITIVE REACTION: SOCIAL AWARENESS :[L12]
BENEFIT: MORE OBJECTIVE MEASURE TO BALANCE OUT SUBJECTIVE I NPUT FROM FAMILY (SOCIAL AWARENESS) :[L12]

She felt the social awareness information would be good to get from a third party.

> "The social awareness might be like your husband nagging you or your kids nagging you… another person would be better, because they are not always objective. It would be nice to have a balance." :[L12]

[TV] (0:30:03)

POSITIVE REACTION: TV WATCHING :[L12]
BENEFIT: ASSISTING WITH TIME BUDGETING (TV) :[L12]

She could imagine that the value in tracking this information, in terms of time budgeting or changing behavior.

PROCEDURE: PARTICIPANT SPECULATES ABOUT HYPOTHETICAL SITUATION (IF I WAS TRYING TO CHANGE…) RATHER THAN CURRENT NEED :[L12]
BENEFIT: EVALUATING TIME SPENT ON ACTIVITIES FOR BEHAVIOR CHNAGE :[L12]
CONCERN: EVALUATING TIME SPENT ON ACTIVITIES GOING HAND IN HAND WITH HAVING A STRICT SCHEDULE :[L12]

> "If I was trying to budget my time better, then I would know how much time I watch TV, or how much time I went outside, or how much time I spoke to my husband. Then yeah, it would be valuable. If I had a goal to stay on a strict schedule, but I'm not really sure…":[L12]

> "If I wanted to cut back on TV, then it would be valuable to know how much time I actually watched it." :[L12]

NEUTRAL REACTION: TIME SPENT OUTSIDE, QUESTIONING USEFULNESS :[L12]
IDEA RELATIONSHIP: TIME SPENT OUTSIDE AND SUN-EXPOSURE (BUT NO INTEREST IN TRACKING) :[L12]

As she thought more about it, she noted that some information would be difficult to apply.

> "I don't know why I would need to know how much time I spent outside, unless I was worried about getting too much sun."

NEUTRAL REACTION: MOOD, ALREADY KNOW WHEN UP OR DOWN :[L12]

> "My mood, I don't think I really need to track it, I know when I'm up or down." :[L12]

CONCERN: MORE DETAIL THAN NEEDED (MOOD TRACKING) :[L12]
"I don't really think I would need to be this detailed. I don't think I would need a tracking device for it." :[L12]

PROCEDURE: NOT RECOGNIZING RELATIONSHIPS BETWEEN VARIABLES :[L12]
NEUTRAL REACTION: FACTORS CONTRIBUTING TO MOOD, ALREADY KNOW IT :[L12]
She didn't immediately notice the relationship between variables, so I pointed out that someone might want to see how TV watching affected other things in their life. I then asked her if there were relationships she wanted to see, for example what affects her mood.
"I don't think I would track it, I would just know it. At this time in my life." :[L12]

POSITIVE REACTION: TIME SPENT EXERCISING :[L12]
I then asked her about whether there was information related to how much she exercised that would be helpful.
"Yeah, that I could see the value in that." :[L12]

POSITIVE REACTION: COMPARING WITH AVERAGE – ENCOURAGING OR MOTIVATING (TIME SPENT EXERCISING) :[L12]
BENEFIT: IF BETTER THAN AVERAGE, MAY BOOST SELF-ESTEEM :[L12]
CONCERN: IF WORSE THAN AVERAGE, MAY BE DISCOURAGING :[L12]
BENEFIT: IF WORSE THAN AVERAGE, MAY MOTIVATE CHANGE :[L12]
I asked her to imagine that instead of minutes watching TV, the chart was showing her minutes spent exercising. I asked her about seeing the average time for people in her age group.
"It might make me feel better if I was exercising more than the average person. It might be discouraging if I was exercising less… but it would probably motivate me to do it more. So yes I guess there would be value in it." :[L12]

PROCEDURE: PARTICIPANT REVERSES DECISION ON WHETHER MOOD COULD BE HELPFUL :[L12]
POSITIVE REACTION: MOOD AND TIME SPENT EXERCISING :[L12]
IDEA RELATIONSHIP: MOOD, WEIGHT, WAISTLINE, AND EXERCISING :[L12]
I asked her whether seeing her mood with time spent exercising would be helpful.
"My mood, my weight, my diet with exercising, yeah definitely. If there was weight lost, inches lost, if I felt better." :[L12]

[Years] (0:36:30)
NEUTRAL REACTION: SHORT-TERM MEMORY OVER YEARS, ALREADY KNOW, WOULD RECOGNIZE CHANGE :[L12]
"I don't think I would need to track my memory. I think I would realize, you know from the things I don't remember that, I kind of know it." :[L12]

NEUTRAL REACTION: TIME SPENT COOKING, ALREADY KNOW SPEND A LOT OF TIME :[L12]
NEUTRAL REACTION: OPENING CABINETS, ALREADY KNOW :[L12]
"I kind of know I spend a lot of time cooking or opening cabinets. I don't think I would bother tracking it." :[L12]

POSITIVE REACTION: COMPARING SHORT-TERM MEMORY WITH FRIENDS :[L12]
BENEFIT: COMPARISON TO UNDERSTAND WHETHER NORMAL :[L12]
The only variable of interest to her was the comparison for the short-term memory score.
"Then you would know if you were normal or not, yeah, I guess it would help." :[L12]

IDEA USE: IF GETTING WORSE (MEMORY) TALK TO DOCTOR, DO PERSONAL RESEARCH ABOUT WHAT CAN BE DONE, TAKE SUPPLEMENTS :[L12]
I asked her what she would do if she saw that her memory was getting worse.

"I might talk to the doctor about it, ask if it was normal or if there was something I could do. Go to lectures to find out what people know about increasing their memory, take some supplements, look at the Internet." :[L12]

BENEFIT: KNOWING EARLY ABOUT CHANGE TO MAKE PLANS FOR HOW TO COMPENSATE :[L12]

"I would definitely want it. You need to make plans to figure out how you would keep remembering things. I would want that information. :[L12]

[Place] (0:43:00)

PROCEDURE: PARTICIPANT HAD TROUBLE INTERPRETING DISPLAY (PLACE) :[L12]

PROCEDURE: PARTICIPANT IS RESPONDING ACCORDING TO HYPOTHETICAL GOAL RATHER THAN ACTUAL GOAL :[L12]

BENEFIT: MATERIAL TO ARGUE FOR NEED FOR CHANGE WITH FAMILY (SEDENTARY ACTIVITY) :[L12]

NEUTRAL REACTION: ACTION SEQUENCES, ONLY IF WANTED TO ARGUE FOR BETTER TIME MANAGEMENT :[L12]

She had difficulty interpreting the floor plan.

"Well it would be helpful if I wanted to point out how we weren't getting anything done except for watching TV and stuff like that. We would know where to direct more of our efforts at doing stuff." :[L12]

PROCEDURE: SOMEWHAT MIXED REACTION (ACTION SEQUENCE IN SPACE) :[L12]

She indicated that seeing the actual action sequences in space would be of interest to her.

"I guess it would be helpful to see how far you were going. It would be useful if you wanted to see what you did. If you were wasting any time doing stuff and getting everything done as quickly as you wanted to." :[L12]

POSITIVE REACTION (MILD): MEDICATION TAKING :[L12]

IDEA USE: MEDICATION TAKING TO DETERMINE WHERE TO KEEP PILLS :[L12]

She connected the medication adherence example with a possible solution.

"Well yeah, then you could have your pills there. So that would be helpful."

NEUTRAL REACTION: WHERE-WERE-YOU-WHEN AT HOME, ALREADY KNOWS SHE IS "MIXED UP" :[L12]

IDEA USE: WHERE-WERE-YOU-WHEN AT WORKPLACE :[L12]

The participant felt a more general "where were you when you were doing or not doing something" would be best for the workplace, not home.

"It would help for budgeting your time and keep you on schedule. It could show you where you were. I probably don't need help with it. I know I'm mixed up. It helps for when you're at work. It keeps people doing what they need to be doing. At work I could see it." :[L12]

NEUTRAL REACTION: LOW POINTS/HIGH POINTS WITH PLACE, ALREADY KNOWS :[L12]

IDEA USE: CONCENTRATION AND PLACE/TIME WHEN TAKING A CLASS :[L12]

She reiterated that she thought she knew when she was up and down and did not to see that information with regard to the place or situation.

"If I were taking a class, I would probably want to know when I could concentrate more. When would be the best time to take the classes or have tests. What would be the best time. Other than that I'm not really sure." :[L12]

IDEA USE: DETERMINING THE BEST TIME TO GO TO THE STORE OR TRAVELING :[L12]

"If you're talking about when the best time to go to the store when it was crowded or not crowded. Or if I wanted to travel somewhere." :[L12]

[Family] (0:53:00)

NEUTRAL REACTION: MORNING SCHEDULE – COULD BE BENEFICIAL WITH RESPECT TO DIVISION OF CHORES, BUT NOT FOR HER FAMILY :[L12]

CONCERN: FAMILY WOULD NOT BE WILLING TO CHANGE :[L12]

She could see the value in examining the morning routine, particularly for division of chores, but didn't think her family would be willing to talk about this information.

"They wouldn't want to do it. They like to do what they do when they do it. It wouldn't affect them." :[L12]

NEUTRAL REACTION: WATER USAGE, INSUFFICIENT MOTIVATION BECAUSE OF BILLING PROCESS :[L12]
IDEA VARIABLE: HOW AND WHERE FAMILY MEMBERS SPEND MONEY :[L12]

She recognized that the water tracking might help save water, but didn't think there would be sufficient motivation considering how billing was done. She did think her family would benefit from tracking how and where they were spending money.

NEUTRAL REACTION: EATING TOGETHER, ALREADY KNOW :[L12]

She didn't think the eating-together information would be needed.

"I don't see why we would want to track it, I think we realize it." :[L12]

NEUTRAL REACTION: READING, DOESN'T APPLY NOW THAT KIDS ARE OLDER :[L12]

She thought that the reading example might have applied when she had younger children, but not now.

NEUTRAL REACTION: CORRESPONDENCE, ALREADY KNOWS :[L12]

She thought she knew the correspondence information.

POSITIVE REACTION: FAMILY GROUP ACTIVITY :[L12]
BENEFIT: TRACKING WOULD TRIGGER MORE CONVERSATION, WHICH MIGHT TRIGGER MORE ACTION (FAMILY EXERCISE) :[L12]

She liked the group activity example and didn't think it would bother family members.

"That would be good. That would help if you are trying to lose weight or workout. If we talked about it more we would probably make sure we did it more." :[L12]

[Sorting] (0:59:36)

Resorted by "stress" **(1:11:00)**

ID: [L13]

Date: 8/31/04, 12:30 – 1:30 (60 minutes)
Participant type: Word of mouth
Demographics: Woman, age 55,
Location: Phone (New York)
Audio file: DM1000

- Shown 8 Displays: Journal, Snoop, TV, Mirror, Family, Years, Body, Daily Rhythm

Work: Researcher, neuroscience
Technologies: Cell phone, digital camera
Monitoring Devices: Scale, pedometer (but doesn't use)

[What I monitor] (0:02:06)
CURRENTLY MONITORING: WEIGHT :[L13]
CURRENTLY MONITORING: PULSE WHEN JOGGING :[L13]
She monitors her weight and keeps track of her heart-rate (by pulse) when she's jogging.
Doesn't keep a journal

[Journal](0:04:15)
NEUTRAL REACTION: PLACES VISITED :[L13]
NEUTRAL REACTION: PEOPLE ENCOUNTERED :[L13]
She wasn't interested in places visited or people encountered.

POSITIVE REACTION (HIGH): SNACKING :[L13]
She was enthusiastic about snacking and emphasized that she wanted something that would be easy.

> "Every time I diet, I start recording what I am eating, but either I lose the paper or I'm not at home when I'm eating, so I would love to be able to keep track of snacking." :[L13]

POSITIVE REACTION: WATER DRINKING :[L13]
She liked tracking water drinking, but thinks she may have that goal met because she uses a sports bottle.

POSITIVE REACTION: MEMORABLE MOMENTS, WHEN HAVE YOUNG KIDS OR GRANDKIDS :[L13]
She thought she would have loved to record memorable moments when her children were young, but is less interested now. She thought it might be important again when she has grandchildren.

POSITIVE REACTION: MEDIA, SOURCE OF CONTENT :[L13]
She wouldn't be interested in seeing the media related to her stage in life, but would like to be able to track down the source of content.

> "I think that would be important. Half the time I see something and can't remember when I saw it, and want to tell someone about it, so I would say that's important to me." :[L13]

[Snoop](0:07:20)
POSITIVE REACTION: EATING BEHAVIOR :[L13]
IDEA RELATIONSHIP: EATING AND BOREDOM, TASTE, IN FRONT OF THE TV, AWARENESS/MEMORY :[L13]
She would love having an outside source to give her information about her eating behaviors. I asked her what they would notice.

"That sometimes I eat things because I'm bored, that sometimes I eat things because they just taste so good, sometimes I'm eating in front of the TV and I can't even remember what I ate, other than I'm full." :[L13]

"I am totally unaware of what is going in my mouth. Totally. I can be on a diet and end up preparing food for someone else and I can't stop myself." :[L13]

POSITIVE REACTION: POSTURE, MILDLY CURIOUS :[L13]
She was only mildly curious about posture.

POSITIVE REACTION: OPPORTUNITIES FOR EXERCISE :[L13]
She liked the opportunities for exercise, wanting to know what opportunities she was overlooking.

POSITIVE REACTION: USE OF HEDGES IN PRESENTATIONS, LOW PRIORITY :[L13]
The social awareness example that stood out to her was the use of hedges in presentations. She thinks she is not as fluent as she could be, but it's low on her priority list.

POSITIVE REACTION: WORK HABITS, EFFICIENCY :[L13]
IDEA INVESTIGATION: EFFICIENCY AND MULTITASKING, COMPLETING TASKS :[L13]
"I wouldn't mind someone telling me how I could be a little more time-efficient, I tend to start a lot of different things and have problems finishing everything. So I wouldn't mind taking a look at that." :[L13]

She doesn't get distracted by external factors such as printer noise, but admits that she will get off-course on her current task by thinking of something else that needs to be done.

She's comfortable with the idea of having someone offer observations.

[TV](0:12:25)
She laughed at the idea of the mood rating and the time spent in conversation.

NEUTRAL REACTION: TV WATCHING, IT ISN'T AN "ISSUE" :[L13]
CONCERN: BEHAVIOR IS TOO ERRATIC TO SUGGEST A MEANINGFUL PATTERN (TV WATCHING) :[L13]
She does watch TV, and falls asleep watching it at night, but doesn't feel it is an issue for her and wouldn't find tracking it to be important. She thinks her TV watching is very erratic, so one week's time wouldn't necessarily offer clues about her general TV watching behavior.

IDEA VARIABLE: TIME SPENT WORKING, INCLUDING RESPONDING TO EMAILS AT HOME, READING PAPERS AT HOME :[L13]
I asked her if there was anything else for which she would like to examine duration of time spent.
"I wouldn't knowing in reality how much time I spend working. I'm obviously here at the office, but I respond to emails at home, I read papers, I would love to know how many hours I really put in." :[L13]

IDEA RELATIONSHIP: TIME SPENT WORKING AND TIME SPENT ON ACTIVITIES "FOR ME," PREPARING DINNER, GETTING A MANICURE, EXERCISING, ONLINE SHOPPING :[L13]
BENEFIT: GET LIFE IN ORDER, GET A BETTER SENSE OF PERSPECTIVE :[L13]
I point out the relationships aspect of this chart. I ask her if there was anything she would want to know in relationship to time spent on work.
"I guess I would like to know how much time I spend on things for me, preparing dinner, how many hours I spend getting a manicure or exercising or ordering things online, things like that I wouldn't mind seeing, maybe I could get my life in order here and get

a better sense of perspective. Relationship of me time versus everything else I do in the world." :[L13]

NEGATIVE REACTION: COMPARING TO AVERAGES OR ESTIMATES – PREFERS "HARD FACTS" :[L13]
I asked her about comparing to averages or estimates. She didn't have interest in that, preferring the "hard facts" and wouldn't care much about the average.

[Mirror](0:15:45)
NEUTRAL REACTION: HYGIENE ROUTINES, ALREADY DILIGENT AND EFFICIENT :[L13]
She feels that she is diligent and efficient about her hygiene routines so wouldn't need additional information.

POSITIVE REACTION: REFLECTION OF WHAT WEARING :[L13]
IDEA VARIABLE: WHAT CLOTHING HAS BEEN WORN LATELY :[L13]
"I'm getting to the age where I can't remember what I wore a couple of days before, that would be real helpful. I'm sure the world is talking about me wearing the same thing – but to me it's important, and when I get home it's a blur because I hang it up." :[L13]

CONCERN: REFLECTION MIGHT BE DEPRESSING :[L13]
POSITIVE REACTION: REFLECTION OF APPEARANCE, FUN, BUT FRIVOLOUS :[L13]
IDEA VARIABLE: HOW YOU WEAR YOUR HAIR :[L13]
She thought seeing the reflection of herself from a year ago might be depressing, but also fun and ultimately frivolous.
"I might like to remember how I was wearing my hair." :[L13]

POSITIVE REACTION: THINGS THAT HAPPENED THE DAY BEFORE :[L13]
BENEFIT: JOG MEMORY :[L13]
NEUTRAL REACTION: PHOTOS OF PEOPLE ENCOUNTERED – IF IMPORTANT, WOULD RECALL ANYWAY :[L13]
She liked the idea of being reminded about things that happened the day before and felt that she wouldn't need much to jog her memory. She didn't feel she would need the photos of people encountered, because if it were an important person, she would recall.
"I don't know how specific I would get, but I wouldn't mind remembering some of the things I did the day before."

CONCERN: EFFORT REQUIRED :[L13]
"Any of these things are interesting, if there is a way to do them without a lot of effort from me." :[L13]

NEUTRAL REACTION: PHOTOS OF PEOPLE WILL ENCOUNTER, DAY-TIMER ALREADY FILLS THIS ROLE :[L13]
She feels her day planner prepares her for the day (without need for something like photos of the people she would meet).

[Family](0:19:40)
NEUTRAL REACTION: FAMILY SCHEDULE :[L13]
She didn't have an interest in the family schedule.

NEUTRAL REACTION: WATER CONSERVATION, GLUT OF WATER :[L13]
IDEA VARIABLE: ELECTRICITY AND HEATING :[L13]
IDEA INVESTIGATION: DOES TURNING OFF TV, TURNING OFF COMPUTER SIGNIFICANTLY REDUCE ELECTRICITY :[L13]
She doesn't think about water conservation because they have a glut of water. She could see it for electricity and heating. She gets a bill, but if she could assess when things (e.g., the computer, the TV) could be turned off that would really make a difference.

POSITIVE REACTION: EATING MEALS TOGETHER – INTERESTING BUT NOT MANDATORY :[L13]
NEUTRAL REACTION: READING – KIDS ARE OLDER NOW :[L13]
NEUTRAL REACTION: GROUP SCHEDULE – KIDS ARE OLDER NOW :[L13]

She would find the eating meals together information interesting, but "absolutely not mandatory." She didn't feel reading or group activity levels were an issue in their house any more because the kids were older.

POSITIVE REACTION: CORRESPONDENCE WITH FRIENDS/FAMILY, LAST TIME CONTACTED ONLY :[L13]
IDEA USE: CORRESPONDENCE TO REMIND OVERDUE :[L13]

With regard to correspondence with friends and family, she likes the idea, but doesn't believe the medium of the contact would be relevant.

> "I would love to keep track of that. I always feel like I'm behind, I owe someone a call, somebody I haven't spoken to in a while, that would be kind of nice, something to remind me I'm overdue." :[L13]

[Years] (0:22:25)

POSITIVE REACTION: TIME SPENT COOKING OVER LONG-TERM – INTERESTING, NOT MANDATORY :[L13]
POSITIVE REACTION: CABINET ACCESSES OVER LONG-TERM – INTERESTING, NOT MANDATORY :[L13]
CONCERN: SOME INFORMATION MAY BE INTERESTING BUT NOT HELPFUL :[L13]

She would find time spent cooking and cabinet accesses "not mandatory, but very interesting." She doesn't think it would help her at all.

NEUTRAL REACTION: MICROWAVE VERSUS STOVETOP :[L13]

She wasn't interested in microwave versus stovetop.

CONCERN: LONG-TERM CHANGES MAY MAKE MOROSE, LONGING FOR THE PAST (TIME SPENT WITH FAMILY) :[L13]
IDEA VARIABLE: TIME SPENT WITH FAMILY OVER LONG-TERM :[L13]
IDEA VARIABLE: EXERCISE OVER LONG-TERM :[L13]
IDEA INVESTIGATION: CHANGE IN EXERCISE VERSUS PERCEPTION OF CHANGE; CONTRIBUTION OF LIFESTYLE REQUIREMENTS (HAVING KIDS) :[L13]

She would be interested in seeing time spent with family, but admits that it might make her morose. She would be interested in exercise.

> "I would probably be interested in exercise, then and now, I feel like I'm actually do more now, but then I was running up and down the stairs." :[L13]

IDEA INVESTIGATION: HOW COOKING TIME CHANGES AS HOUSEHOLD CHANGES, REGULAR COOKING VERSUS COOKING ON SPECIAL OCCASIONS :[L13]

For the next ten years she would like exercise, time spent with family, and cooking. She believes that once everyone is out of the house, she won't be doing much cooking, except for special occasions.

> "It wouldn't change my life, but it would be interesting to see." :[L13]

POSITIVE REACTION (STRONG): SHORT-TERM MEMORY OVER TIME :[L13]
POSITIVE REACTION: COMPARING SHORT-TERM MEMORY WITH FRIENDS :[L13]
CONCERN: INFORMATION MAY BE DEPRESSING (SHORT-TERM MEMORY OVER TIME) :[L13]

> "I would love to see that [short-term memory]. Again, it would probably be depressing, I certainly know enough about short-term memory and aging, I would see the decrement, but I would still like to see it. I think that would be very valuable. I think I'm right in there with my friends, but again I wouldn't mind comparing myself to others. I would find that interesting and very useful." :[L13]

IDEA VARIABLE: SHORT-TERM MEMORY LAPSES, SUCH AS LEAVING GROCERY LIST AT HOME, FORGETTING PHONE NUMBER/ADDRESS :[L13]

"If it were at all possible to have variables that were really part of my life, like I left my grocery list at home or I can't remember what I did yesterday, it's very hard for me to retrieve that, or a phone number or address, whatever it happened to be. That would be the more practical thing, I don't know how doable that is." :[L13]

CONCERN: RELIABILITY OF ASSESSMENT, VALIDITY - WHETHER ASSESSMENT REFLECTS CHALLENGES OF REAL WORLD :[L13]

"I'd be willing to take a game, but I don't know how reliable that is. If it were a game, I would be giving it full concentration and ignoring anything extraneous, whereas in the real world, I try to remember something and there are other things going on." :[L13]

IDEA USE: SHORT-TERM MEMORY TRACKING TO TRIGGER MORE INTENSIVE USE-IT-OR-LOSE-IT EFFORTS :[L13]

She would try to use her brain a little bit more, according to the use it or lose it strategy. She feels like she is using her brain a lot while she works, but once she is retired she might try doing games.

"Maybe I would be more diligent in making associations, once I know that, I might make more effort, if I met people…" :[L13]

IDEA RELATIONSHIP: MOOD AND MENOPAUSE :[L13]

She doesn't think her mood varies much now, but imagines that her mood might vary more once she goes through menopause. She feels her short-term memory is worse, but that it doesn't get in the way of her life.

[Body] (0:29:20)

NEUTRAL REACTION: SKIN CHANGES, ALREADY DILIGENT BECAUSE KNOWS AN ISSUE :[L13]

She's out in the sun a lot and is very diligent already about examining her skin for changes.

NEGATIVE REACTION: ACTIVITY INFORMATION, TOO MICRO :[L13]
CONCERN: INFORMATION IS TOO MICRO (BODY ACTIVITY) :[L13]

She felt the activity information was "a little micro"

"I couldn't imagine altering my activity because I wasn't doing enough torso activity or my arms. I wouldn't let that get in a way of what my life was like. It's too micro for me." :[L13]

NEGATIVE REACTION: MUSCLE TONE :[L13]
NEGATIVE REACTION: EXERCISE :[L13]
CONCERN: DON'T WANT TO FOCUS ON SOMETHING YOU DON'T ENJOY, DOING BECAUSE YOU HAVE TO (EXERCISE) :[L13]

She felt the same way about the muscle tone and exercise. She hates to exercise and does it because she has to.

POSITIVE REACTION (STRONG): ACHES AND PAINS, FREQUENCY :[L13]

She liked aches and pains.

"You know what, this would be interesting, because I do have headaches and I'm not good at keeping track of – it's in the same area, in the frontal area, but I'm not good at tracking when I get them. I'd like to look for a pattern. I'd love to keep track of it." :[L13]

IDEA INVESTIGATION: CAUSE OF HEADACHES :[L13]
POSITIVE REACTION: HEADACHE AND DAILY EVENTS, STRESS :[L13]

She agreed that having additional information about what was going on during the day or stress would be helpful, because she has "no idea" about possible relationships with her headaches.

[Daily Rhythm] (0:32:45)

POSITIVE REACTION (MILD): DAILY RHYTHM, INTERESTING BUT NOT USEFUL :[L13]
She thought it might be interesting to see, but couldn't imagine having any use for it. She feels like the things in her life that she needs to change she already knows about and wouldn't need more information.

CONCERN: THINGS IN LIFE THAT NEED TO CHANGE (MORE SLEEP, MORE EXERCISE, LESS FOOD, ALREADY KNOW ABOUT; HAVING ADDITIONAL INFORMATION WOULDN'T BE HELPFUL :[L13]
"I feel like my life will continue the way it is. I don't think this would change. There are certain things I know I need that I just can't seem to get. I know I need more sleep, I need more exercise, I need less food." :[L13]

BENEFIT: SEEING SOMETHING "IN BLACK AND WHITE" COULD TRIGGER A CHANGE (EATING) :[L13]
"[eating and snacking] that part I may need some help, maybe if I saw it in black and white, I might change a little." :[L13]

[Sorting] (0:34:45)
IDEA RELATIONSHIP: TIME OF DAY AND CONCENTRATION/ALERTNESS :[L13]
She would like to look at alertness and concentration – she feels she knows that time of day affects her, but would like to see it.
She thinks it may be hopeless in terms of improving her recall of jokes and stories, but would like to see it.
CONCERN: ALREADY A SOURCE OF CONFLICT WITH FAMILY MEMBER (ADJUSTMENTS TO THERMOSTAT) :[L13]
She and her husband battle over the thermostat all the time – and it might be interesting to prove a point, but she decides against tracking it.
She only drinks a glass of wine occasionally.
IDEA RELATIONSHIP: HEADACHES AND BAROMETRIC PRESSURE :[L13]
She thinks barometric pressure may trigger her headaches.
She feels like she is in the bathroom all the time.
She thinks blood pressure may be an issue for her one day.
She would like to keep track of what she's worn recently.
She feels like she does what she has to do with regard to commitments.
She knows the traffic patterns.
She feels like she knows about her email patterns.
IDEA USE: JUST INVESTIGATE ONCE OR TWICE (FOOTSTEPS) :[L13]
She would like to see footsteps once or twice.
She'd like to think she isn't naïve.
She does take her pulse when she jogs, so she includes heart rate.
She's interested in hormones with respect to menopause.
She has no idle time. <laughs>
BENEFIT: DIFFICULT TO READ YOURSELF WELL ON PERSONALITY VARIABLES (IMPULSIVENESS) :[L13]
She doesn't think she is impulsive, but thinks she may not be reading herself well.
She thinks she laughs a lot.
She's very interested in multitasking because she does it, but doesn't think she does it well.
Her night vision is not as good any more.
She just keeps her photos on her computer.
She is concerned that her hearing will deteriorate and would like to monitor it.
She thinks the fridge open/close would be funny, but she doesn't care.
Doesn't smoke.
IDEA VARIABLE: LEISURE TIME :[L13]
Time spent with friends and leisure time.
IDEA VARIABLE: TEMPERATURE SENSITIVITY :[L13]

She's interested in touch and temperature sensitivity. Sometimes she'll have food on her face and she won't feel it.

Resorts according to eating choices.

IDEA RELATIONSHIP: AWARENESS OF TIME AND DECISION TO EAT :[L13]

She's interested in awareness of time with respect to when she decides to eat
Blood glucose and blood pressure should be, but she's "just not interested."

IDEA RELATIONSHIP: MULTITASKING AND UNCONTROLLED EATING :[L13]

She thinks she stuffs things in her mouth when she is multitasking

IDEA RELATIONSHIP: SHORT TERM MEMORY AND EATING – NOT REMEMBERING WHAT YOU'VE EATEN :[L13]

She thinks not remembering what she eats might be related.

IDEA RELATIONSHIP: EATING TRIGGERED BY TRIP TO THE GROCERY STORE :[L13]

She rips the bags open right after she gets food at the grocery store.

"I clicked on things that I wasn't really interested in before." :[L13]

IDEA USE: ONE INVESTIGATION (HEADACHES, EXERCISE) AT A TIME :[L13]

She would like to do specific investigations, like understanding her headaches, looking at exercise – doing investigation things individually.

ID: [L14]

Date: 8/26/04, 5:00 – 6:00 (50 minutes)
Participant type: Word of mouth
Demographics: Woman, 1948
Location: Phone (Washington)
Audio file: DM100024

- Shown 11 Displays: Journal, Snoop, Years, Place, Body, Performance, Grocery, Voicemail, Family, TV, Activity
- Believes she already knows a lot about herself, low interest in tracking
- Not interested in seeing information that demonstrates cognitive decline
- Was interested in data that could tell her whether she was doing enough, activity-wise
- Had some interest in eating and spending tracking
- Would prefer background tracking to investigations

Work: High school teacher
Technologies: No cell phone, no digital camera, no pda
Monitoring devices: No scale or other devices

[What I monitor] (0:02:50)
Doesn't keep a journal.

[Journal](0:04:30)
POSITIVE REACTION: PLACES VISITED :[L14]
POSITIVE REACTION: MEMORABLE MOMENTS :[L14]
She would keep a journal of places visited and memorable moments.

She is reminded that she kept a journal last summer when she went on a trip.

NEUTRAL REACTION: SNACKING :[L14]
NEUTRAL REACTION: WATER DRINKING :[L14]
NEUTRAL REACTION: PEOPLE ENCOUNTERED :[L14]
NEUTRAL REACTION: MEDIA :[L14]
She wasn't interested in the other examples.

[Snoop](0:06:00)
POSITIVE REACTION: EATING BEHAVIORS :[L14]
POSITIVE REACTION: OPPORTUNITIES FOR EXERCISE :[L14]
POSITIVE REACTION: SOCIAL AWARENESS :[L14]
She likes eating behaviors, opportunities for exercise, and social awareness.

IDEA INVESTIGATION: APPROPRIATE SCHEDULING OF EATING TIMES :[L14]
IDEA USE: FINDING WAYS TO INCORPORATE EXERCISE INTO THE WORK DAY :[L14]
If the detective followed her around for a week, she would like them to notice whether she's eating the right amount at the right time. She eats lunch and is then hungry again when she gets home, but it's too early for dinner. She would like to find more opportunities for exercise during the work day.
IDEA VARIABLE: ABILITY TO RECOGNIZE WHEN OTHER PEOPLE NEED ASSISTANCES (STUDENTS) :[L14]
"I'm always interested in ways, particularly with my students, of making good contact with them. Having students know how much I care, because then they are more willing to do more. Ways of – because I don't always think of what I want to say, and later I think

of it – particularly with students, you want to pick up on a cry for help and you maybe don't hear it." :[L14]

She thinks someone might be able to show her how to pick up on these signals from her students sooner.

CONCERN: DISRUPT ILLUSIONS ABOUT SELF – BUT NOT ALWAYS A BAD THING :[L14]

"You never like to hear you are not a 100% good, but I don't suppose it would be too bad." :[L14]

NEUTRAL REACTION: POSTURE :[L14]

NEUTRAL REACTION: WORK HABITS :[L14]

She doesn't feel posture or work habits would be anything she would need to work on.

[Years](0:09:30)

NEUTRAL REACTION: TRACKING OVER YEARS TIME PERIOD, KNOW ALL NEED TO KNOW :[L14]

Ten years previous her life was much different, because her two children living with her. One of her daughters is just out of college and will be living with her for a brief period.

She knows her short-term memory has "gone downhill fast."

"I don't know if there is anything different about me that I don't know that I would want to know." :[L14]

I asked her to consider the next 10 years, particularly with regard to things that would indicate significant change. She couldn't think of anything in the moment.

I asked her to consider a time when she went through a period of change, such as depression. She felt that there wasn't anything in particular that would have described that period, and felt that she was self-aware of the experience.

"It became difficult to do almost anything, I can't think of anything particular that changed. I stared at the tube because I didn't do anything yes." :[L14]

CONCERN: SEEING DECLINE IS UNPLEASANT (SHORT TERM MEMORY) :[L14]

IDEA USE: REASSURANCE ONLY THAT SHORT TERM MEMORY IS WITHIN RANGE ONCE A WEEK :[L14]

POSITIVE REACTION: COMPARING SHORT-TERM MEMORY TO PEOPLE IN THE AGE RANGE :[L14]

I asked her about the short-term memory example, specifically with regard to the comparison with friends.

"I know that it's going down, I don't need to know any more of that. As you get older, you worry about Alzheimer's. I just need everything now and then to be reassured that this is normal and I suppose it would be nice to know other people have the same problem I do." :[L14]

POSITIVE REACTION (MIXED): SHORT-TERM MEMORY, TO INITIATE EFFORTS TO COUNTERACT IF SHARP DECLINE :[L14]

I asked her how often she would like to check in on her memory. She laughed and said maybe once a week. I asked her if she saw that it was declining more than other people her age whether it would still be information she would like to have.

"Yeah, because then I would like to find out if there is anything I should do about it or if there is anything that can be done about it." :[L14]

I asked her how variable she thought her memory was.

“All I know, is that over time, over the last twenty years, it’s gone downhill. For example, every year, it takes me longer to learn all the students’ names. That kind of thing.” :[L14]

NEUTRAL REACTION: TIME WITH FRIENDS OVER YEARS :[L14]

I asked her about the time with friends metric.

“I have no feeling that that would vary over time or that I would get anything from that.” :[L14]

[Place](0:16:10)

NEUTRAL REACTION: ACTIVITY PATTERNS, CAN’T THINK OF HEALTH APPLICATION :[L14]

She couldn’t imagine wanting to have the activity patterns in space information unless she was trying to redesign her home.

NEUTRAL REACTION: MEDICATION PATTERNS, ALREADY MASTERED :[L14]

I had her look at the medication example and she offered that she does take cholesterol medication. She thought it might be useful to determine where to put medication, but she already feels she has a good system, where she places her medication on the counter in the morning to remind herself in the evening.

She doesn’t feel that she puts off activities and feels she develops a consistent routine with built-in reminders for most things.

NEUTRAL REACTION: LOW POINTS/HIGH POINTS, ALREADY UNDERSTAND WHY HAPPY OR NOT :[L14]

CONCERN: TRACKING MOOD IS FOR PEOPLE WHO ARE NOT SELF-AWARE :[L14]

I asked her to take a look at the high point/low point example.

“I can imagine that this might be helpful to someone, but it would have to be someone who is not very self-aware, like would have no idea why they are feeling happy or not feeling happy. I don’t tend to be like that, so I couldn’t imagine needing that.” :[L14]

[Body](0:21:25)

POSITIVE REACTION: SKIN RECORD, IF YOU HAVE A SPOT THAT CONCERNS YOU :[L14]

She wanted to know how a skin record would be tracked

“I could see that, if you have a spot and want to see how it changes over time.” :[L14]

POSITIVE REACTION: ACTIVITY BY BODY REGION, TRACK RELATIVE TO GOAL :[L14]

She would like to track the activity and likes how it is set-up by body limb, but suggests that the relevant goal needs to be listed.

POSITIVE REACTION: EXERCISE :[L14]

BENEFIT: OTHERWISE DIFFICULT TO DETECT IMPROVEMENT IN INITIAL STAGES OF EXERCISE ROUTINE :[L14]

She thought that muscle tone would be good for an exercise program

“That would be good, if you were entering on an exercise program, and at first you wouldn’t notice that anything had improved, but if you had something like this telling you things are improving, that might encourage you to keep going. I like that idea.” :[L14]

IDEA INVESTIGATION: HAVE YOU DONE ENOUGH EXERCISE IN THE COURSE OF EVERYDAY ACTIVITIES :[L14]

“I think I would know how much exercise I’ve done, I think I would need to know how much I need to do, that’s why I like the activity one. If you are doing bicep curls, you know how much you’ve done, but if you walk around on a given day and do your life, is that enough to keep you healthy, do you need to do more? That would be interesting.” :[L14]

"I know when I have aches and pain… " I asked her if she knows what causes the pain.

POSITIVE REACTION: PAIN, UNDERSTANDING TRIGGER FOR BURSITIS :[L14]

"I suppose - I get this reoccurring bursitis and if I keep track of it, maybe I would know what triggers it. I can see that." :[L14]

IDEA RELATIONSHIP: PAIN AND PHYSICAL ACTIVITY FROM PREVIOUS DAY :[L14]

I asked her what she would need to track to understand what is causing the pain.

"I guess you would need to know what happened before, like the day before, to set it off. You would need to keep track of what your physical activities that led up to that." :[L14]

[Performance] (0:24:50)

CONCERN: DOWNHILL PERFORMANCE WOULD BE DEPRESSING, AND THAT'S INEVITABLE WITH AGE :[L14]

CONCERN: IS THERE SOMETHING YOU CAN DO ABOUT IT? IS IT ACTIONABLE? (MEMORY) :[L14]

"That would be so depressing, because you would do nothing but go downhill at my age. But I think it would be interesting to keep track of it. The important thing about these things, if you are keeping track of short term memory or your performance or whatever – the other thing that goes along with it, if this is something that is deteriorating faster than it should, is there something you can do about it? Otherwise you are just documenting your decline." :[L14]

I asked her if she expected that she would see variability based on time of day or what she was doing.

CONCERN: DOESN'T MATTER IF CONTEXT AFFECTS YOU (TIME OF DAY AND MENTAL ABILITY), BECAUSE NO CONTROL OVER CONTEXT (MUST DO WORK AT SPECIFIC TIME) :[L14]

"I don't know, but if I did, it wouldn't matter much, because I go to work and I have to be – class starts at 7:20 here and I have to be performing. It wouldn't matter to me if I found that I performed better at a different time of day because there is nothing I could do about it." :[L14]

She keeps a structured schedule in terms of sleep.

IDEA USE: DON'T PROBLEM SOLVE, JUST DOCUMENT DECLINE TO TAKE TO DOCTOR :[L14]

IDEA INVESTIGATION: DOES EXERCISE IMPACT MEMORY :[L14]

She doesn't want to problem solve, but instead would want to just have documentation of physical or mental decline over time so that she could take it to a doctor to see if anything could be done. She would try exercising more to see if it affects these variables, but is skeptical that engagement in stimulating activities is helpful, because she is a chemistry teacher and still experiences mental changes.

[Grocery] (0:27:50)

POSITIVE REACTION: SPENDING, WITH BREAKDOWN :[L14]

POSITIVE REACTION: NUTRITION GOALS :[L14]

She was interested in spending and meets nutritional goals.

"That [the breakdown of spending by snacking, basic, and non-food] is what I thought was interesting. You go to the store and spend this money… at the end of the month, you want to add up how much you spent on groceries, and for a matter of fact, how much of it was spent on food versus non-food and snacking. I think that might be interesting." :[L14]

IDEA USE: GROCERY RECEIPT, NOT GROCERY LIST, IS APPROPRIATE TIME/PLACE FOR DELIVERY BECAUSE IT'S THE IMPULSE BUYS THAT ARE RELEVANT :[L14]

Her nutrition goals would have to do with vitamins, fiber, and low-fat (not low-carb). I asked her about the timing of presentation.

"I think it would be the only practical time. I don't think you could get it ahead of time, because if you were disciplined and knew here's what you were going to by and then were told about whether they met your nutrition goals, this assumes that you were just going to go in and follow that, that shopping plan. Well if you were that disciplined, you wouldn't be buying the crap in the first place. It would be okay to get it afterward because maybe in the course of the month you would be looking at these things and go oh my god, look at all the crap I bought, maybe it would make a difference in what you bought." :[L14]

[Voice Mail] (0:30:30)

NEUTRAL REACTION: VOICE MAIL, EMAIL ALREADY PROVIDES SUFFICIENT CONTACT :[L14]

"I couldn't see this being any improvement over email." Her mother gives her updates about her life by email 4 or 5 times a day.

NEGATIVE REACTION: VOICE MAIL, INTRUSIVE :[L14]
CONCERN: MONITORING FAMILY MEMBERS IS INTRUSIVE :[L14]

"That seems a little intrusive to me, I guess I don't like the big brother aspect." :[L14]

I asked her if family members have trouble communicating information about their life or health and she said that her son doesn't tell her anything, but that he wouldn't want something automatically sent either.

[Family] (0:32:50)

She noted that most of these examples only apply to households with multiple members.

NEUTRAL REACTION: CORRESPONDENCE, ONLY USES EMAIL :[L14]

All her extended correspondence is through use of email, so she didn't feel she would need additional information about that.

POSITIVE REACTION: WATER USAGE, EXPERIENCE WITH IN PAST WAS POSITIVE :[L14]
NEUTRAL REACTION: WATER USAGE, NOT NEEDED WITH SINGLE PERSON HOUSEHOLD :[L14]

She had her students keep a "water diary" years ago and did find it to be a valuable experience for herself and her students. She found out that over half of her water went to watering the lawn.

"It did make a difference in my life. It changed how we used water." :[L14]

[TV] (0:35:40)

NEGATIVE REACTION: TIME SPENT DOING ACTIVITY, HAPPY WITH HOW SPENDING TIME :[L14]

"I can't see any point where I say, oh I want to have something that keeps track of how much time I spend doing anything. I don't see any need for it." :[L14]

"It's not a problem for me. I don't perceive it as a problem. Probably because I don't have my children with me, I don't have this feeling of oh I can't get everything I need to done, where is my time being wasted, I don't have a problem with it. I'm happy with how I'm spending my time." :[L14]

[Activity] (0:38:15)

POSITIVE REACTION: WALKING ACTIVITY :[L14]
IDEA RELATIONSHIP: ACTIVITY ON WEEKENDS VERSUS WEEKDAYS, ACTIVITY AT WORK VERSUS VACATION :[L14]

She chose this one on her own and liked it. She would like to see daily and then weekends versus weekdays, work time versus vacation.

She feels she wouldn't need additional information about what she was doing to make sense of the information.

[Sorting] (0:39:50)

IDEA VARIABLE: HOT FLASHES :[L14]

Sometimes she thinks she is having hot flashes and would like to understand her internal thermostat (whether she's feeling hot or cold) – she notes that she doesn't always adjust the thermostat though.
She thought clothing choices might be relevant though.
She already knows that community traffic congestion is bad.
She resorts by physical activity and for mental sharpness.

She wouldn't want to put a lot of mental effort into tracking and would rather have something that would remind her to check on values.

IDEA USE: TRACKING THE BACKGROUND :[L14]

She would rather have things tracked in the background over a long period rather than initiating investigations.

ID: [L15]

Date: 8/31/04, 5:00 – 6:00 (55 minutes)
Participant type: Word of mouth
Demographics: Woman, age 57
Location: Phone (Washington, DC)
Audio file: DM1000

- Shown 7 Displays: Journal, Snoop, TV, Place, Daily Rhythm, Activity, Years

Work: Part-time, self-employed accountant, was a nutritionist in training
Technologies: cell phone, digital camera, no PDA, laptop
Monitoring Devices: scale in bedroom, no pedometer, no heart rate monitor, sometimes checks on blood pressure at store

[What I monitor] (0:05:10)

CURRENTLY MONITORING: BLOOD PRESSURE :[L15]

She occasionally will check her blood pressure at the store, but only when she thinks of it (she doesn't make trips to the store for that purpose).

She was surprised recently to have her blood pressure taken and it be higher than her normal, so she is now more interested in tracking it.

CURRENTLY MONITORING: WEIGHT :[L15]

She also tracks her weight and would like to lose some weight for general health.

[Journal](0:09:30)

PROCEDURE: CONFUSION ABOUT STUDY REQUIREMENTS :[L15]

She was initially concerned that she would actually have to start keeping a journal as part of the study, but was reassured that the displays were just hypothetical.

CURRENTLY MONITORING: TO-DO'S :[L15]

She already uses a personal calendar and has from time to time recorded what she did for the day over a period of several years, but she never goes back to look at it.

CONCERN: NOT KNOWING WHAT TO DO WITH INFORMATION :[L15]

After a few years, I don't do it anymore, because I just don't know what to do with that information. :[L15]

BENEFIT: TRACING START OF MEDICAL PROBLEM :[L15]

She admits that she would look at the information over a short-term basis, particularly when some problem occurred for which she sought medical assistance. She would look back in the calendar to try to determine when the problem first occurred. She only did this once or twice.

BENEFIT: INFORMATION USED TO PLAN (PERSONAL CALENDAR) CAN ALSO BE USED TO TRACK :[L15]
BENEFIT: RECORDING INFORMATION ABOUT DAILY EVENTS IS NOT HARD :[L15]

She mainly uses her calendar for appointments and believes that information could also be helpful retrospectively. She asserts that she would be willing to record more about what happened during a day if there was use for it, because the act of doing it is not hard.

NEUTRAL REACTION: SNACKING – ALREADY KNOW, MOST EATING FOLLOWS ROUTINE :[L15]
She feels that she is fairly conscientious about what she eats and therefore does not need to track snacking. She believes she can recall what she eats during the day and with some effort, what she ate the day previous. She believes she has a fairly strong routine with regard to what she eats.

[Snoop](0:16:05)
PROCEDURE: CONFUSION OVER WHETHER TO ANSWER FOR OTHER PEOPLE OR FOR SELF :[L15]
She asked for clarification about whether it would be helpful for other people or for her case.

POSITIVE REACTION: SNOOP, EATING BEHAVIOR :[L15]
Oh, I think it would be very helpful. Even though I studied nutrition, I do think there might be something I don't realize. Mistakes I made, or I should do better.. yeah, I think that would be very good. :[L15]

IDEA VARIABLE: QUANTITY OF FOOD :[L15]
IDEA VARIABLE: FOOD CHOICES :[L15]
IDEA VARIABLE: POSTURE AND EATING MANNER :[L15]
She was interested in getting feedback about the quantity of food she is eating, but particularly about posture and eating manner and choices of food.
She agreed that feedback about exercise would be helpful too.

IDEA USE: EXERCISE FEEDBACK SHOULD BE GENTLE AND JUST FOCUS ON HELPING PERSON TO GET STARTED :[L15]
In a gentle way. If I wasn't moving around, if they could remind me to do a little bit more – "try 20 hand lifts or bend your knees, try a little bit." A little push, because I know the initiation is so hard, but once I get on to doing something, I really enjoy it. :[L15]

PROCEDURE: THINKS ABOUT SOCIAL AWARENESS AND WORK HABITS AS APPLIED TO OTHER PEOPLE :[L15]
She thinks the social awareness and work habits are helpful, but talks about them more hypothetically ("people can get into bad habits…"). She notes that she has noticed food service employees sometime have non-hygienic habits, such as touching their face. She also would like to see politeness addressed.

IDEA USE: A COMPUTER GIVING FEEDBACK WOULD BE SEEM LESS CRITICAL :[L15]
She emphasizes that she wouldn't want criticism, but thinks a computer telling her would be better.

[TV](0:21:20)
NEUTRAL REACTION: FAIRLY AWARE OF HOW MUCH TIME SPENT WATCHING TV :[L15]
POSITIVE REACTION: TIME SPENT OUTSIDE :[L15]
She thinks they are all very interesting. She's "kind of aware" of how much time she spends watching TV. She notes that "time spent outside" would be helpful because if it's very low, "you are not spending enough time with nature and are instead cooped up".

NEUTRAL REACTION: MOOD, DOESN'T VARY SIGNIFICANTLY :[L15]
She thinks it would be interesting to see time spent outside with mood, but believes that her mood is generally not varied; she doesn't have significant mood swings.

NEGATIVE REACTION: COMPARISON WITH AVERAGE, MAY NOT BE HELPFUL OR ACCURATE :[L15]
NEUTRAL REACTION: COMPARISON WITH PERCEPTION OF TIME SPENT IN BEHAVIOR (NO REACTION) :[L15]
POSITIVE REACTION: TIME SPENT WATCHING TV WITH TIME SPENT OUTSIDE :[L15]

She's not sure about making comparisons with the average, because sometimes it is not very accurate or helpful. She didn't respond to the idea of comparing the watched time with the perceived watched time, but instead concentrated on seeing the relationship between TV watching and time spent outside.

NEUTRAL REACTION: TIME SPENT IN CONVERSATION, UNSURE THAT WOULD BE RELATED TO OTHER VARIABLES :[L15]

I asked her about tracking time spent in conversation and she wondered whether that would really be related to time spent watching TV. She was unsure, in general, about whether tracking conversation time would be helpful.

[Daily Rhythm](0:25:28)

POSITIVE REACTION: DAILY RHYTHM AS GOOD OVERALL PICTURE :[L15]

She thought it would be a good overall picture of how she was spending her time, what she was doing more or less, and what she should be concentrating on.

IDEA VARIABLE: CLEANING :[L15]
IDEA VARIABLE: TIME SPENT ON PHONE :[L15]
IDEA VARIABLE: TIME SPENT (WORKING) ON THE HOUSE :[L15]
IDEA VARIABLE: TIME SPENT COOKING :[L15]
IDEA VARIABLE: TIME SPENT ON LEARNING OR READING :[L15]
IDEA VARIABLE: TIME SPENT ON SOCIAL :[L15] :[L15]
IDEA VARIABLE: OUTINGS, SUCH AS GOING TO MOVIE THEATRE

> Maybe...hmm, I don't know about the categories. It would be different for each person. Maybe a little simpler, cleaning, or time spent on the phone, time spent on the house, cooking, time spent on learning or reading, social, outings, going to the movie theatre.

IDEA USE: EVALUATE TIME SPENT ON ACTIVITIES ON DAILY BASIS TO DETERMINE WHERE ADJUSTMENTS COULD BE MADE TO MAKE MORE TIME FOR GOALS :[L15]

After a year or two, she would evaluate whether she was meeting her goals and if not, begin assessing whether she could better manage her time in order to achieve them.

> After a year or two, I think oh, I'm not progressing along or I didn't fulfill some of my wishes or whatever, and maybe look at the pattern and go "oh! Maybe I should cut down on this, increase this a little bit more, to progress more. Find out why and help to improve. :[L15]

[Place](0:2:) – participant chosen

The participant indicated she was familiar with plan view. She reflected that the sequence of activity information was "anthropology-like."

BENEFIT: ACTIVITY SEQUENCE FOR HOME DESIGN, DEVELOPING CENTRALIZED DESIGN :[L15]
NEUTRAL REACTION: ACTIVITY SEQUENCE, ALREADY KNOW SOME :[L15]
NEUTRAL REACTION: ACTIVITY SEQUENCE, MORE APPROPRIATE FOR EXPERT AUDIENCE :[L15]

She thought that if she were getting a new house, she would like to see how much she is currently "running around" in order to make a more centralized design. She felt she already knew some things though, and felt the rest would be more appropriate for an expert audience.

> I think this would be very helpful for planners or architects, probably not for ordinary people. :[L15]

NEUTRAL REACTION: MEDICATION AND PLACE, ALREADY HAS ROUTINE IN PLACE :[L15]
She doesn't take much medication, so didn't find that the medication-place example applied to her. Her medication regimen is currently very routinized and she uses a common place (in the kitchen) for medications that are temporarily prescribed.

IDEA USE: RETRACING STEPS TO EVOKE MEMORY AND HELP LOCATE MISSING ITEMS :[L15]
IDEA USE: HELPING CAREGIVERS RETRACE STEPS TO LOCATE MISSING ITEMS :[L15]
She feels though that as she gets older, she might like information that would help her locate items. Forgetting itself is troubling, so she would like information that could help her trace what she was doing and "bring back the memory." She also foresaw that having information might help her caregivers as well, if she were incapable.

NEUTRAL REACTION: HIGH POINT/LOW POINT, MOOD IS FAIRLY CONSTANT :[L15]
BENEFIT: EVEN IF MOOD IS CONSTANT NOW, ONE DAY MAY HAVE MOOD SWINGS OR DEPRESSED MOOD :[L15]
BENEFIT: IDENTIFYING WHAT MAKES YOU HAPPY CAN HELP YOU WHEN IT'S DIFFICULT TO MAINTAIN UP MOOD :[L15]
She thought the high point/low point would be good for other people, and maybe one day for her, but not right now because her mood is fairly constant. She noted that mood swings are the kind of thing that could happen to anyone at some point in their life, so thought it might one day be helpful. She specifically thought that it might be helpful to identify things that make her really happy (other than good food, good music, and good movies) that she doesn't know about for later on.

[Activity](0:35:00)
POSITIVE REACTION: ACTIVITY TRACKING :[L15]
She thought that the activity data would be helpful and thought that looking at it on a week-scale would be fine.

CONCERN: INFORMATION CAN BE UNHELPFUL IN SITUATIONS WHERE SCHEDULE CONSTRAINS CHOICES :[L15]
She noted that sometimes there are constraints that limit what people can do at particular times of day (for example, if they work) and that having that information would not be helpful.

IDEA USE: IDENTIFY LITTLE CHANGES THAT CAN BE MADE (GETTING MORE ACTIVITY ON BREAKS) :[L15]
She believes that if change occurs slowly, you will feel better, and that helping people to identify little things they can do (e.g., get more activity on their breaks at work) would be helpful.

[Years](0:38:30)
POSITIVE REACTION: LONG TIME SCALE :[L15]
NEUTRAL REACTION: TIME SPENT COOKING, ALREADY KNOW :[L15]
Her initial reaction to looking at a longer time-scale was that it would be very interesting, but noted that she thought she knew about significant changes, such as with time spent cooking over the past 10 years.

`Big changes I probably know. Little changes, minor ones, I probably don't know. :[L15]`

She knew she cooked less and less as her daughters moved out.

IDEA RELATIONSHIP: SLOWNESS, AGILITY AND AMOUNT OF ACTIVITY, TIME SPENT SITTING DOWN, OVER LONG TERM :[L15]
She thought that her body had become slower and less agile over the last 10 years. She would like to see whether those changes are associated with whether she moved less, sat down more.

IDEA VARIABLE: ABILITY TO RECALL DETAILS OF MOVIES WATCHED IN PREVIOUS WEEKS, TIME TO RECALL :[L15]
She indicated that she was interested in memory changes and notes that there have been some changes in the last 10 years; specifically that it will take her longer to recall specific memories (like what she did last week). She notes that if she watches a movie in the previous week, the details are already faded and she has to concentrate to really recall it and her response to it.

BENEFIT: PROMPTING MEDICAL VISIT (MEMORY) :[L15]
When asked what she would do if she tracked those kinds of changes to memory over time and saw a decline, she indicated she would talk with a doctor and that she would want to know. She indicated that it might help to put it in the context of her friends' cognitive abilities.

PROCEDURE: CONFUSION ABOUT OVERLAPPING DATA SCALES :[L15]
She was confused by the overlapping scales (memory versus time spent cooking) and was advised to uncheck some options to reduce clutter.

POSITIVE REACTION: TIME SPENT WITH FRIENDS OVER TIME :[L15]
IDEA RELATIONSHIP: TIME SPENT WITH FRIENDS AND MOOD, GENERAL HEALTH :[L15]
She knows that social interaction is related with mood so thought that tracking time spent with friends would be good. She noted though that some people can be content without having a lot of social interactions. She thought that social interaction would be related to mood and health change for herself.

IDEA RELATIONSHIP: SHOPPING TRIPS AND MOOD/HEALTH :[L15]
She thought that a good indicator of health change for herself would be whether she was going out, specifically to do necessary shopping.

[Sorting] (0:49:50)
IDEA RELATIONSHIP: BAROMETRIC PRESSURE AND MOOD :[L15]
She believes barometric pressure is related to mood.

She was curious whether she had to say it out loud or if I could see her choices.

BENEFIT: IT DOESN'T HURT TO TRACK (TIME SPENT READING, WATCHING TV, MOVIES) :[L15]
She believes she "kind of knows" how much she is reading, watching TV, etc., but "it doesn't hurt to track"

She resorts by "stress"

Idea relationship: gardening and stress
BENEFIT: MOTIVATION TO DO ACTIVITY (SEEING HOW GARDENING AFFECTS STRESS) :[L15]
She thought there were more specific things that would be associated with stress, and gave as an example, gardening. She knows it's a good thing for her to do, but she has trouble initiating the activity.

ID: [L16]

Date: 8/27/04, 1:00 – 2:00 (65 minutes)
Participant type: Word of mouth
Demographics: Woman, (1946),
Location: Phone (Indiana)
Audio file: DM100026_1

- Shown 6 Displays: Journal, Snoop, Diary, Performance, Place, Years
- In an interesting place in life, recently, and unexpectedly, retired
- Is interested in doing a variety of investigations

Work: Recently retired from a tech-research field, scientific evaluation of chemical technologies
Technologies: Cell phone, digital camera, pda
Technologies: Scale, heart rate monitor (not used), pedometer (not used yet)

[What I monitor] (0:02:00)

CURRENTLY MONITORING: WEIGHT :[L16]

Monitors her weight. Was monitoring her blood pressure, but it is controlled through medication.

BENEFIT: COMBINED INFORMATION; REPLACING INEFFECTIVE RECORD-TRACKING METHODS :[L16]

"I write a lot of things down, but can't keep track of them. I like the idea of everything being electronic." :[L16]

She was keeping an electronic calendar at work, but hasn't done that for home and her non-work-related activities.

[Journal](0:05:55)

CONCERN: DISCIPLINE REQUIRED TO RECORD :[L16]

The participant is excited about tracking, but notes the challenge of being disciplined at recording information.

IDEA VARIABLE: FEELINGS OF ANGER :[L16]
IDEA RELATIONSHIP: BLOOD PRESSURE AND ANGER :[L16]
IDEA USE: INTERVENING WHEN BECOMING ANGRY :[L16]

The participant would like to have a device that reacts to signals that she is getting angry by making her aware and coaching her to keep her temper in check.

"I've often thought that, with respect to behavior modification, if you could have a monitor that would tell you, I don't know how sensitively your emotions could be monitored, but I frequently regret getting angry and if I had something speaking in my ear "[name], your blood pressure is going up, is there something going on around you – listen, don't react." I've often wondered if I would find that helpful." :[L16]

POSITIVE REACTION: SNACKING :[L16]
IDEA USE: NOTIFICATION WHEN CALORIC LIMIT HAS BEEN REACHED DURING THE DAY :[L16]

She would like to be able to track what she eats and get notified in the day to curtail her eating when she has overreached her target caloric intake.

"With respect to weight, to easily say this is what I'm eating and to convert that to calories – well look, you are up to what you have for the day, maybe you need to rethink having that snack." :[L16]

"If you had so many calories and you can either have that candy bar or two apples." :[L16]

CONCERN: ARE THERE HIDDEN REASONS WHY DON'T TRACK ALREADY (OTHER THAN RECORDING IS DIFFICULT) :[L16]

"I don't know if it's just an excuse [that current recording methods are too difficult] that I don't track things like that, or if something was available, whether I would." :[L16]

POSITIVE REACTION: MEMORABLE MOMENTS :[L16]
BENEFIT: RECORDING REINFORCES RECALL :[L16]

"With my age, memory and things like that are not getting any better. I love if I've written it down and can get back to it – oh yeah, that's what happened. Or if one of our sons calls and relates where he's been and what he's been doing, when I relate that information to my husband, I can recall it. If you've written it down or documented, that's easier." :[L16]

BENEFIT: CAPTURING IMPORTANT MOMENTS :[L16]

"Our sons have journaled at summer camp… had it not been required, I don't know if they would have done it. They love the fact that they have written down their experiences in journals and were devastated when they lost one." :[L16]

BENEFIT: BACK-UP FOR MEMORY :[L16]

"I have a tendency to want to have a record of everything. And it seems to be getting stronger, I don't if that's an age thing or what. It intrigues me of why, maybe it's feeling less able to rely on memory and if I have it written down, there it is." :[L16]

BENEFIT: IT'S EASY TO FORGET THINGS YOU THINK YOU WILL REMEMBER (MOVIE TITLES WATCHED) :[L16]

"With respect to movies and books you read, you're like oh, I'm going to remember this – then the next thing you know, you've rented the same movie that you saw <laughs>. Or talking about a book you read and remembering specific parts of it." :[L16]

POSITIVE REACTION: MEDIA :[L16]
IDEA VARIABLE: PREFERENCE RATINGS OF LEISURE EXPERIENCES :[L16]
IDEA INVESTIGATION: WHETHER EMOTIONAL EVENTS DURING THE DAY AFFECT HOW YOU LIKE OR DISLIKE A MEAL, A MOVIE, OR AN EXPERIENCE :[L16]

"I have found it fascinating to try to get at why I found a book or movie positive or whether I didn't enjoy it. I've wondered whether you can separate whatever happens to you, the disagreement you got into that morning, whether that colors everything and that's why you didn't like the movie. Or who you went with, and you were worried that they wouldn't like it, and that's why you weren't just able to enjoy it. I would love to be able to do experiments to see how various apparently not related things can in fact influence how you like and dislike a meal, a movie, an experience." :[L16]

IDEA INVESTIGATION: FACTORS THAT CONTRIBUTED TO A "GOOD DAY" – PRODUCTIVITY, COMMENTS FROM OTHERS, BLOOD SUGAR, OR SOMETHING ELSE :[L16]

"In looking back over a workday – it's like oh, today was really good – well, why was it good? Was it because of some really trivial complement that you were given early in the morning or is it because you accomplished something where you can say look, I discovered how to do this or wrapped something up. What was it about the day or was it just your blood sugar in the morning? I suppose if you are monitoring all kinds of physiological things, you can correlate and have the higher protein load earlier because look at this pattern. I have a feeling that it's much more complicated to do, otherwise you would have figured it out already, but that interests me." :[L16]

IDEA INVESTIGATION: DO BEHAVIORAL/EMOTIONAL EXPLANATIONS FOR EATING APPLY? DO SIMPLE TRICKS TO ABATE HUNGER WORK? :[L16]

“I don’t know if the reminder would make me angry or would be just enough to be helpful. I know various tricks – if you are hungry and have a glass of water, that may pass. Or if you are angry about something and it’s quote/unquote emotional eating, if there is a way to get beyond that. Usually for me, if it is emotional eating, someone reminding me that it is emotional eating is probably not going to help. <laughs> But I would like to try to experiment.” :[L16]

[Snoop](0:18:00)

POSITIVE REACTION: EATING BEHAVIORS :[L16]

“I would like to have that snoop and to do the analysis. See if there is a way that my eating habits might be able to be changed [in a way] that’s not as onerous as a diet. I’m relatively healthy so I can’t think anything that I’m not aware of that I might feel better because of somebody watching.” :[L16]

IDEA INVESTIGATION: WHAT DAILY ROUTINE WILL GIVE SATISFACTION :[L16]

In the past few months, she hasn’t had a routine like she did when she was going to work.

“I’m basically looking for what routine will make me feel good at the end of the day and have been looking for ways to figure that out and it hasn’t come to me yet.” :[L16]

IDEA VARIABLE: TIME SPENT ON ACTIVITIES :[L16]

IDEA RELATIONSHIP: HOW REGIMENTED HER ROUTINE IS AND FEELINGS OF CONTENTMENT AND SATISFACTION :[L16]

She’d like to see what she’s spending her time on and

“…whether or not making [her routine] regimented will in fact result in a greater sense of contentment and satisfaction, what the day has accomplished. That’s due to my sudden recent change.” :[L16]

She had the opportunity to retire early and she chose to do so, a year and a half before she had planned to retire. In some ways she doesn’t feel like she was ready and is trying to decide what to do with her time.

NEGATIVE REACTION: SOCIAL AWARENESS, UNLESS YOU HAD A MOTIVATION TO CHANGE :[L16]

CONCERN: FEELING LIKE BEING CRITICIZED (SOCIAL AWARENESS) :[L16]

CONCERN: IT MAY NOT BE POSSIBLE TO CHANGE THE BEHAVIOR (LINGUISTIC PATTERNS) :[L16]

With respect to social awareness,

“I’d like to hear it, but with respect to whether you feel you are being criticized. Just looking at the repeated use of “you know” in casual conversation – I know that sort of thing would be pointed out and I know it’s very difficult to change that. So I guess it’s a function of whether you feel a motivation to change. Would I know want to know something? I can’t think of anything.” :[L16]

[Diary](0:23:00)

POSITIVE REACTION: DIARY :[L16]

IDEA VARIABLE: TASK FOCUS/COMPLETION (VERSUS DISTRACTION) :[L16]

She thought this would be interesting. When I asked her what we would see if she did this for next few days, she said she thought it would show how often she is using the computer.

“I’m not staying focused on tasks, but rather, oh I want to look this up <laughs> It’s kind of a classic, once you get to the computer, oh there you are surfing and one thing leads to another. It may even be useful things you are discovering as opposed to watching TV or doing some mindless game. However, you are not finishing the task that you started.” :[L16]

“I can get really distracted by doing things like that. I only have a few things I’m going to do during the day and all of the sudden, you haven’t done the two things you were going to do today <laughs>” :[L16]

IDEA USE: YOU HAVE TO HAVE A GOAL IN MIND TO BENEFIT FROM TRACKING :[L16]

She believes you have to have a goal in mind to benefit from tracking data.

> "It would be driven by what do I want to change, what is my goal, and what are the factors that are preventing you from that goal, what could you be doing to do that better. I don't have that in my mind yet. I want to get a part-time job or I want to volunteer. I want to do it by the end of the month – I haven't done that for myself. I guess I feel you have to do that for any of these monitoring things to actually work." :[L16]

IDEA USE: SOMEONE OTHER THAN YOU MAY BE ABLE TO TEASE OUT IDEAS FOR PERSONAL GOALS FROM EXPLORING DATA :[L16]

I ask her if she believes she can use data to determine a personal goal.

> "I don't think I would be able to do that myself… I don't know if I could come up with that would be. I could do it for somebody else." :[L16]

IDEA USE: INFERRED LABELS ON ACTIVITY WOULD BE FINE :[L16]

I ask her about the "inferred" aspect of the collected data, with respect to whether uncertainty would be problematic.

> "Would I still want to see the information? Absolutely." :[L16]

POSITIVE REACTION: REFRIGERATOR EVENTS, TV REMOTE EVENTS :[L16]

IDEA USE: "DISCRETE DATA," SUCH AS REFRIGERATOR OPEN EVENTS CAN BE HELPFUL IN REVEALING PATTERNS OF WHICH YOU ARE NOT AWARE :[L16]

She indicated that the discrete data, such as the refrigerator events and the TV remote events, could be helpful in revealing patterns of which you are not aware.

> "I think the patterns are the things that might bring some sort of insight…." :[L16]

[Performance](0:28:40)

POSITIVE REACTION: MEMORY PERFORMANCE :[L16]

> "I've been intrigued about your ability to remember things, if your mental ability to be monitored in some way – people my age are going oh, I'm forgetting things, I think I'm getting Alzheimer's – isn't there some way to in fact monitor what your mental performance is, repeat back numbers or something, that intrigues me. I don't know if something can in fact do that in a real way." :[L16]

IDEA USE: COGNITIVE PERFORMANCE TESTS SHOULD BE AS OBJECTIVE (CONTROLLED?) AS POSSIBLE :[L16]

I asked how she would feel if it were an explicit memory assessment as opposed to something like a game.

> "I would it prefer to be as objective as possible, I don't know if that's the right word. So no, it wouldn't have to be a game." :[L16]

IDEA VARIABLE: FLEXIBILITY :[L16]

IDEA VARIABLE: STRENGTH :[L16]

IDEA VARIABLE: HAPPINESS :[L16]

IDEA USE: ONE SPOT READING THAT ENCAPSULATES MULTIPLE MEASURES OF PHYSICAL HEALTH (FLEXIBILITY, STRENGTH, HAPPINESS, WEIGHT, ACTIVITY) :[L16]

> "Also like your physical, like you put a thermometer in your mouth and there's your temperature, if you could take your physical, how flexible and how strong and how happy you are. That's broken up into weight and activity." :[L16]

IDEA INVESTIGATION: ARE SEASONAL CHANGES IN MOOD RELATED TO SEASONAL QUALITIES (LIGHT/TEMPERATURE) OR REDUCTION IN ACTIVITY :[L16]

She doesn't have a sense of the variability in her mental abilities or mood. Her husband believes that he has seasonal affective disorder, because he feels down in the wintertime. She notes that he also doesn't get to ride his bicycle as much though, so she questions whether the change in physical activity may be the cause.

> "If there were a way to monitor these things in a more whole body way…" :[L16]

IDEA USE: WOULD FEEL COMFORTABLE TAKING TRACKING INFORMATION TO DOCTOR :[L16]
She could see herself taking tracking information to her doctor. She doesn't take anything right now, but because she had a bilateral mastectomy a few years ago, she goes in for frequent exams and her doctors more closely monitor change. She feels like she is being monitored.

[Place](0:35:40)
POSITIVE REACTION: ACTIVITY SEQUENCES IN SPACE, CURIOSITY :[L16]

> "[she would be interested in seeing this kind of data] only because I have no idea what the pattern would look like and I'm interested for that reason." :[L16]

CONCERN: LACK OF FAMILIARITY WITH METRIC MAKES IT DIFFICULT TO DETERMINE APPLICATIONS (ACTIVITY SEQUENCES IN SPACE) :[L16]
She agreed that it was so unfamiliar, she is not sure what she would do with it. She feels it would be fascinating though

POSITIVE REACTION: HIGH POINTS/LOW POINTS :[L16]
IDEA RELATIONSHIP: MOOD AND PEOPLE INTERACTING WITH, TIME OF DAY, FOOD :[L16]
She did believe the high points/low points example would be useful. I asked her what situational factors she would like to examine with respect to mood.

> "Definitely the people I'm interacting with and time of day to see whether the patterns are associated with it. And food." :[L16]

[Years](0:38:40)
POSITIVE REACTION: YEARS :[L16]
IDEA VARIABLE: ACTIVITY PATTERNS OVER YEARS :[L16]
IDEA RELATIONSHIP: ACTIVITY PATTERNS AND LIFESTYLE (WITH KIDS) :[L16]
IDEA USE: FINDING A SUBSTITUE ACTIIVTY AFTER LIFESTYLE REQUIREMENTS CHANGE (KIDS GROW UP) :[L16]
IDEA RELATIONSHIP: WHO YOU'RE LIVING WITH, WHEN THEY COME HOME, WHEN MEAL TIMES ARE :[L16]
I asked her what she would like to know about what her life was like 10 years ago compared with today.

> "Activity patterns with and without children would be dramatically different <laughs> and I think you don't realize – now that you're delving into this – I have this feeling I'm not as active, but why not? Well, you know when you are dealing with children, I think your activity levels are definitely affected and you can see a difference without them. Similarly who you're living with, when they come home and when meal times are, that could be correlated. I guess it's well look, you're not nearly as active, you are going to have to substitute something in order to be more active, what might that be with respect to your time and other constraints and your enjoyment levels. You can positively affect your body." :[L16]

IDEA VARIABLE: SOCIAL INTERACTION OVER YEARS, HOW MANY TIMES YOU HAVE SOMEONE OVER TO DINNER, GO OUT TO DINNER :[L16]
I asked her to think about the next ten years, what she would like to track.

> "Social interaction, how many times do you have someone over to dinner, go out to dinner, I could see those things really taking a nose dive."

CONCERN: IF YOU THINK YOU KNOW YOURSELF AND DON'T NEED INFORMATION, HAVING INFORMATION WON'T HELP :[L16]

"I think depending on how you receive information about yourself and whether you feel having outside or impartial information about you can be useful. If you think 'I know myself, I don't need any information', that's your preconceived notion, no graph is going to do anything." :[L16]

CONCERN: SOME PEOPLE DON'T WANT TO QUESTION THEIR BELIEFS ABOUT THEMSELVES AND THEIR PERSONAL HEALTH BEHAVIORS :[L16]

She recounts how she has a relative who takes supplements. She is of the opinion that the supplements aren't doing anything and has suggested that the relative try running some experiments (using placebos) to see if there is an effect, but the relative has no interest in doing so.

"It has to do with your attitude toward information. Number 1, do you trust the measurement, do you think it is something real. Like an aura measurement I would have no interest in." :[L16]

[Sorting] (0:44:37)

She would like to track ability to concentrate with respect to aging.

She would like to track ability to recall information, but not stories/jokes

IDEA USE: TRACKING SOMETHING AS A COUPLE OUT OF CONCERN THAT ONE PERSON HAS A PROBLEM (ALCOHOL) :[L16]

"I wonder if my husband is drinking more – I know he is – and I wonder if it is excessive. I would like to track his alcohol drinking and could track mine as well. It would be interesting to see if he would [be willing]." :[L16]

IDEA VARIABLE: SUSCEPTIBILITY TO DISTRACTION :[L16]

She relates awareness of time to susceptibility to distraction and that's of interest to her.

She doesn't think barometric pressure affects her, but thinks it would be fun to find out for sure.

She thinks her blood pressure is under control and doesn't need to track.

She would be interested in knowing how long it takes her to decide what to wear and would "cringe at that"

She would be very interested in commitments

She is less interested in community traffic congestion because she can choose when to go out into it.

She is fascinated with social interaction and would like to track conversation turn taking.

She would like to examine communication with family/friends by medium (phone, email, direct). She would like to know how much time she is spending emailing in particular, because she guesses that it is significant.

She wonders how gullibility would be measured but may be interested

She wanted to measure heart rate with respect to building anger.

IDEA INVESTIGATION: DRUG TRIALS AND MENTAL ABILITY, HOW RELATE TO WORLD :[L16]

She had a hard time convincing doctors to let her go on estrogen during menopause. She had to argue that her quality of life was more important to her than the risk. Her oncologist had her try several psychiatric compounds, unsuccessfully, and she wish had been able to monitor that to see what those medications affected how she relates to the world

> "I would have wanted to measure cognitive ability, the ability to concentrate… something quick that you could do over a week or two while trying a drug." :[L16]

CONCERN: TRACKING BECOMES TOO ATTENTION CONSUMING AND TAKES AWAY FROM GOAL ITSELF (FAMILY KNOWLEDGE) :[L16]

She wanted to know how "how much you know about your friends and family" would be measured and agreed that she would like to track that, but only to the degree that it in itself wasn't becoming too attention consuming.

CONCERN: ABILITY TO RATE SUBJECTIVE QUALITIES LIKE MOOD :[L16]

> "I'm not sure I can do that [rate her mood] very well, but yes I would like it" :[L16]

She would like to explore whether multitasking is beneficial or not.

She would like to track how many people she actually interacted with…

She felt her hearing was starting to get worse and would like to monitor

IDEA RELATIONSHIP: RAISED VOICE AND AFFECT ON OTHER PEOPLE :[L16]

She wanted to track raised voices in terms of an indicator of her own anger. She also noted that a raised voice, even when from excitement, could turn people off, so she would like to correlate voice amplitude with people's perceptions.

She wanted to track when she feels the need to have something to eat and why

> "Spouse's mood, yes! If you can figure that out, let me know." :[L16]

She was interested in general in how her day was "arranged," in terms of wake-sleep cycles and time spent in activities such as cooking.

She doesn't watch much TV and so is not interested in tracking it.

She interpreted "use of space" as having to do with organization, noting they have too much stuff.

Re-sorted by mental sharpness.

ID: [L17]

Date: 6/13/04, 4:45-6:05PM (80 minutes)
Participant type: Friend to researcher
Demographics: Woman, 59
Location: Participant home
Audio file: DM100005

- Shown 6 Displays: Journal, Snoop, Grocery, Daily Rhythm, Place

GENERAL REACTION: POSITIVE ABOUT TRACKING (+) :[L17]

- Interest in daily rhythms and routines
- Interest in spouse's patterns and how each individual affects the other
- Nice example of selecting additional constructs on resort
- Observation about seeing patterns on a larger time scale

Work: Mostly retired, works part-time catering and at a restaurant
Technologies: Uses a cell phone and a digital camera
Tracking devices: Owns a bathroom scale and pedometer

[What I monitor] (not recorded)

CURRENTLY TRACKING: MEMORABLE MOMENTS, TRAVEL, INTENSE TIMES IN LIFE :[L17]

Has kept a personal journal in the past, keeps a travel journal that includes memorable moments with family

CURRENTLY TRACKING: BEFORE-AFTER PHOTOS FOR SENSE OF ACCOMPLISHMENT :[L17]

Recently took a before-after photo of gardening work to get a sense of accomplishment

CURRENTLY TRACKING: WILDLIFE PATTERNS :[L17]

She has been keeping a record of how much sugar she uses to feed the hummingbirds over time as a way of studying how many travel to the area. She wants to start tracking other variables related to wildlife and noted that she recently found out a friend is doing this as well.

IDEA VARIABLE: WHETHER YOU DREAM, COMPLEXITY OR RICHNESS OF DREAMING :[L17]
IDEA RELATIONSHIP: VITAMIN-B SUPPLEMENTS AND SHORT-TERM MEMORY, DREAMING, READING ABILITY :[L17]

After experiencing short-term memory problems during menopause, she started taking vitamin-B supplements. She believes it has made a significant improvement in her memory; she also notes that she remembers her dreams every day now and they are quite vivid, which she wasn't doing. She's always found it difficult to concentrate on reading, but finds it easier now and can read for much longer periods.

[Journal] (0:08:30)

POSITIVE REACTION: PLACES VISITED, WITH FREQUENCY, WASTING TIME? :[L17]

> "I think it would be valuable to know which places I frequent the most, such as I tend to like to go to a lot of grocery stores to pick out the things we like to eat, and I don't have a real clear idea of how often I'm going to the store and if I understood that maybe I could have a better schedule, so I'm not wasting time going to the store, I could use that time in another way." [L17]

IDEA USE: PEOPLE ENCOUNTERED FOR KEEPING TRACK OF CHECKING ON OLDER ADULTS :[L17]

The participant couldn't see how she would use the people encountered, but could imagine other people finding it useful, such as those who are checking on older adults (i.e. knowing when the last time you checked on that person).

NEUTRAL REACTION: ALREADY KNOW SNACKING :[L17]

"Since I've recently changed the way I eat – I think it would be good to be aware of how much you're snacking, I try not to eat between meals too much – but if I thought I was snacking more than I should."

POSITIVE REACTION: MONITORING WATER DRINKING; IDEA USE: COMBINE WITH REMINDER TO DRINK WATER :[L17]

"I really would like to know that, because I do think water drinking is good." She indicated that she would like reminders for water drinking.

POSITIVE REACTION: MEMORABLE MOMENTS FOR RECORD :[L17]

"I definitely like the idea of if I heard a memorable story or an incident that I found fascinating, as something I kept that I could go back in be reminded of it in the future."

IDEA USE: SUGGESTIONS OF WHAT TO READ :[L17]

"The other thing is that if somebody gave me some information that I thought hey – like a suggestion of a book to read or a suggestion to try something they had tried, and I thought it was a good idea, and I went hey, I want to remember that, to remind myself to check on it." (0:14:50)

POSITIVE REACTION: MEDIA SOURCE OF NEWS :[L17]
NEUTRAL REACTION: MEDIA AND POINT IN LIFE :[L17]

"I find that sometimes I get information about a certain vitamin or something and it's so easy to take that information and you think you're going to remember it and you don't. Or you say, there was something about that – did I read it, did I see it on TV?" [L17]

She doesn't think she would be interested in looking at what she was watching or listening to at a certain time period, unless there was a dramatic change.

[Snoop] (0:21:55)

POSITIVE REACTION: DISTRACTION BY EXTERNAL FACTORS :[L17]

She would like to know how often she gets distracted by external events.

POSITIVE REACTION: HOW WELL GIVING VERBAL FEEDBACK TO SPOUSE :[L17]

She would like to know how well she is giving feedback to her spouse, such as the "I'm listening" cues.

POSITIVE REACTION: POSTURE, HUNCHED POSITIONS :[L17]
IDEA RELATIONSHIP: POSTURE AND AGING, POSTURE AND BACK STRENGTH :[L17]

She thought the posture information would be good and noted that it is so easy to not be aware of hunched positions. She's concerned about her posture as she ages; she wants to maintain a strong back.

POSITIVE REACTION: OPPORTUNITIES FOR EXERCISE :[L17]

She liked the "opportunities for exercise" example, noting that she should be walking more and would like to combine exercise, such as lifting weights, with other activities.

[Grocery] (0:26:30) (participant choice)

NEUTRAL REACTION: NATURAL VERSUS ARTIFICIAL, WHETHER ITEMS MEET NUTRITION GOALS :[L17]

She feels that she already knows whether she is buying products with natural versus artificial ingredients and whether her items meet her nutrition goals.

POSITIVE REACTION: LAST TIME PURCHASED :[L17]
IDEA USE: LAST TIME PURCHASED TO REMIND ABOUT INVENTORY :[L17]
"The *last time I purchased* looks like it would be something that would be interesting to me, because it is easy to forget what you have."

POSITIVE REACTION: GROCERY SPENDING, BROKEN DOWN BY SNACKING VERSUS REAL FOOD, AFTER GROCERY TRIP :[L17]
IDEA VARIABLE: ITEMS HAVEN'T PURCHASED IN A WHILE :[L17]
She also liked being able to track spending.

> "I think it would help me to be a little more frugal at times if I needed to be and also let me know, hey you can spend a little more <laughs>. If you have a goal of how much you would like to spend in a month and it could tell you in a printout at the grocery store. I like that it gives you a breakdown of snack food versus real food versus not food. I think that's something that I would really like having." [L17]

> "Could it have a reminder, you usually purchase this item and you haven't purchased it in a while – so you just have an idea, I haven't been buying that – is that something I just don't want to buy any more or have I been meaning to get that." [L17]

IDEA USE: DELIVERY OF INFORMATION ON GROCERY RECEIPT – COULD RUN BACK AND GET SOMETHING OR PUT IT ON THE LIST FOR NEXT TIME :[L17]
She thought the timing of the information, on the grocery receipt, would be helpful (as long as it was in large print).

> "..you could go back and purchase an item if you forgot it or put it on the list for next time." [L17]

[Daily rhythm] (0:32:50)

POSITIVE REACTION: SLEEP PATTERNS :[L17]
IDEA RELATIONSHIP: SLEEP PATTERNS, ACTIVITY, FEELING RESTED :[L17]
She liked the daily rhythm example.

> "I'd liked to know the getting up and going to bed, and what days I feel really rested and what days I don't, so I can see pattern, of when I'm getting rest, and when I'm getting activity. It gives me an idea of what I need to change, if anything." [L17]

POSITIVE REACTION: COMPARING SLEEP PATTERNS WITH SPOUSE :[L17]
IDEA INVESTIGATION: IS YOUR SLEEP PROBLEM AFFECTING SPOUSE, IS THERE A COMMON CAUSE, DOES THE TIME ONE OF YOU GO TO SLEEP AFFECT THE REST OF THE OTHER :[L17]
She liked the idea of comparing her pattern with her spouse's.

> "You know, I'm always interested in how he's [her husband] doing versus how I'm doing. A lot of times you wonder if what's going on with you is affecting the other person. That may be able to tell you something. If you're staying awake at night or not sleeping well, is that affecting the other person, are they having the same problem." [L17]

BENEFIT: IS IT OCCASIONAL OR PATTERN (TROUBLE SLEEPING) :[L17]

> "Whether you had a restful night, if you were able to have that kind of information. How are you feeling each day and how are you sleeping. Is that pattern every day or are you finding just once in a while that you are not sleeping well." [L17]

POSITIVE REACTION: HOURS OUTSIDE THE HOUSE :[L17]
IDEA RELATIONSHIP: HOURS OUTSIDE THE HOUSE AND FEELING WORN OUT/FEELING GROUNDED :[L17]
BENEFIT: DETERMINING TOO MUCH OR TOO LITTLE (TIME OUTSIDE HOME) :[L17]

She thought hours spent outside of the home would be good information.

“It’s easy when you retire to forget to do certain things. Like, I need to get out to be with people more. Or I’m feeling worn out, maybe there’s too much activity out there and I need to stay in and be more grounded.” [L17]

POSITIVE REACTION: EATING TIMES :[L17]
IDEA USE: BALANCING EATING SCHEDULE :[L17]
BENEFIT: SEEING CHAINING OF EVENTS (EATING LUNCH LATE, EATING DINNER LATE, HEART BURN) :[L17]

“I like the idea of being able to be aware of times you’re eating, when you’re eating, kind of what you are doing during the day, when you’re having conversations. I notice I have a tendency to like to eat around 8 o’clock or after, I make a conscious effort to. I have to pay more attention to when I’m eating lunch, because I’m running errands. I often end up eating lunch at 3 o’clock, which I don’t like, I’d rather be eating at 1 to 2, somewhere in there.” [L17]

It affects what time she eats dinner and when she takes her vitamins.

“The other part, is I think I’m waiting too long between breakfast and lunch. With my body, I feel that if things are balanced, when I eat, when I sleep, I feel better physically and mentally.” [L17]

[Commitments] (0:41:05)

NEUTRAL REACTION: COMMITMENTS :[L17]

In the past, she has set up tasks for projects by when they need to get done, once a month, twice a year, etc. and only let’s herself postpone something once. She thinks it is a good idea, but doesn’t do it regularly. She thinks it would good to identify both what you are procrastinating about and what you do get done. She liked the idea that instead of being a constant reminder, it just asked at a prespecified time whether something had been done.

[Place] (0:51:38)

IDEA INVESTIGATION: THE CIRCUMSTANCES (ROOM) WHEN DISTRACTED FROM INTENDED TASK :[L17]

She believes she is good about when she takes medication and vitamins, but would like to know what was going on when she did miss it. She imagined that it could show her what the circumstances were when she got distracted and let time get away from her.

“I believe if I knew the room and the time, I would know what I was doing.” [L17]

“I think it would be interesting to know what distracts me from doing projects. What do you go off and do when you were planning on doing something else.” [L17]

POSITIVE REACTION: ACTIVITY PATTERNS :[L17]
IDEA INVESTIGATION: COMPARING ACTIVITY PATTERNS WITH SPOUSE :[L17]

“I love the knowing the actual activity sequence. I think that is pretty interesting to see how you are using your space. I do several things at once, which drives my husband crazy. It would be very interesting to see my pattern versus his pattern. I think one thing that might help you understand is being a little more accepting of how you each do things – this is my pattern, this is his pattern. We’re so different in the way we do things, that might be why we don’t work well together. We have found that we can both work outside together [gardening, caring for wildlife, doing chores], as long as we don’t do the same thing <laughs>, because our styles are so totally different.” [L17]

BENEFIT: UNDERSTANDING INDIVIDUAL DIFFERENCES :[L17]

“If your patterns were different, it would let you know why you get on each other’s nerves occasionally. I don’t know if it would change anything, but give you understanding.” [L17]

POSITIVE REACTION: LOW POINTS AND HIGH POINTS :[L17]

IDEA RELATIONSHIP: MOOD AND LIGHT AND TEMPERATURE :[L17]

She would like to understand the relationship between her mood and environmental factors, particularly light and temperature.

"Especially if you are finding that you have certain moods, certain days – I think I'd like to understand that. I'd like to understand if there's a pattern there, if I'm affected by light or by temperature. I think those two – I'm suspicious that I have a problem there. Also, what other things that affect mood." [L17]

BENEFIT: RECOGNIZING LONG-STANDING PROBLEM LIKE DEPRESSION :[L17]

The Places example focuses on individual examples of low points and high points. I ask the participant if she prefers looking at individual examples or trends over time.

"I think trends over time would give you a better idea <than specific examples>. I don't think you're aware – I went through a period of time when I was depressed and I didn't realize I was, I think if I had something that said, have you noticed that you are down at this time and this time, if I had had something like that, I think I would have gone in earlier. I thought it was just me and the circumstances of the world." [L17]

[Sorting] (1:02:30)

NEUTRAL REACTION: DON'T THINK DO BEHAVIOR (SNORING) :[L17]

She does not think she snores and therefore does not want to track it.

She needed an explanation of what muscle tone means and decided it might be interesting to know.

She felt that variation from routine and use of space would be interesting.

NEUTRAL REACTION: BEHAVIOR (TV WATCHING) UNDER CONTROL :[L17]

She feels she is careful with how much TV she watches, so doesn't need to track it.

NEUTRAL REACTION: THAT'S NOT WHO I AM (IMPULSIVENESS) :[L17]

She doesn't feel she is an impulsive person.

She doesn't think she has a problem with blood glucose, but would be interesting in knowing that.

She would like to track tea drinking instead of coffee drinking.

IDEA VARIABLE: HOW SPOUSE RATES HER MOOD :[L17]

She would like to see how her husband rates her mood compared with how she rates her mood.

PROCEDURE: ON RESORT, INCLUDES PREVIOUSLY DISMISSED ITEMS :[L17]

When [L17] resorts for "things I would track to understand what affects my feelings of stress," she adds several cards that she did not include in her original "I want to track" pile.

"All of these, I go my god, I just never thought about it." [L17]

NEUTRAL REACTION: VARIABLE (NIGHT VISION) DOESN'T CHANGE :[L17]
IDEA RELATIONSHIP: STRESS AND WHETHER PROBLEMS WITH NIGHT VISION ARE MORE DISCONCERTING :[L17]

She believes her night vision is not good, but does not feel there is any change in it. However, when she resorts, she includes it:

"I know that if I'm having trouble with my night vision, it can stress me out" [L17]

NEGATIVE REACTION: CONCERN ABOUT GETTING OBSESSED (IDLE TIME) :[L17]
IDEA RELATIONSHIP: STRESS AND WHETHER IDLE TIME IS SATISFYING OR DISTURBING :[L17]

Initially, she is concerned that could become obsessed with idle time, because she has a tendency to think that she has to "stay busy". However, she later adds-in idle time because she feels that she's more negatively reactive to idle moments when in a poor mood.

"I can tell my moods, if I'm in a down mood, idle time really is disturbing to me. It does not relieve me, it disturbs me." [L17]

IDEA RELATIONSHIP: STRESS/BEING MOODY AND DIFFICULTY DECIDING WHAT TO WEAR (TIME DECIDING, NUMBER OF CLOTHING CHANGES) :[L17]

IDEA RELATIONSHIP: STRESS AND GETTING TASKS DONE ON TIME :[L17]

She also includes clothing choices, even though she did not originally select it.

"Clothes choice - I can tell when I'm moody, I can try on clothes several times. Awareness of time – if I'm not getting things done on time. If I watch too much TV, that can depress me." [L17]

IDEA RELATIONSHIP: STRESS AND IMPULSIVITY – PARTICULARLY STRESS AND IMPULSIVE TRIPS TO GROCERY STORE :[L17]

She does not consider herself an impulsive person, but associates impulsivity with stress.

"Trips to the grocery, a lot of times I enjoy it, but then if I'm thinking oh, I've got to go here and here and here, I set that up for myself and it can stress me out." [L17]

[Years] (0:29:40) second file

INVESTIGATION: PRESENCE OF ACTIVITIES OVER YEARS TIMESPAN :[L17]

"I've done this actually – I thought ten years ago, I was 49. What was I doing then? Can I think about what kind of changes I've made and that's not real clear, about what is still in my life and what has gone away. In my mind, it's like I'm doing basically the same things that I was doing then, but I bet if I started looking at it, that there were a lot of things that I used to do that I'm not doing now. Re-examining – is it a good thing that is no longer in my life? Or would I like to try doing that again? Add more activity to my life. I really think if I looked ten years ahead, started recording, I think what would happen is that you would end up adding more stuff to your life and maybe get rid of stuff in your life, if you were actually able to track it." [L17]

BENEFIT: EVALUATION OF WHETHER SPENDING TIME ON WHAT IS IMPORTANT, ADDING IMPORTANT ACTIVITIES, REMOVING UNIMPORTANT ACTIVITIES :[L17]

"I think you would decide what things really are not that important and you're spending way too much time doing it and you'd really like to be doing something else. Especially as you get older, you think more that these are important years and would I really want to spend my time doing that, or something else?" [L17]

IDEA VARIABLE: JOB SATISFACTION :[L17]

IDEA VARIABLE: TIME SPENT ON CHORES :[L17]

"Doing a job that I really don't get into and maybe I need a change there. Am I spending too much time doing chores? Is that so important to me? Should I be doing things I always wanted to do? I could see how something like that would give you the opportunity to change some things in your life." [L17]

(0:36:00)

BENEFIT: SENSE OF CONTROL OVER LIFE, ABILITY TO AFFECT DIRECTION OF LIFE :[L17]

"People are so busy doing their lives that they are not looking at their life. If you had the opportunity to track, you might make some big changes in your life, because hey, life is short…. I think it could give you a lot more control in your life." [L17]

(0:38:00)

BENEFIT: MAKING IT EASIER TO SEARCH MEMORY OF EXPERIENCES :[L17]

BENEFIT: TAKING EMOTION OUT OF REVIEWING LIFE EVENTS TO PERMIT EVALUATION :[L17]

“For a while I was journaling, but to sit down and wade through all that, reading everything I’ve said from that period – if it was into a place of, where you are taking a little bit of the emotion out of it, but could see what you were doing, which brings up the memory.” [L17]

ID: [L18]

Date: 6/21/04, 1:56-3:10PM (74 minutes)
Participant type: Friend to researcher
Demographics: Man, 59
Location: By phone (Colorado)
Audio file: DM100026

- Shown 4 Displays: Journal, Snoop, Email, TV

GENERAL REACTION: INTEREST IN SOME INVESTIGATIONS (-/+) :[L18]

- Act of recording strengthens memory
- Interested in investigating recommendations of doctors
- Interested in tracking time spent on favorite activities

Work: Retired, freelance photographer
Tracking devices: Owns a scale, his wife and daughter own pedometers
Technologies: Owns a digital camera, cell phone

“Everything I track is in my head or in email or in my photo file.”

IDEA VARIABLE: WILDLIFE :[L18]

[L18] keeps a record on the computer he calls “first of the year, flora and fauna” which he uses to track the seasonal changes in animals and plants around his home.

IDEA RELATIONSHIP: HOURS PUT INTO A PROJECT VERSUS OUTCOMES :[L18]

He has his email broken down by category and mainly reviews the folders when he’s looking for a particular email, but occasionally uses it as a spot check on how successful has been on a particular project.

“I sometimes, when I’m looking over my environmental work [emails], I’m glancing to try to get a feel for how many of the damn things I wrote, to whom, and over what period of time, because occasionally I try to assess how much work and how many hours I’ve put into it and what has been accomplished.” :[L18]

[Journal] (0:10:58)

CURRENTLY TRACKING: MOVIE RECOMMENDATIONS :[L18]

He didn’t find any of the examples problematic, but thought the media may be least interesting. However, he noted that he has a “movie recommendations” email folder.

POSITIVE REACTION: MEDIA, CURRENT EVENTS :[L18]

IDEA VARIABLE: PARTICIPATION IN POLITICAL ACTIVISM :[L18]

He would like to add current events, such as those related to the war, politics, and elections, to what is tracked, particularly with regard to events that he participated in that contributed to something happening, such as someone getting elected.

IDEA RELATIONSHIP: SNACKING AND WEIGHT :[L18]

When asked what he would do with tracking information such as is available in the examples, [L18] offers,

“Snacking. If I found out by weighing myself that I was gaining weight and couldn’t for the life of me figure out why, I could go back and look at how much I was snacking, and that would put it into focus as to why I’m 5 pounds higher.” :[L18]

BENEFIT: INVESTIGATING THEORIES SUGGESTED BY MEDICAL PROFESSIONAL :[L18]

With some further questioning, [L18] admits that he might not see himself personally tracking snacking. However, he counters that if a problem came up, he would like to be able go back and investigate.

“Water drinking, let’s say I wasn’t feeling good and my doctor asked how much water I’m drinking, gee, I don’t know, I drink water and I drink ginger ale, but more ginger ale than water. But then I could go back and say well, that’s what I think is going on, I could go back and look at it.” :[L18]

BENEFIT: ACT OF RECORDING HELPS STRENGTHEN MEMORY :[L18]

“The other thing is, the act of writing things further etches it in my brain. Actually, a way of making it a stronger memory for me. You can either state things 3 times or write them once.” :[L18]

[Snoop] (0:18:50)

CONCERN: HOW TO EVALUATE THE ACCURACY/PERSPECTIVE OF THE SOURCE (SNOOP) :[L18]
CONCERN: OBSERVATIONS CAN FEEL LIKE CRITICISM :[L18]

[L18]’s first reaction to the Snoop scenario is to comment that he does not like criticism. He asks where the information would come from and whether you could trust them.

POSITIVE REACTION: EATING BEHAVIORS :[L18]
POSITIVE REACTION: OPPORTUNITIES FOR EXERCISE :[L18]
POSITIVE REACTION: POSTURE :[L18]
POSITIVE REACTION: WORK HABITS :[L18]
NEGATIVE REACTION: SOCIAL AWARENESS :[L18]
CONCERN: TOO SET IN WAYS TO CHAGE :[L18]
CONCERN: DON’T WANT TO THREATEN WHERE HAPPY WITH SELF :[L18]

“I think all of them are valid and good to have awareness on. Social awareness would be the most difficult to receive. I’m too set in my ways and happy with my own social deal.” :[L18]

He’d like to specify from whom he was getting advice or observations for each type of information.

“I wouldn’t want just anybody [giving this feedback]. If you’re talking about my wife or my daughter, that’s one thing, but if you’re talking about open-ended, I wouldn’t want open-ended advice from people I don’t know or don’t trust.” :[L18]

[Email] (0:30:15)

IDEA USE: GROUP STATISTICS FOR A GROUP WITH WHICH YOU ALIGN YOURSELF, BUT TO WHICH YOU DON’T FORMALLY BELONG :[L18]
IDEA USE: OPINIONS FROM MEMBERS OF SPORT-CENTRIC GROUP ON WHERE/HOW TO DO SPORT :[L18]

[L18] first indicated that the group statistics wouldn’t be of interest because he doesn’t belong to a group. When I suggested that they may not be people he knows personally, he suggested fly-fishers as a group of people about which he would like statistics,

“If I knew fly-fishers go to monument lake and on average get two trouts per trip, that would be an example. Maybe if they were not organized groups that you know right now.” :[L18]

POSITIVE REACTION: OUTDOOR CONDITIONS (FISHING) :[L18]
POSITIVE REACTION: NEWS RELATED TO PHYSICAL CONDITIONS :[L18]
POSITIVE REACTION: NEWS RELATED TO FOOD PURCHASED/EATEN :[L18]
POSITIVE REACTION: NEWS RELATED TO CURRENT ACTIVITIES :[L18]

He liked the other examples.

[TV watching] (0:38:15)

[L18] noticed that mood was low when the couple was talking more and found it amusing (it was designed that the couple talks more when watching TV, but watching TV lowers their mood – so it's interesting that [L18] saw another possible relationship here).

[L18] also questioned whether heart rate would go up or down while watching TV.

NEGATIVE REACTION: HEART RATE (NOT NEEDED YET) :[L18]

He thought it was all pretty interesting and would like to track it, but would not be interested in heart rate at this time of his life (maybe as he gets older).

POSITIVE REACTION: TV AND MOOD :[L18]

He would like to review it on occasion, if he became concerned about his mood and wanted to experiment with TV watching time to see if that would help.

IDEA USE: SHORT INVESTIGATION TRIGGERED BY CONCERN ABOUT MOOD :[L18]
BENEFIT: ENCOURAGING TRIAL AND ERROR PROBLEM SOLVING APPROACH :[L18]

> "Probably intermittently, short periods of time, meaning on occasion, not over an extended period of time. If I found that I was shocked by how much TV I'm watching and what my moods were, I might try to cut down on TV and see if it changed later. Comparisons I think would be good." :[L18]

[Sorting] (1:04:15)

IDEA VARIABLE: TIME SPENT ON OUTDOOR ACTIVITIES :[L18]

He would like to track coffee and alcohol drinking, email sent/received, laughter, number of digital photos taken, snacking, TV watching. He would like to add outdoor activities – time spent and how you spent it, gardening, fishing, feeding birds or tending to wildlife, and writing to officials regarding environmental causes.

INVESTIGATION: SPENDING TIME DOING THE THINGS YOU LOVE :[L18]

He believes that it is important for people to do the things they love to do to affect their health positively. He notes that people commonly put these things "on the backburner" but if they could see how much they affect their mood, they would make time for them.

> "If you could make it flexible, so people could type in the things you love to do inside the house, now make a list of things you like to do outside the house, now can you see that you might want to track how often you do them and what you're moods are. … visiting places, going to restaurants – what are the things you *love* to do? Would you like to track them?" :[L18]

IDEA RELATIONSHIP: MENTAL SHARPNESS AND TIME SPENT ON ACTIVITIES THAT YOU LOVE TO DO :[L18]

With regard to tracking memory and mental sharpness, he first believes people should pay attention to mood and whether they are doing the stimulating activities that they love to do, such as being involved in causes, reading books, watching movies.

BENEFIT: PUTTING YOURSELF IN THE CONTEXT OF YOUR AGE GROUP (MEMORY) :[L18]
BENEFIT: CHANCE TO TRY TO CURE DISEASE (ALZHEIMER'S) :[L18]

> "At a certain point it would be depressing but it could lend you to say, gee I guess I do have a memory problem… I think comparisons would come in real handy with memory, because all 50-somethings talk about memory loss, short term memory loss, so I think there what would be really helpful is seeing your peer group, and you would take great solace in seeing that you are where about where your peer group is. If you were worse, as awful as that would be, that would tell you you need to go in and talk with your doctor, and say it's beginning to worry me that I have Alzheimer's. I think it's a valuable thing. Do

most people want to know? No. Do most people want to know they have cancer? No…. but how else do you begin to cure it." :[L18]

Resorted according to mental sharpness

ID: [L19]

Date: 8/27/04, 7:30 – 8:30 (60 minutes)
Participant type: Word of mouth
Demographics: Woman, age 61,
Location: Phone (Pennsylvania)
Audio file: DM100029, 30

- Shown 5 Displays: Journal, Snoop, TV, Mirror, Years

Work: Retired, taking care of husband debilitated by a stroke and Parkinson's; adult children
Technologies: Cell phone, digital camera
Tracking devices: Scale, pedometer, blood pressure/pulse monitor.

[What I monitor] (0:03:05)

CURRENTLY MONITORING: STEPS (DID IT FOR A MONTH THIS YEAR) :[L19]
IDEA USE: SHORT INVESTIGATION JUST TO GET AN IDEA OF YOUR TYPICAL BEHAVIOR (STEPS) – MAYBE TO DETERMINE WHETHER A PARTICULAR EXERCISE APPROACH WOULD BE FEASIBLE/PROMISING :[L19]
She got a pedometer a few months back and used it consistently for a month to get a reference of how much she "wasn't moving." She was aiming for 10,000 a day and found that most of hers averaged between 6000 and 8000. Occasionally she made it to the goal.

BENEFIT: MORE ACCURATE THAN READINGS IN CLINIC :[L19]
She's tracking blood pressure at home because she's had higher blood pressure for years and her doctor suggested that she may just have higher blood pressure at the clinic and recommended that she monitor it at home.

CURRENTLY TRACKING: BLOOD PRESSURE :[L19]
She keeps a journal and records her blood pressure and the steps, when she was using the pedometer.

[Journal](0:00:30)(second file)

POSITIVE REACTION: SNACKING :[L19]
She likes snacking and notes that she had recorded the number of calories eaten in a day in the past.

POSITIVE REACTION: WATER DRINKING :[L19]
POSITIVE REACTION: MEMORABLE MOMENTS :[L19]
She also likes water drinking and memorable moments.

She regrets not keeping a journal and would like to have a record of things that happen throughout the day that she would like to remember.

IDEA USE: MONITORING TO MAKE SURE A GOAL IS MET :[L19]
She tries to meet the 8-glasses a day of water goal and would like something that just monitored that she was meeting that goal.

NEUTRAL REACTION: PLACES VISITED, SIMPLE LIFE, HARD TO RELATE TO :[L19]
NEUTRAL REACTION: PEOPLE ENCOUNTERED, SIMPLE LIFE, HARD TO RELATE TO :[L19]
It's hard for her to relate to the places visited and people encountered because she doesn't get out that much day-to-day, as she cares for her husband.

[Snoop](0:03:40)
She initially laughed and said she would be afraid to see what they said, but that it would be interesting.

CONCERN: DIFFICULT TO BEHAVE NATURALLY WHEN YOU KNOW YOU ARE BEING WATCHED :[L19]
She thinks that if there were an actual person following her, it would be difficult to behave naturally and that she would be on her best behavior.

POSITIVE REACTION: EATING BEHAVIORS :[L19]
POSITIVE REACTION: POSTURE :[L19]
If she could be natural, she thought they all looked interesting. I asked her what they would notice if they followed her around for the next few weeks. She thought that they would notice her snacking, because she feels that is something she does without really realizing it. She tends to get a lot of upper back aches and think they may be related to tightening up her shoulders. She would like to see when she's actually do that.

POSITIVE REACTION: OPPORTUNITIES FOR EXERCISE :[L19]
She does a fair amount of exercise in the house just running up and down the stairs and thinks it's enough, but would like to see if it actually is.

NEUTRAL REACTION: SOCIAL AWARENESS, WOULDN'T SERVE A PURPOSE :[L19]
NEUTRAL REACTION: WORK HABITS, LIFE IS STRUCTURED AROUND CARE FOR ANOTHER PERSON, "DOES THINGS AS THEY NEED TO BE DONE" :[L19]
I asked her about the second two examples. She thought she would definitely be more interested in the top three than these. She doesn't know what purpose the social awareness would serve for her. She feels her work throughout the day is structured around her husband's needs, so she "does things as they need to be done."

[TV](0:09:40)
POSITIVE REACTION: TV WATCHING :[L19]
CONCERN: DIFFERENTIATING BEHAVIOR OF FAMILY MEMBERS (TV WATCHING) :[L19]
She notes that the TV is on all the time because her husband watches it, but she would like to know how much time she's actively watching.

She asked for additional explanation, particularly about the mood rating.

She thought all of them would be interesting to her and could see herself tracking them over time.

Her husband has impaired speech output, so they don't have conventional conversations, but she noted that you could track how often she is speaking to him.

POSITIVE REACTION: TIME SPENT OUTSIDE :[L19]
POSITIVE REACTION: TIME SPENT IN CONVERSATION WITH SPOUSE :[L19]
POSITIVE REACTION: MOOD :[L19]
She could imagine wanting to improve variables like time spent outside and time spent in conversation with spouse.

BENEFIT: CONSIDER VARIABLES THAT YOU DON'T ORDINARILY THINK ABOUT :[L19]

She feels like she has a good idea of TV watching, but hasn't thought much about time spent outside or mood.

[Mirror](0:14:15)

POSITIVE REACTION: INFORMATION DISPLAYED ON MIRROR :[L19]
POSITIVE REACTION: HYGIENE TIMINGS, COMPARED TO GOAL :[L19]
POSITIVE REACTION: FLOSSING :[L19]
She thought the idea of having information about her hygiene routine displayed on the bathroom mirror would be clever and liked it.

She follows a daily flossing routine about 50% of the time.

She says that she knows you are supposed to brush your teeth for the length of some song, but can't remember what it was. She likes the idea of being able to track that information in comparison to a goal.

I asked her about the "conditions when you're more likely to do something" idea.

POSITIVE REACTIONS: CONDITIONS THAT COINCIDE WITH DOING OR NOT DOING A TASK (FLOSSING) :[L19]
"It would be interesting to know why you didn't do it certain days." She noted that time she started getting ready for sleep was probably a factor for her and she would like to investigate it.

POSITIVE REACTION (MILD): REFLECTION, CURIOUS :[L19]
I asked her about the reflection example. It was more of a curiosity for her, what she looked like, what she was wearing, and what she was planning to do that day, but didn't think it would be particularly valuable.

POSITIVE REACTION: ACTIVITY FROM PREVIOUS DAY :[L19]
IDEA USE: ACTIVITY FROM PREVIOUS DAY TO SET A NEW GOAL (STEPS) :[L19]
IDEA USE: ACTIVITY FROM PREVIOUS DAY TO REMIND ABOUT CONTINUING PROJECTS :[L19]
I asked her about the final three examples. She was just interested in the activity from the previous day. She noted that she might try increasing the number of steps that she had walked the previous day or notice a project she had been working on to try continuing it. She thought it would serve as a helpful reminder system.

[Years](0:21:15)

CONCERN: TENDENCY (PHILOSOPHY?) NOT TO LOOK BACK ON THINGS IN THE PAST :[L19]
She couldn't think of anything that she would like to have tracked in the past 10 years, because she does not look back on things very often – she doesn't have a need and tries to stay focused on the future.
BENEFIT: HELPING RECALL DAY TO DAY LIFE FROM MANY YEARS AGO :[L19]
IDEA USE: REMEMBER WHAT A DAY WAS LIKE IN THE PAST :[L19]

> "Actually, going back farther to say 20 years ago, I have trouble remembering what a day was like then, so maybe in that respect something like this would serve a purpose. I always felt it was because I was so busy, when the kids were all home, and I really have a hard time remembering a period of years in there what day to day was like." :[L19]

IDEA USE: TRACKING OVER SHORTER TIME PERIODS :[L19]
I ask her about tracking over the next ten years. She couldn't think of anything initially.

> "I would be more be more interested in tracking things in a shorter period of time, like day to day or week to week, but not in the space of 10 years." :[L19]

NEUTRAL REACTION: SHORT-TERM MEMORY OVER TIME :[L19]
She doesn't feel she has a problem with memory and so wouldn't be interested in tracking it, even over a shorter time scale.

[Sorting] (0:26:20)

IDEA RELATIONSHIP: BAROMETRIC PRESSURE AND GOOD DAYS/BAD DAYS FOR IMPAIRED INDIVIDUAL :[L19]
She wanted to track barometric pressure because it seems to affect what kind of day her husband has
She doesn't drink coffee/tea
She doesn't think she is very gullible
She's been told she has a problem with snoring
She thinks she probably watches too much TV

She resorts by eating choices

She notes that her snacks are usually outside the refrigerator.

IDEA RELATIONSHIP: SOCIAL INTERACTION WITH FAMILY AND MINDLESS EATING :[L19]
Conversation turn-taking…
"When there are more people around, a lot of times you can mindlessly eat and snack, sitting at the table with family, not thinking about what you are eating."
IDEA RELATIONSHIP: CLOTHING CHOICES AND WEIGHT :[L19]

I asked her what she would like to track with respect to her husband.
IDEA RELATIONSHIP: WHAT KIND OF DAY AN IMPAIRED FAMILY MEMBER HAS IMPACTS PRIMARY CAREGIVER'S DAY :[L19]
IDEA VARIABLE: MOBILITY, SPEECH, MENTAL ACUITY OF FAMILY MEMBER WHO HAS HAD STROKE :[L19]
She would like to track his mobility, because it varies so much. She noticed that the kind of day he is having determines the kind of day she has. His eating choices are usually good because she prepares the food for him. She wanted to track his speech from day to day and his mental acuity.

BENEFIT: THERE IS A LOT OF VARIABILITY IN CHRONIC COMPLEX CONDITIONS, SO THERE NEEDS TO BE A WAY TO DESCRIBE THAT :[L19]
"There is so much about his problems that vary from day to day, so if there was some way that could be tracked, it would really be of interest." :[L19]

BENEFIT: DIFFICULT TO DETERMINE HOW GOOD OR BAD DAY IS RELATIVE TO PAST :[L19]
"You do tend to forget from day to day, is this a bad day, or is this a day that is worse than it has ever been." :[L19]

She would like to track how many times he walks from his chair to the kitchen and to the bathroom. She hasn't tried a pedometer with him, but was going to try it the following weekend.
"I'll have to try that some time, I never thought about that." :[L19]

She would take information about his mobility, speech, and mental acuity to her doctors.
BENEFIT: INFORMATION FOR COMMUNICATION WITH DOCTORS, DOCTORS RELY ON CAREGIVERS FOR INFORMATION ABOUT PEOPLE WITH CHRONIC CONDITIONS :[L19]
"They rely on what I tell them. That's the only way these kinds of conditions can be tracked, observing them from day to day." :[L19]

ID: [L21]

Date: 8/24/04, 1:30 – 2:30 (60 minutes)
Participant type: Referred by other participant
Demographics: Woman, age 66
Location: House_n
Audio file: DM100019_1

- Shown 5 Displays: Snoop, Mirror, Daily Rhythm, Activity, Years
- Initially negative reaction to tracking, felt the information was not needed
- Recognized value of having baseline data, but just wants passive monitoring

Work: Administrative
Technologies: cell phone only when traveling, no digital camera
Devices: bathroom scale, thought about getting a pedometer

[What I monitor] (0:02:30)
CURRENTLY TRACKING: GARDENING (JOURNAL) :[L21]
CURRENTLY TRACKING: WHAT READING (JOURNAL) :[L21]
CURRENTLY TRACKING: INTENSE TIMES IN LIFE (JOURNAL) :[L21]
BENEFIT: AS A WAY OF DEALING WITH ANXIETY OF DISEASE TREATMENT :[L21]
She keeps several journals, but not on a daily basis. She keeps a journal of her reading, writing a summary paragraph and title for the books that she reads. She keeps a garden journal that includes what she plants and how they do. When she travels, she keeps a record of the things she does and the things she might want to go back and see. She also has a personal reflection journal, which she adds to under intense life circumstances. She had cancer a few years ago and recorded everything that happened to her, how she was feeling, what the doctor said and what her questions were, "as a way of dealing with the anxiety of the cancer and the treatment." She's also written in this personal journal when she moved.

IDEA USE: RECORDS THAT BECOME A REFERENCE FOR HOW TO DO SOMETHING (GARDENING) :[L21]
Her entries are freeform; she just dates them. She does review them – "they become a reference."

Every spring she reviews her garden journal and looks for plants that have lasted well. She doesn't believe there is a lot of difference, however, in how she gardens throughout the journal period of 6 years.

IDEA USE: JOG MEMORY (READING JOURNAL) :[L21]
She uses the reading journal to jog her memory about what she's read and whether she liked a particular author.

She weighs herself a couple times of week at the same time and is just making sure she stays within a particular range.

IDEA USE: CORRESPONDENCE TO MONITOR RELATIONSHIPS :[L21]
She keeps correspondence to and from friends that she may review for suggestions they have made (e.g., what to read) or to reminder herself of events in their lives. She keeps them to "monitor her relationships."

She keeps financial records.

She considers herself a Ludite, giving as examples the fact that she had to be convinced her by her children to get an answering machine, that she never owned a VCR (but now owns a DVD), and that she doesn't use a cell-phone regularly.

[Snoop](0:12:40)

NEGATIVE REACTION: SNOOP EXAMPLES :[L21]

BENEFIT: IF YOU WANTED TO MAKE CHANGE, FOR EXAMPLE TO DIET :[L21]

"I can't imagine why you would want to do this - I suppose I would imagine if you were concerned about your diet or wanted to lose or gain weight, maybe it would be helpful to have insight. I suppose this kind of thing would be useful if you wanted to change your behavior and if you weren't yet aware that you needed to change your behavior, this would not be useful." :[L21]

CONCERN: IT'S BETTER TO GO TO A PROFESSIONAL EXPERT OR TO GET SOCIAL SUPPORT :[L21]

She noted that you generally go to a therapist or nutritionist to address behavior change. Because this resource exists and because social support/responsibility is so powerful, she does not believe that something like this would be useful.

"These kind of behavior change things require a system that helps you to stay on track, if you are in some kind of community or with some kind of authority, for most of us, I don't think I'm unusual, to do it only internally is hard. You're not as likely to do it. So I'm not sure these things would be particularly useful. To me." :[L21]

CONCERN: IF YOU HAVEN'T ALREADY RECOGNIZED A NEED TO CHANGE, THEN WHATEVER YOUR BEHAVIOR IS MAY BE OKAY FOR YOUR LIFE (SOCIAL AWARENESS) :[L21]

"For many of these things, there is a gap between self-awareness and what can be observed. Unless you want to affect change or you feel something is wrong… like social awareness… you may annoy the heck out of people, but you don't feel anything is wrong… you have plenty of friends, you are doing social things as frequently as you need to, so you don't feel a need to change. If you feel you need to make a change, some sort of analysis would be useful." :[L21]

She notes that if you did want to make a change, you could monitor something (such as what you are really eating) and then take it to a professional (nutritionist).

CONCERN: TOO INTROPSECTIVE :[L21]

"I don't need that kind of self-introspection."

I asked her if there was anything she has tried to change in recent years. She has tried to get more exercise by going to a health club. She has discontinued going and admits that she needs to motivate herself to go again.

CONCERN: ALREADY KNOW WHAT THE PROBLEM IS, JUST NEED MOTIVATION :[L21]

"I'm not sure if these observations would help. I know I need to go and I need a regular regimen of exercise… I don't need some kind of monitoring tool to tell me that." :[L21]

CONCERN: IF DOING BETTER THAN EXPECTED, MAY BE DE-MOTIVATING (EXERCISE) :[L21]

"I may get more exercise than I think I do and if I had a monitoring tool might be less motivated to go <laughs>." :[L21]

[Mirror](0:21:30)

NEUTRAL REACTION: HYGIENE, ALREADY HAVE SOLID ROUTINE IN PLACE :[L21]

CONCERN: DOCTORS ALREADY SUPPLY SUFFICIENT INFORMATION ABOUT WHEN A CHANGE IS NEEDED (TOOTHCARE) :[L21]

She tells me about how a few years ago her gums were bleeding and her dentist told her what she needed to do and had her come back in repeatedly. As a result of this experience, she developed a thorough tooth care routine that includes flossing every day that she does without fault.

CONCERN: TRACKING IS REDUNDANT IF YOUR MEMORY IS INTACT (MIRROR EXAMPLES) :[L21]

She couldn't think of anything that she is tried to get more regularly.

> "It is interesting to me that you and the folks are working with had the idea that people would like to have this information. It's sort of – I don't know, maybe if I were getting older and I was having some kind of problem with my memory, it might be a good thing to have something on the bathroom mirror that would say, oops, you didn't do your teeth yet." :[L21]

She wants to age-in-place and could see this as something relevant to someone with memory problems. She couldn't see the value of it when you are healthy and have a routine. She thinks the main thing she needs now is something that eggs her on to call her trainer or go to the gym. She recounts a story about an older relative who was not getting enough electrolytes and notes that a monitoring system could have helped identify that this person wasn't eating right.

CONCERN: ANNOYING :[L21]

> "I would find it a little annoying now." :[L21]

> "I assume you would set these up and say this is the diet you want to stick to… I could see a usefulness under circumstances like that. I would sure want to be able to turn it off <laughs>" :[L21]

NEUTRAL REACTION: REFLECTION, COULDN'T SEE HOW USEFUL :[L21]

She couldn't see how the reflection example would be useful.

NEUTRAL REACTION: ACTIVITY/PEOPLE ENCOUNTERED FROM PREVIOUS DAY – ONLY USEFUL FOR PEOPLE WITH ALZHEIMER'S :[L21]

She thought that the final three examples would mainly be useful for people with Alzheimer's.

She has a calendar with her schedule for the day and that primes her for what she needs to do that day. She doesn't feel she needs more than that.

[Daily Rhythm](0:31:20)

NEUTRAL REACTION: DAILY RHYTHM, NO PERSONAL APPLICATION (ONLY FOR RESEARCH) :[L21]

She thought that the daily rhythm information was relevant for research, but not for personal use.

CONCERN: TOO MUCH STRUCTURE IMPOSED ON FREE TIME :[L21]

IDEA USE: SHORT TIMESCALES ARE REDUNDANT (WEEK, COMMITMENTS) :[L21]

She has a regular schedule and relishes flexible days. She keeps a calendar that also serves as a record. The time-scale of the week seemed particularly redundant for memory.

[Activity](0:34:20)

NEGATIVE REACTION: ACTIVITY INFORMATION :[L21]

CONCERN: INFORMATION OVERLOAD :[L21]

She feels this would be useful if she was trying to make a change, but ordinarily wouldn't need this information. She is concerned this would be information overload.

> "If you are not going to do something with it, it is just data." :[L21]

BENEFIT: PASSIVE MONITORING MAY IDENTIFY THAT A CHANGE IS NEEDED BEFORE PERSON BECOMES AWARE :[L21]

BENEFIT: BASELINE BY WHICH TO IDENTIFY SIGNIFICANT CHANGE :[L21]

She acknowledges that passive monitoring might identify that a change is needed or be used for the purposes of a baseline by which to identify significant change (she brought up these ideas independently).

[Years](0:37:50)

IDEA VARIABLE: ACTIVITY LEVELS OVER YEARS :[L21]

BENEFIT: IDENTIFICATION OF ACCUMULATION OF SUBTLE CHANGES :[L21]

IDEA USE: CHANGE TO STRONG ROUTINES (FLOSSING, BRUSHING TEETH) AFTER YEARS OF CONSTANCY :[L21]

She thought that activity levels would be something she would want to monitor over the next 10 years for detection of significant change.

> "Some kind of monitor that would help remind you that you are making these subtle changes that you are not self-aware as you might be – activity level, or any of these behaviors now that you have triggered my thinking here. For years I've brushed my teeth and flossed and if all the sudden I started skipping days, might not be a bad idea. As you age, what are the things that you now do in a kind of routine way that make you a healthy person, that you might fail to do and need some sort of trigger to say, you're not keeping up." :[L21]

IDEA USE: DON'T USE TRACKING TOOLS TO UNDERSTAND LIFE RIGHT NOW, BUT TO MONITOR AND ANTICIPATE CHANGE AS AGING, AS EARLY WARNING TOOL :[L21]

She thinks of herself as being very healthy, aside from having had cancer. She does agree that monitoring over time could be useful for detecting a major health change.

> "Not using it as a tool for understanding my life right now, but monitor and anticipate change as I'm aging, as an early warning, to go to a doctor. Why are you not walking as much. I could see it as a tool as I'm getting older that would remind me of change. Not what I did today, but the last six months. That would be more useful than did you floss today – but you've flossed every day for the last 12 years and in the last month, you've skipped 5 days, so what's going on." :[L21]

IDEA VARIABLE: WHETHER YOU ARE SCHEDULING ACTIVITIES :[L21]

IDEA VARIABLE: TIME SPENT OUTSIDE THE HOUSE OVER YEARS :[L21]

IDEA USE: USER CAN EXPLAIN SUDDEN CHANGES :[L21]

She believes that general activity level would be the most informative for her personal health changes. She notes that whether you're scheduling things and how much time you are spending outside the home would be relevant for her, particularly once she is in retirement.

> "I know what I did this week and last week, but I won't know how that compares to the activity level of 6 months ago or a year ago. I would be more interested in having monitoring that happened every day, some sort of intervention that would say when there was change. It could say oh, you're suddenly hyperactive what's going on? My granddaughter's getting married, so don't worry about that." :[L21]

IDEA USE: USER SET EXPECTATIONS FOR BEHAVIOR, IN CASE YOU ARE INTENDING TO CHANGE (FITNESS TRAINING) :[L21]

> "I would imagine that with these tools you would have the option of telling them about what the expectations were, so if you decided to change your behavior, if you decided that you were going to run a marathon, you could program that." :[L21]

Her general conclusion was that these tools could be helpful if you were trying to make a specific behavior change and needed help or if you wanted to monitor change over a long timer period, but not for day-to-day tracking.

[Sorting] (0:49:00)

CONCERN: PREFER AUTOMATED APPLIANCES TO ADDITIONAL INFORMATION (THERMOSTAT) :[L21]

She wanted to have an automatic thermostat and didn't care about monitoring adjustments.

She wanted to know if there was a change in heart rate, blood pressure, skin, short-term memory, things that measure activity, commitments, ability to concentrate, laughing.

She knows she can't recall stories and jokes so doesn't need that monitored.

Doesn't have a digital camera or pets, doesn't smoke or crack her knuckles

She thinks time spent cooking, interacting with people, sleep routines, would all be relevant to detecting significant health changes.

IDEA VARIABLE: IDLE TIME (FINDING MORE) :[L21]

Interesting perspective on idle time: `"I don't think we have enough idle time."` :[L21]

IDEA VARIABLE: IMPULSIVENESS (BEING MORE IMPULSIVE) :[L21]

Interesting perspective on impulsiveness: `"We should always stay impulsive."` :[L21]

Resorted on physical activity.

ID: [L20] & [L22]

Date: 6/16/04, 11:05-12:25PM (80 minutes)
Participant type: Participant, through community group visit
Demographics: [L22] is over 70, Louise is about 65
Location: Community center
Audio file: DM100018, DM100019

- Shown 5 Displays: Journal, Snoop, Mirror, Place, Performance

GENERAL REACTION: PREFER SOLUTIONS TO INFORMATION (-) :[L22]

- Already restricted life, many things don't apply
- Interested in a ready-made diet solution

GENERAL REACTION: POSITIVE ABOUT TRACKING WITH PROMPTING (+/-) :[L20]

- Interested in better managing her social commitments
- Notes that her routine has changed with a move to a new community
- Interested in how media leads to photography project

Technology: [L22] has a cell phone; Louise has a cell phone and digital camera
Work: [L22] has worked as a dietician and is now focusing on writing children's fiction (unpublished as yet); Louise is a polarity therapist

[What I monitor] (0:01:25)

CURRENTLY TRACKING: BLOOD SUGAR, CALORIC INTAKE, AMOUNT OF INSULIN :[L22]

[L22] is diabetic. She tracks her blood sugar, her calorie intake, the amount of insulin she's taking, and her weight. She writes down what she eats, but it's not easy for her to do. She takes a blood sugar count in the morning and before each meal.

IDEA VARIABLE: CALORIES :[L22]

"I would like to see some sort of computer where you stick it [food] in and see what it is [calories, etc.]." :[L22]

"This morning I had a quarter of a melon and an ensure, which is 350 calories. If I had looked sooner, I would have gotten something with less calorie count, because I don't need calories." :[L22]

She knows that activity affects blood sugar, but does not track it.

Louise loves photography and takes nature and journalistic photos.

[Journal](0:07:20 second file)

POSITIVE REACTION: SNACKING :[L22]

[L22] noted the snacking example as something she would want to track, related to her diabetes.

POSITIVE REACTION: MEDIA :[L22]
IDEA RELATIONSHIP: RELATED INFORMATION FROM MULTIPLE MEDIA SOURCES :[L22]
NEUTRAL REACTION: MEDIA AND POINT IN LIFE :[L22]

She also is interested in tracking the things she learns from the media, particularly for material for her books. She also notes that she'll hear about something on a particular day and time and then shortly afterwards, hears about it again from some where else, so she would like to be able to link and track the sources. She doesn't think, however, that she would be looking at the data to understand "what was I watching/listening to in January"; instead she would want to do specific searches.

Positive reaction: places visited :[L20]
Positive reaction: media :[L20]
Idea use: source of media that inspires trip or activity, trip or activity, and photos from trip or activity :[L20]

Louise liked "places visited" and wanted to connect it with media and her photographs. She often gets ideas for what to photograph (in her community) from television. She would like to record where the idea comes from and relate it the location she went to and the photos she took.

> "If they talk about sunrise in Boston, I might take a note of that and then some morning take the camera and go there, take pictures of the sunrise, and [put an article together for the local paper]." :[L20]

Positive reaction: water-drinking :[L20]

Louise liked the water-drinking and memorable moment examples.

> "I need to keep track of water drinking because I don't drink enough. If that could be done easily." :[L20]

Positive reaction: people encountered :[L20]
Positive reaction: memorable moments :[L20]
Idea use: people encountered and the stories they tell :[L20]

> "I like the idea of keeping track of memorable moments, if there was something to do that easily, because I know sometimes when I'm roaming around with the camera, I will ask people for their favorite places and sometimes I will meet some fascinating people. I'll have an interesting 10-15 minute conversation and it would be nice to keep track of that and those people." :[L20]

[Snoop] (0:15:50)
Positive reaction: eating behaviors :[L22]
Positive reaction: opportunities for exercise :[L22]
Positive reaction: work habits :[L22]
Idea use: prepared meals :[L22]
Procedure: interest in solution rather than tracking (eating behaviors) :[L22]

[L22] liked eating behaviors, opportunities for exercise, and work habits. She really wishes that there could be a tailored meal plan or prepared meals for her to meet her strict dietary needs. She feels she knows what she shouldn't do and triggers her eating, but wants a structured solution.

Neutral reaction: social awareness, limited social life :[L22]

(0:18:36) [L22] doesn't feel she needs help with social awareness because she "doesn't have a social life". She is not close to her extended life and is not married or have friends.

Idea variable/use: opportunities to eat, reminders when to eat :[L20]

Louise needs help finding opportunities to eat meals. She believes it would help to have a better sense of her schedule and thinks an alarm notifying her of when to eat would help.

> "I tend to run out, realizing I can make it there in such and such a time, if I just don't eat." :[L20]

Positive reaction: opportunities for exercise :[L20]

> "I think it would be interesting to have someone report on opportunities for exercise. I think I'm pretty good with that, but was better a few years ago." :[L20]

Idea use: finding replacements for activities or locations that triggered routines and exercise on move to new location :[L20]

Louise noted that her daily routine interrupted when she moved to a new location. In her previous location, she had a reason to get up early in the morning to go to the beach and her routine was fairly consistent. Now her routine is highly variable and she may get less exercise.

[Mirror] (0:24:25)

BENEFIT: RECOGNIZING LAPSES IN SELF-CARE :[L22]

IDEA USE: REFLECTION TO EVALUATE WHETHER TAKING CARE OF HAIR, MAKE-UP, AND APPEARANCE AS WELL AS IN PAST :[L22]

[L22] is not so much interested in clothing choices, but does believe that seeing one's reflection from a year ago (or some time ago) could provide an objective insight about whether she is letting herself go in terms of hair, make-up, and other visible signs of health and hygiene.

> "It would be interesting to see what you looked like a year ago and then a projection of what you might look like in the future. You could see the change and then correct it. Sometimes you don't pay attention to your hair and stuff. Our eyes look out and we don't see stuff – it would be help to see how other people see you." :[L22]

NEUTRAL REACTION: MEMORABLE MOMENTS – LIFE NOT EVENTFUL ENOUGH :[L22]

NEUTRAL REACTION: PEOPLE ENCOUNTERED – LIFE NOT EVENTFUL ENOUGH :[L22]

[L22] wasn't interested in the events or people encountered because she didn't think her life was eventful enough.

POSITIVE REACTION: PEOPLE ENCOUNTERED :[L20]

IDEA VARIABLE: SOCIAL COMMITMENTS :[L20]

On the other hand, Louise thought the people encountered would be very important to her. She keeps an appointment book and has to refer to it to avoid missing her social commitments; she has frustrated friends in the past.

[L22] proposed that Louise's schedule could be displayed for her in the morning mirror while she brushes her teeth and Louise agreed that would be helpful if it could be easily done

[Place] (0:29:04)

POSITIVE REACTION: LOW POINTS AND HIGH POINTS :[L20]

IDEA RELATIONSHIP: MOOD AND BEING OUTDOORS :[L20]

IDEA RELATIONSHIP: CHANGES TO HOME THAT COINCIDE WITH HIGHER MOOD (E.G., I'M HAPPY AT HOME WHEN…) :[L20]

BENEFIT: INVESTIGATING HOW TO ALTER ENVIRONMENT OR SITUATION TO IMPROVE EXPERIENCE :[L20]

Louise liked the low points and high points place example. She is happy when she is around a lot of people and can get "a little slumpy" when she is home alone. She knows that being outdoors improves her mood. She's tried making modifications to her home to improve her mood, such as adding plants, and is now considering having a cat.

(0:35:55)

IDEA RELATIONSHIP: BLOOD PRESSURE AND SALT INTAKE :[L22]

[L22] mentioned that she would like to monitor blood pressure and salt intake. She and Louise discussed how blood pressure readings in the clinic can be wrong because of "white coat syndrome."

POSITIVE REACTION: BLOOD PRESSURE AND FEELINGS OF STRESS, BLOOD PRESSURE AND EVENTS/ACTIVITIES DURING THE DAY :[L22]

IDEA VARIABLE: CHOLESTEROL :[L22]

[L22] liked the idea of associating blood pressure with stress. She noted that she wasn't highly stressed out, but liked the idea of associating it with events or activities during the day. She also noted cholesterol as something to track.

[Performance] (0:38:20)

POSITIVE REACTION: PROJECTS – IMPROVEMENT ON PHOTOGRAPHY :[L20]

Louise noted that she already likes to monitor the improvement on her photography. She noted there was an improvement, and then a drop for a little bit when she switched to digital photography.

> "Sometimes there is a progression, and then you change the situation, and it drops for a bit then improves." :[L20]

IDEA VARIABLE: WEIGHT – IMPROVEMENTS :[L22]

[L22] was interested in tracking improvements in weight.

[Sorting] (0:39:52) (Louise)

IDEA RELATIONSHIP: SNORING, FREQUENCY, SLEEPING POSITION, TIME OF NIGHT :[L20]

> "Snoring yes, I didn't realize I snore, but I've been on vacation with some people and one woke me up one night and said turn over!" :[L20]

She thought it would be interesting to see how snoring related to sleeping position and time of night and whether it happens all the time.

POSITIVE REACTION: NUMBER OF PHOTOGRAPHS TAKEN AND SEASON :[L20]

> "Number of digital photographs taken, that's a definite yes. You can track that…"

She liked the idea of graphing the data against time, and examining differences with seasons.

> "Use of microwave, I don't need to track that, I use it when I need to use it." :[L20]

> "Smoking. I don't smoke. Alcohol drinking is so little, it doesn't need." :[L20]

> "Headaches, I don't get them." :[L20]

IDEA RELATIONSHIP: NUMBER OF MOLES AND AGE :[L20]

> "Skin changes – I'm getting a lot of moles, so that might be interesting. That seems to be an old thing <laughs>":[L20]

> "Use of space, definitely, I'd like to find a better way to use space." :[L20]

> "Gullibility, hmmm! Yeah, I can be gullible." :[L20]

> "Refrigerator open/close. Nah, doesn't matter." :[L20]

When she gets to "ability to recall jokes and stories, she laughs and says she has a tendency to remember the set-up or the punchline, but not both.

She notes that her computer already tracks her use of programs and thinks it works well.

INVESTIGATION: SOCIAL COMMITMENTS - OVERCOMMITTING, FREQUENCY OF CANCELLATIONS, FRIENDS' FRUSTRATION :[L20]

Louise had a high level of interest in tracking social commitments. She often over-commits even though she keeps an appointment. She has had to cancel on people.
She would like to know how often she does it and who she is frustrating.

She spends more time in the car than in the house.

IDEA VARIABLE: PEOPLE ENCOUNTERED INTO NEW FRIENDS, OLD FRIENDS, AND ACQUAINTANCES :[L20]
She would like to break down people encountered by new friends, old friends, and acquaintances.

IDEA USE: TUNE INTO NEWS EVENTS THAT ARE IMPORTANT TO FRIENDS :[L20]
She would like something to remind her to check on news, particularly news events that are important to her friends, so she can understand their references.

PROCEDURE: SURPRISE ABOUT NUMBER OF VARIABLES SELECTED :[L20]

> “I’m building a big stack there.” :[L20]

IDEA USE: VARIATION FROM ROUTINE TO TRY TO ESTABLISH A MORE STRUCTURED ROUTINE :[L20]

> “Variation from routine – it would definitely be good to have some help with that. I don’t have a standard routine, I go where the mood takes me.” :[L20]

IDEA VARIABLE: TIME SPENT WORKING, TIME SPENT MAKING PHONE CALLS :[L20]
Working and making phone calls affects the time she has available.

Louise commented on the interesting intergenerational communication that can take place on selecting “family knowledge,”

> “I have recently discovered that my mom, who is in her 90s, has told my daughters a lot of stuff that she never told me. Things she did as a teenager, risky things <laughs>” :[L20]

IDEA RELATIONSHIP: MOOD AND BAROMETRIC PRESSURE :[L20]
Louise was definitely interested in mood and noted that barometric pressure affects mood.

IDEA VARIABLE: TIME SPENT EATING OUT :[L20]
She cooks very little, so was unsure how to classify the “time spent cooking” card, but noted that time spent eating out may be more important.

IDEA USE: TRACKING MULTITASKING TO LEARN HOW TO ORGANIZE TASKS :[L20]
CONCERN: DIFFICULT TO CHANGE NATURE (IMPULSIVENESS) :[L20]
She thought it would be good to look out how to organize her tasks, how to better multitask, but she knows that her nature is to be more impulsive.

> “Laughing. I don’t think I laugh enough. Used to, but don’t think I do now.” :[L20]

INVESTIGATION: SNACKING AND SPENDING :[L20]
Related to tracking snacking, Louise says she once kept track of her purchasing of coffee and donuts at Dunkin Donuts and found she was spending $20 a week; as a result, she decided to stop making those frequent stops.

ID: [L23] & [L24]

Date: 6/16/04, 12:27-1:12PM (45 minutes)
Participant type: Participant, through community group visit
Demographics: Women, 70s
Location: Community center
Audio file: DM100020

- Shown 4 Displays: Journal, Snoop, Place, Voicemail

GENERAL REACTION: MOSTLY NEUTRAL, INTEREST IN SOME INVESTIGATIONS (-/+) :[L23]

- Interest in receiving observations about her social behaviors
- Would find information about the activities of her adult children intrusive

GENERAL REACTION: AMBIVALENT, INTEREST IN SOME INVESTIGATIONS(-/+) :[L24]

- Observes that her journaling has corresponded with times when she was down or bored
- Would find information about the activities of her adult children intrusive
- Believe much of her life is too simplified, or doesn't want to make changes, to justify tracking
- Would like to understand fluctuations in her mood, mental sharpness

Technologies: [L23] tried using a cell phone, but didn't use it; they both own personal computers

POSITIVE REACTION: REFLECTION :[L24]
IDEA USE: REFLECTION FROM YEAR AGO TO JOG MEMORY ABOUT WHAT WAS GOING ON THEN :[L24]
Before the interview, [L24] had seen a screen shot of the mirror example and had noted that seeing what she was wearing, as it relates to the weather, from a year ago might aid her in recalling events. She said it was difficult to remember what was happening at the same time last year and this would provide a helpful prompt.

[What I monitor] (0:00:50)
IDEA USE: TRACKING (JOURNALING) AS AN INDICATOR OF MOODS :[L24]
[L24] has kept a journal at times in the past, but she noticed it was only when she was down or bored, and then something exciting would happen and she wouldn't need it anymore.

[Journal] (0:05:45)
POSITIVE REACTION: SNACKING :[L24]
POSITIVE REACTION: WATER DRINKING :[L24]

> "I only got interested in what you were saying when you got to food and how much water. I do try to track how much water I drink right now and I'd be interested in food." :[L24]

IDEA USE: HELPING TO SCHEDULE WATER DRINKING TO AVOID AWKWARD BATHROOM TRIPS :[L24]
Right now, [L24] does not write down how much water she drinks, but she tries to drink 4-6 glasses starting at night. Scheduling the water is important because she doesn't want to have to go to the bathroom during the day when she's out.

POSITIVE REACTION (MILD): MEDIA – BOOKS THAT HAVE READ, ONLY INTERESTING :[L23]
[L23] would note the places visited and the people encountered, but she does not care about snacking or drinking water. She watches TV in the background while working on the computer, she occasionally reads newspapers or watches movies, but she reads books constantly. She thinks it might be interesting to track what she reads over time, but isn't sure it would be important.

[L23] tracks genealogy on the computer.

[Snoop] (0:13:10)

POSITIVE REACTION: SOCIAL AWARENESS :[L23]
IDEA VARIABLE: GOOD LISTENING SKILLS :[L23]

[L23] expressed an interest in social awareness, which prompted [L24] to agree.

"I'd like someone to observe me while I am interacting with other people and tell me what I'm doing right or wrong. At which point I could try to correct it. I do try to make eye contact and listen, not just hear the words, but understand the words. I would like someone to watch me not so much criticize, but give me feedback" :[L23]

POSITIVE REACTION: SOCIAL AWARENESS :[L24]
POSITIVE REACTION: WORK HABITS :[L24]

[L24] expressed interest in social awareness and work habits, noting that hearing what [L23] had to say influenced her interest in the former.

NEGATIVE REACTION: EATING HABITS, SENSITIVE ABOUT WEIGHT :[L24]
PROCEDURE: CONTRADICTION - PARTICIPANT LIKED SNACKING, BUT THOUGHT EATING BEHAVIORS WOULD MAKE HER FEEL SENSITIVE ABOUT HER WEIGHT :[L24]
NEGATIVE REACTION: OPPORTUNITIES FOR EXERCISE, WHAT SHE DOES ALREADY SHOULD BE ENOUGH :[L24]

[L24] doesn't want to anyone to tell her about her eating habits because she is sensitive about her weight.

"Opportunities for exercise is too much effort. I walk everywhere because I don't have a car and I feel that covers it." :[L24]

[Place] (0:18:10)

NEUTRAL REACTION: ACTIVITY PATTERNS :[L24]
CONCERN: MOST OF BEHAVIOR IS WHAT NEEDS TO BE DONE, LITTLE CHOICE INVOLVED (ACTIVITY PATTERNS) :[L24]

Patterns in space –

"I don't think it would affect my life, I'm just doing what I must do." :[L24]

NEUTRAL REACTION: MEDICATION ADHERENCE :[L24]
CONCERN: MORE INTERESTED IN SOLUTION TO PROBLEM (REMINDER) THAN TRACKING :[L24]

[L24] wasn't sure what she could get from knowing about medication adherence with respect to place, though she noted that she does forget a pill she is scheduled to take at midday. When we discussed the idea that she might discover she was outside when she was missing the pill, she suggested that she should leave it at Papa Gino's, because that's where she consistently goes to lunch. She could imagine having a reminder for the pill, but it is not clear whether she feels she would benefit from reflection on her medication patterns.

POSITIVE REACTION: LOW POINTS/HIGH POINTS :[L24]
POSITIVE REACTION: LOW POINTS/HIGH POINTS :[L23]

Both [L24] and [L23] were interested in the idea of tracking mood, presented to them in the context of "place." (0:22:10).

IDEA RELATIONSHIP: MOOD AND HORMONE CYCLES (AFTER MENOPAUSE) :[L24]
IDEA RELATIONSHIP: MOOD AND SPECIAL EVENTS :[L24]
IDEA RELATIONSHIP: MOOD AND PHONE CALLS :[L24]

[L24] says she is trying to figure out the fluctuations in her mood. She feels like it has something to do with her period, even though she is post-menopausal. She notes though that the going on a "field trip" with friends to MIT the previous day had given her an incredible, and unexpected, mood boost. She suggested that phone calls might be associated with mood and that when situations are

uncertain or unsettled causes her to worry, and this is particularly dependent on the actions of family members.

[L23] noted that her worrying about her daughter would not help either of them, so she tries not to worry.

[Voicemail] (0:26:25)

NEGATIVE REACTION: VOICE MAIL :[L23]:[L24]

CONCERN: HAVING INFORMATION FORCES INVOLVEMENT (VOICE MAIL) :[L24]

Both [L24] and [L23] react strongly to having access to the details of their children's lives – they don't want to have that level of involvement. They would rather their children tell them things directly. It's important to [L24] not to over-mother and knows her family is not as close as some, but that's how she likes it.

> "I raised my children to grow-up, which means don't consult, don't call, ask for money, solve your own problems just like dad and I did. Let me know if anything is *really* wrong, I want to know that, but you're not a child anymore. They have secrets from me, but then I had secrets from my parents too, and from my in-laws." :[L24]

NEGATIVE REACTION: VOICE MAIL :[L23]

CONCERN: TOO MUCH DETAIL (VOICE MAIL) :[L23]

[L23] enjoys being close to her family, but perceives this level of detail as being too much. They interpret the first example ("your granddaughter only took 3000 steps this week") initially as more of progress update, like baby's first step, that would only really be of interest to the mom. When asked about it in terms of "your family member was watching TV all week and didn't get exercise," both imagine that they would tell the mother to get the kid outside.

[Sorting] (0:31:25)

[L24] sorts for mood and [L23] sorts for family relationships

IDEA RELATIONSHIP: MENTAL SHARPNESS BY DAY, PRODUCTIVITY :[L24]

> "Mental sharpness. That's true. Why do some days I want to write 3 stories for the paper and other days don't want to look at it?" :[L24]

IDEA RELATIONSHIP: BAROMETRIC PRESSURE AND MOOD :[L24]

> "Barometric pressure has a lot to do with your mood." :[L24]

IDEA RELATIONSHIP: EMAIL (CONTENT, SENDER, INTENTION) AND MOOD :[L24]

[L24] initially wasn't interested in email sent/received, but [L23] noted that particular emails could affect her.

> "Email [can affect me]… The content of the email, who sent it and what the intention was." :[L23]

Both women agreed that chain letters and forwarded mail really annoys them.

CONCERN: NO NEED TO TRACK WHEN THERE IS LITTLE CHOICE/LIFE IS SIMPLIFIED (CLOTHING CHOICES) :[L24]

[L24] did not select "clothing choices" because she has a simple wardrobe.

[L23] interpreted "use of computer application" in terms of a genealogy program.

IDEA RELATIONSHIP: ACCOMPLISHING SOMETHING NEW WITH COMPUTER AND MOOD :[L24]

[L24] chose "use of the computer application" as it affects her mood,

> "I honestly – when I remember something and I accomplish something, and it works, I get as excited as when someone invites me on a date." :[L24]

PROCEDURE: UNFAMILIAR TERMINOLOGY, GLUCOSE AND HORMONES :[L23] :[L24]

[L23] and [L24] didn't know what blood glucose or hormones levels referenced.

IDEA RELATIONSHIP: CLUTTER AND MOOD :[L24]

[L24] felt that when her space becomes too cluttered, and things aren't thrown out, it affects her mood.

IDEA RELATIONSHIP: TAKING PHOTOS AND MOOD :[L24]

[L24] noted that she is delighted with the novelty of a digital camera and so it taking a lot of photos. She's not sure whether it will be associated with mood

[L23] and [L24] don't think they snore.

> "I don't hear myself snoring <laughs>" :[L24]

ID: [L25] & [L26]

Date: 6/21/04, 10:30-12:01PM (90 minutes)
Participant type: Participant, through email announcement
Demographics: Couple, 70s
Location: House_n
Audio file: DM100025

- Shown 4 Displays: Journal, Snoop, Voicemail, Years

GENERAL REACTION: MORE FOCUSED ON SOLUTIONS, INTEREST IN SOME INVESTIGATIONS (-) :[L25]:[L26]

- Already doing detailed tracking of expenses
- Procedural challenges: differentiating "this applies to me" from "I want to track"
- Interesting suggestion that tracking will cause self to lose faith that change will occur
- Interest in preserving family knowledge – connection with understanding other family members' medical histories

Technologies: Digital camera, laptop, but no cell-phone

[what I monitor] (0:01:50)

CURRENTLY MONITORING: WEIGHT, CALORIES, CARBS :[L26]:[L25]

They track weight, calories, and carbohydrates, but don't write that information down.

[L26] has just scanned 2000 family photos slides.

CURRENTLY TRACKING: SPENDING BY CATEGORY :[L26]:[L25]

They keep detailed records of their expenses and put together a yearly report broken down into 40 categories, including eating out, travel, food, gifts, taxes, miscellaneous household stuff, etc. They have been tracking since [L26] retired in 1988. They sometimes adjust what they spend based on this report.

"We know that Satan is in restaurants." :[L26]

CURRENTLY TRACKING: ACTIVITIES ON VACATION :[L26]:[L25]

They keep a travel diary
"We make notes of where we traveled or what we did on that day." ([L25])

CURRENTLY TRACKING: VACATIONS, DESTINATION, JOURNEY TYPE, AND LENGTH OF STAY :[L26]:[L25]

[L25] has kept her wall calendars since 1958 and is planning to go back through them to understand where and when they traveled.

"That's what we're doing now, we want to go back and figure out where we went and how we went and how long we stayed. Tracking our life over the years" :[L26] **(0:05:50)**

CONCERN: TRACKING SUGGESTS LACK OF CONFIDENCE THAT BEHAVIOR CHANGE WILL WORK (BLOOD SUGAR) :[L26]

(0:08:00) [L26] has been diagnosed as a diabetic, just over the borderline, and was encouraged to start track his blood glucose, but would rather take a "positive" approach by believing that altering his diet and exercise will work. His wife [L25] appears skeptical and notes that at some point they may need to track it.

"I know it's going to work. In a lot of cases in health, attitudes are important." :[L26]

[Journal] (0:11:10)

CURRENTLY TRACKING: MOVIES WATCHED :[L26]:[L25]

They write down the name of the videos they rent in order to remember what they've seen, and also record the expense.

CURRENTLY MONITORING: WATER DRINKING :[L26]:[L25]
They are monitoring water drinking, but don't currently write it down.

NEUTRAL REACTION: SNACKING, SUFFICIENTLY CONSCIOUS :[L26]:[L25]
They don't have an interest in tracking snacking, because they believe they are sufficiently conscious of their eating. They noted that they've already been given a list of things to eat by the diabetes dietician and after a few days decided they could manage it themselves without having to refer to the list.
IDEA VARIABLE: WHEN KIDS VISIT :[L26]
They would like to record when their kids visit and so sometimes record that kind of thing on their wall calendars.

IDEA VARIABLE: TIME SPENT ON COMPUTER :[L26]:[L25]
They would like to track hours spent on the computer.

[Snoop] (0:19:00)
PROCEDURE: DIFFERENTIATING "IT APPLIES TO ME" FROM "I WANT TO TRACK THIS" :[L26]:[L25]
They were able to identify with some of the examples, noting that [L25] puts her hands in her pockets a lot and that they liked the idea of lifting weights while watching TV.

PROCEDURE: FEEDBACK ON DESIRED SOLUTIONS RATHER THAN TRACKING :[L26]:[L25]
[L26] would like someone who is a financial expert to advise them on spending.

They felt they already do many of the social awareness examples and so don't need help.

IDEA VARIABLE: TIME SPENT HELPING OTHER PEOPLE ON COMPUTER PROGRAMS :[L26]:[L25]
(0:25:30) [L26] could imagine tracking time spent helping other members of his community newspaper group.

CURRENTLY TRACKING: GENEALOGY AND FAMILY PHOTOS :[L26]:[L25]
(0:27:40) Genealogy is of great importance – they have traveled to locations where their family lived and tracked down extended family members; they collect stories and family facts; their children (in their 40s) aren't as interested in the family, but [L26] and [L25] recognize the importance of preserving it, particularly as family members pass away.

(0:37:00) Scanning and labeling photographs is a significant project for them right now. They find value in digitizing fading photos and presenting them back to family members. They note that their ancestors went to great trouble to get a family photograph taken and now they are able to preserve it. They send CDs with photos that contain individuals whom they can't identify to extended family members for labeling, but sometimes encounter problems with family members who don't have computer access.

[Voicemail] (0:41:00)
NEUTRAL REACTION: ACTIVITY INFORMATION FROM FAMILY MEMBERS :[L26]:[L25]
IDEA USE: TRADING HEALTH HISTORIES WITH EXTENDED FAMILY :[L26]:[L25]

They didn't have an interest in getting current activity information from family members but were interest in trading health histories. They noted that talking about medical histories was never done as part of their regular family life growing up, so there is a lot of missing information.

They are interested in the health history of ancestors and current family members – through their search, found out that several extended family members also suffer from abdominal aneurisms, which [L26] experienced. [L25] also says she would be fascinated to know about the physical characteristics of ancestors.

NEUTRAL REACTION: SLEEP/WAKE SCHEDULES, TIME IS SO FLEXIBLE :[L26]:[L25]
They wouldn't be interested in tracking their sleep/wake schedules, because their time is so flexible.

IDEA VARIABLE: TIME SPENT SLEEPING, CONCERN NOT GETTING ENOUGH :[L26]
IDEA USE: PRESENTING INFORMATION TO DOCTOR (TIME SPENT SLEEPING) :[L26]
[L26] can imagine presenting evidence for how much time they are spending sleeping to a GP, because he doesn't feel he's getting enough.

IDEA RELATIONSHIP: TIME SPENT READING, TIME SPENT WATCHING TV, TIME SPENT USING THE COMPUTER :[L26]
IDEA VARIABLE: OVERLAPPING MEDIA USE (READING A BOOK WHILE WATCHING TV) :[L26]
[L26] would like to know how much time he is spending reading books, watching TV, and using his computer, and how much of that time overlaps (reading a book while watching TV).

[Years] (0:50:21)
NEUTRAL REACTION: SHORT-TERM MEMORY OVER YEARS :[L26]
IDEA RELATIONSHIP: STIMULATING ACTIVITIES AND MEMORY :[L26]
[L26] says that his short-term memory, and those in his age group, is affected and that he is concerned about Alzheimer's, but wasn't sure how it could be tracked. I presented the idea of playing a game or taking a test. He said he believed that doing stimulating activities such as reading and doing crossword puzzles, and working on the computer, affects his memory. He didn't seem opposed to tracking whether these stimulating activities have an affect on memory score, but didn't articulate that explicitly.

IDEA USE: COUPLES WORK TOGETHER TO RECALL EVENTS AND INFORMATION, HOW CAN THAT COOPERATION BE EVALUATED? :[L26]
He did note that often a couple works together to recall events or information.
NEUTRAL REACTION: TIME SPENT COOKING, NO INTEREST :[L25]
NEUTRAL REACTION: TIME SPENT WITH FRIENDS, NO INTEREST :[L25]
[L25] did not think she would want to track time spent cooking or time spent with friends.

[Sorting] (0:55:10) – together (no re-sort)
