# Supplementary material for: To Track or Not to Track: User Reactions to Concepts in Longitudinal Health Monitoring
Source: J Med Internet Res. 2006 Dec 7;8(4):e29. doi: 10.2196/jmir.8.4.e29 (PMC1794006; doi:10.2196/jmir.8.4.e29)
Supplement: Supplementary file 3 [file jmir_v8i4e29_app3.html]

Sorting Exercise


# Sorting Exercise

Please go through each item and check whether you, personally, would like to track it over time. Please select the first select circle for "No, I would not want to track this," the second select circle for "Maybe or Unsure," and the third select circle for "Yes, I would want to to track this." During the interview, I will ask you to tell me what you are selecting and why.

*The selections you make are not saved or transmitted.*

|  |  |  |  |
| --- | --- | --- | --- |
| **Would you, personally, want to track this?** | **no** | **maybe** | **yes** |
| ability to concentrate |  |  |  |
| ability to recall jokes and stories |  |  |  |
| adjustments to the thermostat |  |  |  |
| alcohol drinking |  |  |  |
| awareness of time |  |  |  |
| barometric pressure |  |  |  |
| bathroom trips |  |  |  |
| blood sugar (glucose) |  |  |  |
| blood pressure |  |  |  |
| clothing choices |  |  |  |
| coffee/tea drinking |  |  |  |
| commitments |  |  |  |
| community traffic congestion |  |  |  |
| conversation turn-taking with family |  |  |  |
| correspondence with friends/family |  |  |  |
| emails sent/received |  |  |  |
| foot steps |  |  |  |
| grip strength |  |  |  |
| gullibility |  |  |  |
| headaches |  |  |  |
| heart-rate |  |  |  |
| hormone levels/cycles |  |  |  |
| how and when you use a computer application |  |  |  |
| how much you know about your friends and family |  |  |  |
| how well you recognize people's moods and reactions |  |  |  |
| idle time |  |  |  |
| impulsiveness |  |  |  |
| knuckle-cracking |  |  |  |
| laughing |  |  |  |
| mood self-rating |  |  |  |
| multitasking |  |  |  |
| muscle tone |  |  |  |
| news watched/read |  |  |  |
| night vision |  |  |  |
| number of digital photos taken |  |  |  |
| people encountered |  |  |  |
| pet activity levels |  |  |  |
| pitch perception (hearing) |  |  |  |
| posture |  |  |  |
| raised voices |  |  |  |
| refrigerator open/close |  |  |  |
| response time |  |  |  |
| short term memory |  |  |  |
| skin changes |  |  |  |
| smoking |  |  |  |
| snacking |  |  |  |
| snoring |  |  |  |
| sports performance |  |  |  |
| spouse's mood |  |  |  |
| time at which you go to sleep |  |  |  |
| time spent cooking |  |  |  |
| time spent in the car |  |  |  |
| time spent with friends |  |  |  |
| tossing and turning |  |  |  |
| touch perception (touch sensitivity) |  |  |  |
| trips to the grocery |  |  |  |
| TV watching |  |  |  |
| use of microwave |  |  |  |
| use of space |  |  |  |
| variation from routine |  |  |  |

  
  


---

## Tracking with a Goal in Mind

Please select from the list below something that you would like to better understand and possibly change or impact. For example, you might want to understand the highs and lows of your mood and make changes that will result in fewer "down" days.

|  |  |
| --- | --- |
|  | eating choices |
|  | family relationships |
|  | mental sharpness |
|  | mood |
|  | physical activity |
|  | stress |

Now, keeping what you just selected in mind, what would you want to track to understand that thing? For example, if you chose mood, what would you want to track to understand the highs and lows of your mood? What information could you use to impact your mood? Remember to only check items that you, personally, would want to track.

|  |  |  |  |
| --- | --- | --- | --- |
| **Would you, personally, want to track this?** | **no** | **maybe** | **yes** |
| ability to concentrate |  |  |  |
| ability to recall jokes and stories |  |  |  |
| adjustments to the thermostat |  |  |  |
| alcohol drinking |  |  |  |
| awareness of time |  |  |  |
| barometric pressure |  |  |  |
| bathroom trips |  |  |  |
| blood sugar (glucose) |  |  |  |
| blood pressure |  |  |  |
| clothing choices |  |  |  |
| coffee/tea drinking |  |  |  |
| commitments |  |  |  |
| community traffic congestion |  |  |  |
| conversation turn-taking with family |  |  |  |
| correspondence with friends/family |  |  |  |
| emails sent/received |  |  |  |
| foot steps |  |  |  |
| grip strength |  |  |  |
| gullibility |  |  |  |
| headaches |  |  |  |
| heart-rate |  |  |  |
| hormone levels/cycles |  |  |  |
| how and when you use a computer application |  |  |  |
| how much you know about your friends and family |  |  |  |
| how well you recognize people's moods and reactions |  |  |  |
| idle time |  |  |  |
| impulsiveness |  |  |  |
| knuckle-cracking |  |  |  |
| laughing |  |  |  |
| mood self-rating |  |  |  |
| multitasking |  |  |  |
| muscle tone |  |  |  |
| news watched/read |  |  |  |
| night vision |  |  |  |
| number of digital photos taken |  |  |  |
| people encountered |  |  |  |
| pet activity levels |  |  |  |
| pitch perception (hearing) |  |  |  |
| posture |  |  |  |
| raised voices |  |  |  |
| refrigerator open/close |  |  |  |
| response time |  |  |  |
| short term memory |  |  |  |
| skin changes |  |  |  |
| smoking |  |  |  |
| snacking |  |  |  |
| snoring |  |  |  |
| sports performance |  |  |  |
| spouse's mood |  |  |  |
| time at which you go to sleep |  |  |  |
| time spent cooking |  |  |  |
| time spent in the car |  |  |  |
| time spent with friends |  |  |  |
| tossing and turning |  |  |  |
| touch perception (touch sensitivity) |  |  |  |
| trips to the grocery |  |  |  |
| TV watching |  |  |  |
| use of microwave |  |  |  |
| use of space |  |  |  |
| variation from routine |  |  |  |
