# Supplementary material for: To Track or Not to Track: User Reactions to Concepts in Longitudinal Health Monitoring
Source: J Med Internet Res. 2006 Dec 7;8(4):e29. doi: 10.2196/jmir.8.4.e29 (PMC1794006; doi:10.2196/jmir.8.4.e29)
Supplement: Supplementary file 4 [file jmir_v8i4e29_app4.pdf]

Construct	Yes - General % (N=21)
correspondence with friends/family	81
heart-rate	76
time at which you go to sleep	71
short term memory	71
muscle tone	71
pitch perception (hearing)	71
use of space	71
hormone levels/cycles	67
commitments	67
snacking	67
multitasking	67
laughing	67
ability to concentrate	62
blood pressure	62
variation from routine	62
blood sugar (glucose)	62
posture	62
response time	57
time spent with friends	57
how well you recognize people's moods and reactions	52
coffee drinking	52
night vision	52
skin changes	52
people encountered	52
time spent cooking	52
grip strength	52
foot steps	48
conversation turn-taking with family	48
impulsiveness	48
gullibility	48
emails sent/received	48
time spent in the car	48
mood self-rating	43
how much you know about your friends and family	43
trips to the grocery	43
headaches	43
raised voices	43
ability to recall jokes and stories	43
touch perception	43
idle time	43
tossing and turning	43
how and when you use a computer application	43
sports performance	43
clothing choices	38
spouse's mood	38
barometric pressure	38
number of digital photos taken	38

TV watching	38
news watched/read	33
awareness of time	33
bathroom trips	33
refrigerator open/close	33
alcohol drinking	29
adjustments to the thermostat	29
snoring	24
community traffic congestion	19
pet activity levels	14
use of microwave	10
knuckle-cracking	0
smoking	0

Construct	Yes - Investigation % (N=21)
time at which you go to sleep	80
ability to concentrate	70
idle time	70
hormone levels/cycles	65
heart-rate	60
commitments	60
variation from routine	55
blood pressure	55
snacking	50
awareness of time	50
mood self-rating	45
short term memory	45
impulsiveness	45
blood sugar (glucose)	45
posture	45
time spent with friends	45
people encountered	45
TV watching	45
muscle tone	40
multitasking	40
time spent in the car	40
foot steps	40
headaches	40
how well you recognize people's moods and reactions	35
conversation turn-taking with family	35
time spent cooking	35
laughing	35
response time	35
trips to the grocery	35
raised voices	35
coffee drinking	35
spouse's mood	35
alcohol drinking	35
tossing and turning	30
news watched/read	30
barometric pressure	30
refrigerator open/close	30
grip strength	30
pitch perception (hearing)	25
ability to recall jokes and stories	25
how and when you use a computer application	25
adjustments to the thermostat	25
community traffic congestion	25
how much you know about your friends and family	20
sports performance	20
use of space	20
night vision	20

gullibility	20
touch perception	15
clothing choices	15
bathroom trips	15
pet activity levels	15
correspondence with friends/family	10
skin changes	10
emails sent/received	10
snoring	10
use of microwave	10
knuckle-cracking	5
number of digital photos taken	0
smoking	0
